# A Comprehensive Review on Thiophene Based Chemosensors

**DOI:** 10.1007/s10895-021-02833-x

**Published:** 2021-10-08

**Authors:** Rikitha S. Fernandes, Nitinkumar S. Shetty, Priyanka Mahesha, Santhosh L. Gaonkar

**Affiliations:** Department of Chemistry, Manipal Institute of Technology, Manipal Academy of Higher Education, 576104 Manipal, Karnataka India

**Keywords:** Thiophene, Fluorescence, Colorimetric probes, Detection, ICT, PET

## Abstract

The recognition and sensing of various analytes in aqueous and biological systems by using fluorometric or colorimetric chemosensors possessing high selectivity and sensitivity, low cost has gained enormous attention. Furthermore, thiophene derivatives possess exceptional photophysical properties compared to other heterocycles, and therefore they can be employed in chemosensors for analyte detection. In this review, we have tried to explore the design and detection mechanism of various thiophene-based probes, practical applicability, and their advanced models (design guides), which could be thoughtful for the synthesis of new thiophene-based probes. This review provides an insight into the reported chemosensors (2008-2020) for thiophene scaffold as effective emission and absorption-based chemosensors.

## Introduction

Over the years, the revolution in industrial and agricultural sectors has brought plenty of new products and services. Along with the production of useful technologies, these advancements utilize more chemicals to meet the requirements of the population. The release of such chemicals into the environment is mainly responsible for destroying the environmental and biotic systems. Many environmental pollutants such as heavy metals and other transition metals enter the environmental systems through natural and anthropogenic activities. These are detrimental to life due to their toxicity. Hence, detection of these metal ions is vital. Therefore, there is a need to develop convenient and realistic techniques for the quick detection of diverse metal ions in biological and ecological environments. The progress of selective optical signaling systems for ion detection is an emerging subject of interest in research from recent years due to the potentiality of such systems in analytical devices. Among the various available analytical tools for ion detection, fluorometric and colorimetric analysis are among the most important and most preferred optical tools that are used for the detection of various ions and molecules in a particular system due to their high selectivity, fast response, better sensitivity, real-time monitoring [1], and bioimaging capability [2, 3].

From this point of view, various chemosensors have been researched and reported for the discernment of ions. Chemosensors are molecules that show a slight change in their emission properties in response to their interaction with the binding ions of the surrounding environment [4]. Furthermore, it has been known that two important processes take place during the detection of the analyte. Firstly, being molecule recognition, and the second one being signal transduction. On this basis, chemosensors can be cataloged into a receptor that binds with the analyte of interest, an active unit that changes its properties upon binding to the analyte, and a spacer that can tune the electronic properties between the above two moieties [5].

**Fig. 1:**
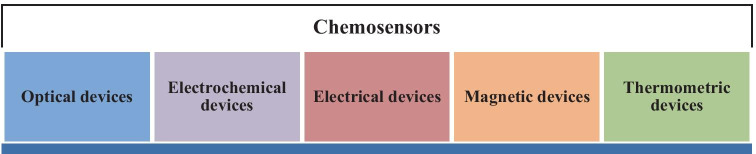
General classification of chemosensors

As a requirement for designing chemosensors to detect specific analytes, three pivotal factors need to be taken into consideration: high selectivity, high sensitivity, and high specificity [5]. Designing an effectual chemosensor possessing both selectivity and sensitivity features for analyte detection is troublesome. Chemosensors are sensitive to changes in the environment [6] and hence they can be generally categorized according to the operating principle of the transducer (Fig. 1) [7].

Optical devices are based on the changes of optical phenomena, such as absorbance, reflectance, luminescence, flourescence which is the outcome of an interaction of the analyte with the receptor of the sensor. Electrochemical devices transform the changes of the electrochemical interaction analyte - electrode into a signal which include voltammetric sensors, potentiometric sensors. Electrical devices sense the change in the electrical properties of the analyte without any electrochemical processes. Few examples are electrolytic conductivity sensors, electric permittivity sensors. Further, magnetic devices rely on the change of paramagnetic properties of a gas which is being analysed. Lastly, thermometric devices are based on the measurement of the heat effects of a specific chemical reaction or adsorption that involves the analyte.

Nevertheless, the detection of analytes or ions through chemosensors is usually attained by the selective coordination to the target analyte resulting in the change in the intensity of fluorescence of the fluorophore with the concentration change of ions [8], via photo-physical mechanisms such as chelation induced enhanced fluorescence (CHEF) [9], photoinduced electron transfer (PET) [10], intramolecular charge transfer (ICT) [11], Foster Resonance Energy Transfer (FRET) [12, 13] and various other approaches. These chemosensors provide a highly sensitive, inexpensive, reliable alternative along with simple visualization of analytes without sophisticated instruments [14].

One of the extensively used chemosensors is thiophene-based (thiophene derivatives), and they have been employed as a fluorescence signaling promoter to organic acids, metal ions, and cations [15]. Thiophene is one of the most studied five-membered heterocyclic compounds. It has been observed that five-membered heterocyclic compounds often show strong photoluminescence properties, such as long emission and absorption wavelengths, vast absorption co-efficient, and more fluorescence quantum yield, it is widely used in light-emitting materials [16] and therefore, the thiophene moiety can be employed as a functional group as a part of the chemosensor. Because of its high potential of structural variation, along with the high polarizability of sulfur atoms in the ring results in a stabilization of the conjugated chain, thereby having magnificent electronic and charge-transfer properties [17]. Oligo and polythiophenes have fluorescence frequencies that are tunable over a broad visible range and possess high absorbencies and high fluorescence efficiencies [18] and therefore, they are being employed as fluorometric and colorimetric chemosensors for the detection of proteins.

The main objective of this review is to focus light on the recent progress in the development of thiophene-based chemosensors that have been employed for the detection of cations (Al^3+^, In^3+^, Hg^2+^, Pb^2+^, Cr^3+^, Zn^2+^, etc.) and anions (CN^−^, F^−^, I^−^ etc.). The chemical and physical interactions of these sensors with the analytes resulting in changes in the emission intensity and probe color are explored. The practical applications of these probes in the real world are also summarised.

## Thiophene Based Chemosensors for Detection of Cations

Thiophene-based chemosensors provide a novel approach with an easy synthetic route and cost-effective technique for selective and quantitative detection of metal ions in biological and environmental systems. The coordination of a cation to a fluorescent chemosensor might enhance the fluorescence emission, called as Chelation Enhanced Fluorescence Effect (CHEF), or quench the fluorescence emission, called as Chelation Enhancement Quenching Effect (CHEQ). These changes in the emission intensity are employed for analyte detection.

### Aluminium

Aluminium is the most abundant non-ferrous metal in the earth’s crust. Most of the foods and antiperspirants contain aluminium compounds, and hence we are exposed to them daily. However, excessive exposure to Al^3+^ to the human body can cause awful illnesses like Parkinson’s and Alzheimer’s disease [19], threatens aquatic species by clogging the gills due to aluminium accumulation [20], and also affects plant growth and development. Therefore, there is a necessity for the development of chemosensors for the recognition of aluminium ions.

Considering the harmful effects, Jeong et al. designed a multifunctional fluorescent ‘turn-on’ chemosensor **1** using the thiophene moiety along with the diethyl aminophenol moiety (electron-donor) for the recognition of Al^3+^ in a near-perfect aqueous solution. The UV-Vis titration in bis-tris buffer (pH 7.0) revealed that, upon the addition of Al^3+^, the absorption intensity of the peak at 370 nm lessened and the absorption intensity at 420 nm increased, therefore showing high selectivity towards Al^3+^ ions. It was also found that the fluorescence intensity of the chemosensor escalated only in the presence of Al^3+^ ions. Job’s plot analysis showed that there was a 1:1 stoichiometric ratio between Al^3+^ and chemosensor, and the better selectivity of the fluorescent chemosensor towards Al^3+^ (hard acid) is attributable to the presence of two oxygen atoms (hard base). Furthermore, the binding constant of chemosensor **1** calculated from Benesi-Hildebrand was found to be 7.56 × 10^2^_,_ and the LOD (Limit of Detection) was observed to be 0.41 µM.

**Scheme 1:**
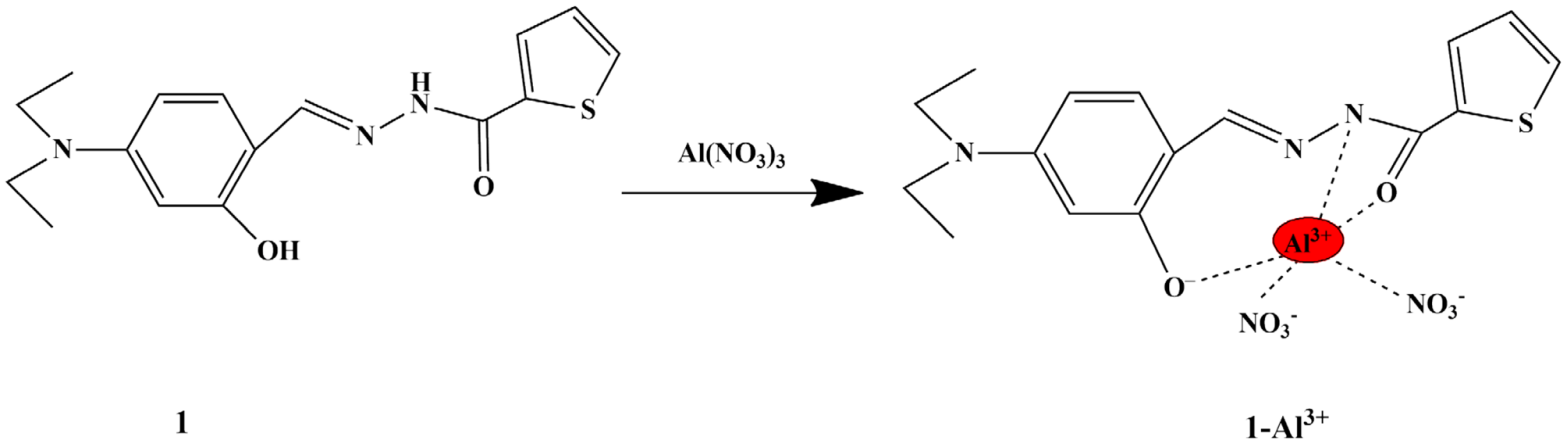
Proposed mechanism of sensor **1** with Al^3+^

Furthermore, evaluation of the feasibility of chemosensor **1** for Al^3+^ detection biological systems was carried out by in vitro cell imaging experiments using HeLa cells, and it was found that the fluorescence intensity augmented with the increase of Al^3+^ ion concentration in the cells, thereby indicating that the fluorescent chemosensor could be employed as a biocompatible detector in the determination of Al^3+^ ions in biological systems. The proposed sensing mechanism for Al^3+^ detection was ICT and deprotonation by hydrogen bonding **(**Scheme 1**)**, as supported by TD-DFT and DFT studies [15].

**Scheme 2:**
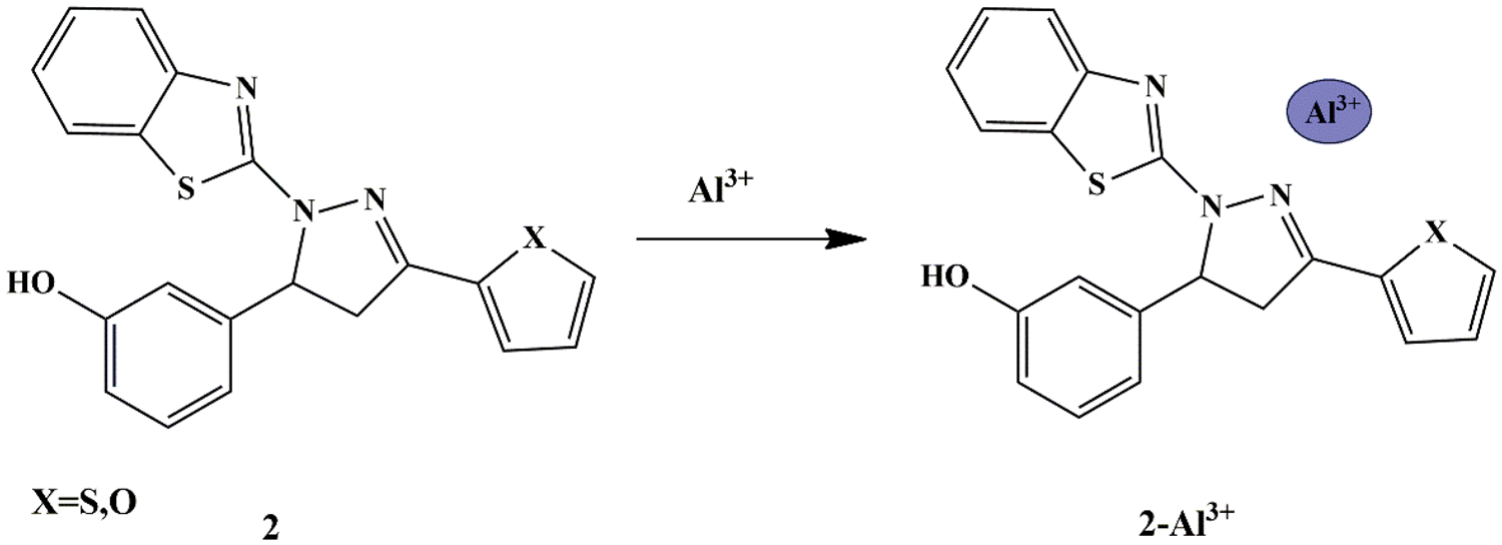
Sensing mechanism of chemosensor **2** to Al^3+^ ions

Manjunath et al. synthesized thiophene appended pyrazoline based ‘Turn-off’ fluorescent chemosensor **2** and evaluated its sensing properties in HEPES buffer solution at pH 7.2. The UV-Vis absorption band at 358 nm exhibited by the free **2** sensor shifted to 349 nm thereby exhibiting decreased intensity upon the addition of Al^3+^ ions only due to the coordination of sensor **2** with Al^3+^ ion **(**Scheme 2**)**. Furthermore, the free sensor **2** showed deep blue fluorescence emission at 447 nm, which was further quenched only in the presence of Al^3+^. The association constant for the binding of Al^3+^ ion in **2** was determined to be 1.84 × 10^4^ M^−1^ with 1:1 stoichiometry [21] and the LOD for Al^3+^ was 8.92 × 10^−8^ M, which was sufficiently lower than the permissible levels of Al^3+^ in drinking water as set up by WHO, thereby showing potential to examine drinking water quality.

### Indium

Indium is used in biological and diagnostic disciplines, such as in treating cancer or other radiopharmaceuticals, owing to its less toxicity. But compounds of Indium, namely Indium phosphate (IP) and Indium tin oxide (ITO), have been recognized to have potential risk for respiratory diseases. Hence, detecting indium selectively is in high demand. Significantly, few fluorescent chemosensors have been developed to detect indium since inhibition from Al^3+^ and Ga^3+^ (belonging to the same group of metal ions) occurs. Thus, there is a need to develop selective chemosensors by fluorescent ‘turn on’ method for differentiating In^3+^ from Al^3+^ and Ga^3+^ ions.

**Scheme 3:**
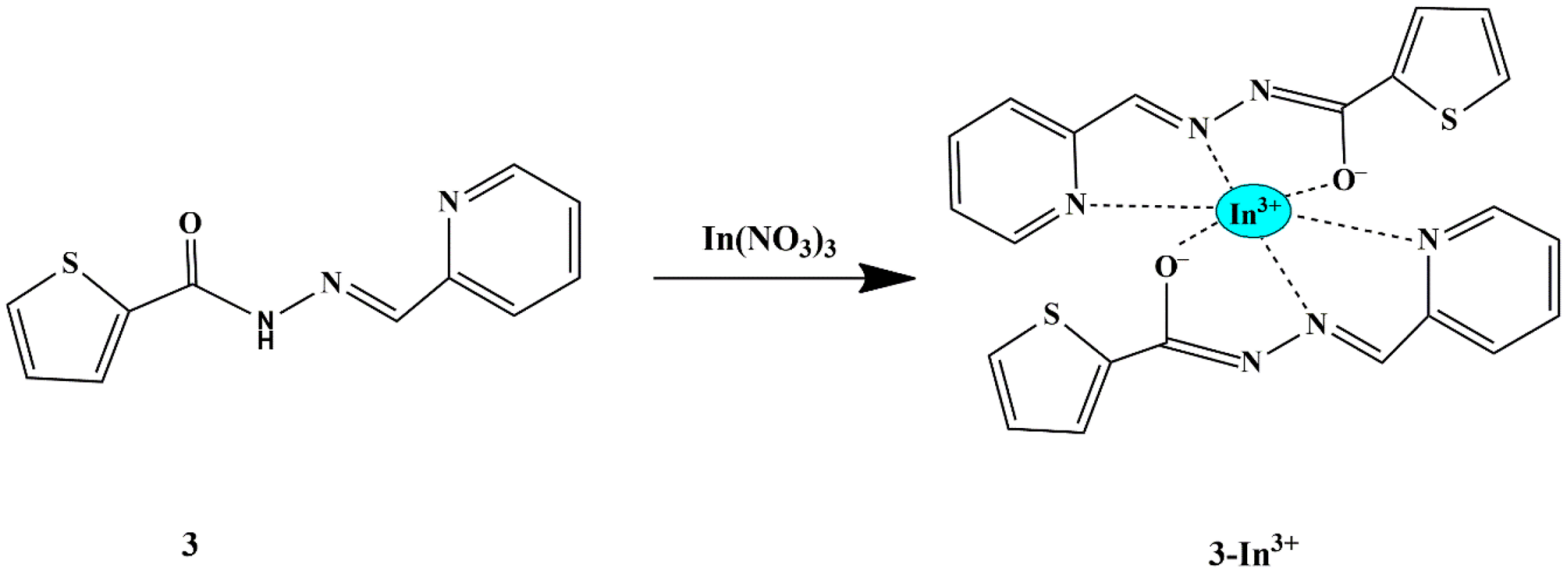
Sensing mechanism of chemosensor **3** towards In^3+^

In this regard, Hanna Cho designed the synthesis of ‘Turn-on’ chemosensor **3** [(Z)-N′-(pyridine-2-ylmethylene)thiophene-2-carbohydrazide] by integrating thiophene-2-carbohydrazide with picolinaldehyde. When Cd^2+^, Al^3+^, Cu^2+^, etc., were added to the chemosensor TP in EtOH, there was no significant fluorescent response, except for In^3+^, which showed a fluorescence emission at 460 nm [22] due to the CHEF effect. The distinctive selectivity of probe **3** towards In^3+^ is assigned to the size effect and the hard-soft principle, indicating that it could effectively distinguish In^3+^ from Al^3+^ and Ga^3+^ because of their alike properties [23] **(**Scheme 3**)**. UV-Vis titration showed that the absorption band (exhibited by the free sensor) gradually decreased at 310 nm and a new band at 379 nm emerged due to the presence of In^3+^. The association constant for the 2. (**3)**−In^3+^ complex was determined by Li’s equation to be 8.1 × 10^4^ M^−2^. The LOD was estimated to be 0.61 µM, which was sufficiently lowest amid all the formerly reported chemosensors for In^3+^ detection.

### Mercury

For decades, heavy metal pollution of the environment has been a significant issue due to its threat to biotic life [24, 25]. One of the most hazardous and commonly occurring heavy metal [26] pollution in the aqueous system is mercury pollution since Hg^2+^ ions can remain persistent in the aqueous environment and even a minute amount of Hg^2+^ ions cause health issues such as respiratory damage, gastrointestinal problems, Minamata disease, and prenatal brain damage. Mercury ion is spectroscopically and magnetically silent (d^10^) [27] and therefore cannot be rapidly detected by the available detection techniques such as NMR and EPR. Thus, there is an essential need for new chemosensors to detect Hg^2+^ ions in the aqueous system.

**Scheme 4:**
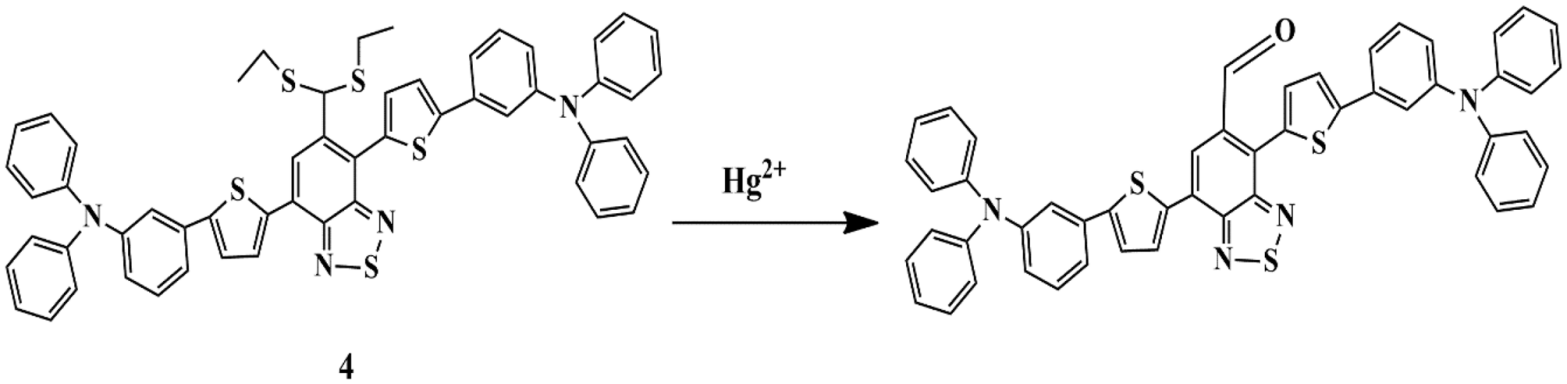
Sensing mechanism of chemosensor **4** with Hg^2+^

In this regard, Zhang et al. developed a thiophene-based ratiometric fluorescent chemosensor **4** possessing triphenylamine and benzothiadiazole moieties, since thiophene can outstretch the π conjugation and also stabilize the quinoid structure and contribute in tuning the energy gap of the D − π − A − π − D type of chromophore, and the presence of the π bridge of thiophene could increase the fluorescence resonance wavelength up to 80 nm. UV-Vis absorbance spectra of probe **4** revealed that only Hg^2+^ promoted a redshift from 489 nm to 535 nm, accompanied by red to violet color change. Probe **4** exhibited a drop in the fluorescent intensity band centered at 656 nm and an increase in the band centered at 723 nm [28], only in the presence of the Hg^2+^ without interference from other competing metal ions. The LOD was determined to be 0.36 µM for Hg^2+^ ions. Furthermore, the sensing mechanism was found to be due to the Mercury-promoted desulfurization reaction **(**Scheme 4**)**.

**Scheme 5:**
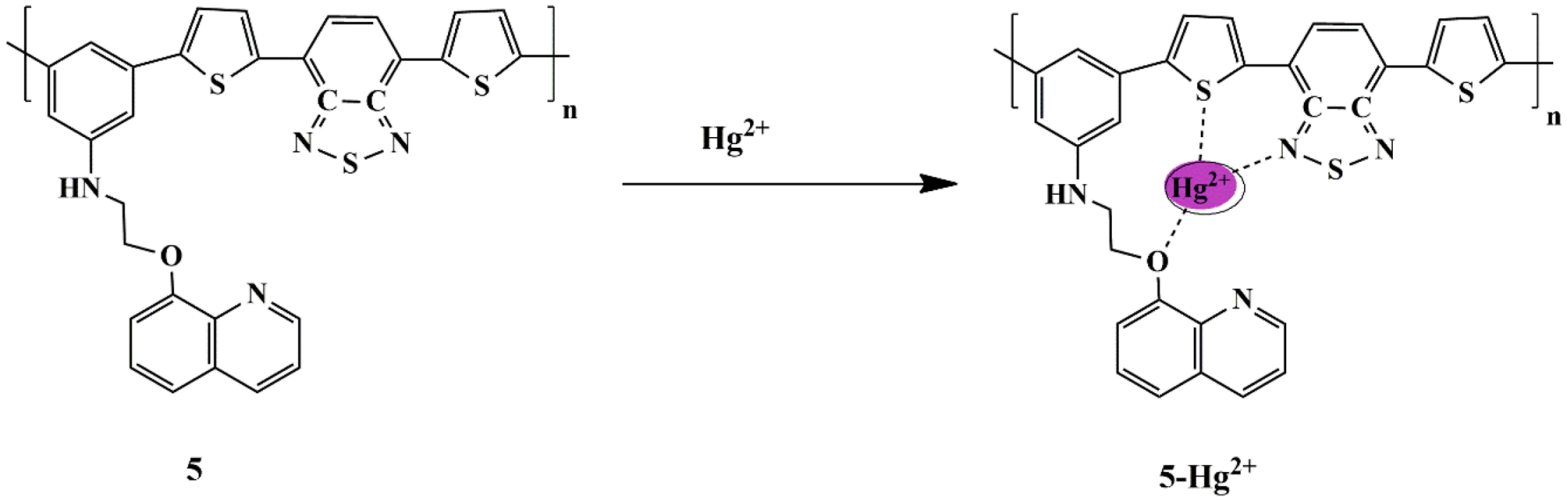
Plausible sensing mechanism of probe **5** towards Hg^2+^

Over the last few years, the π-conjugated polymer has gained increased attention due to its excellent optical properties and signal amplification because of repeating units. Based on these properties, Liheng et al. synthesized a red-emitting ‘Turn-on’ polymer fluorescent probe **5** occupying thiophene, benzothiazole, and quinoline groups for specific sensing of Hg^2+^ ions and evaluated its potential in practical applications (in cosmetics). It was noticeable that only Hg^2+^ ions could enhance the fluorescence intensity of the probe in DMSO, which was evident by the color change from blue to red (under UV light) and could be explained based on the PET process [29] **(**Scheme 5**)**. The binding of probe **5** and Hg^2+^ was found to be in a 1:1 stoichiometric ratio, and the response time was quick, being less than 10 s. It was observed that similar fluorescence changes of probe **5** could also be observed with the increase of Hg^2+^ ions in cosmetics, thereby making it a valuable tool for the monitoring of Hg^2+^ in practical applications.

**Scheme 6:**
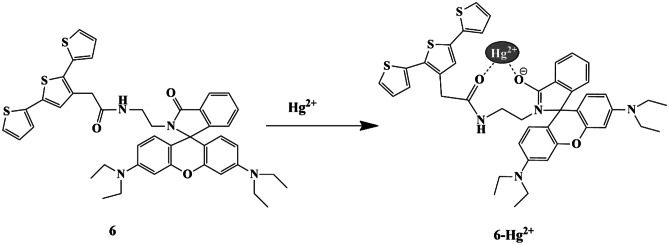
A suggested mechanism for sensor **6** towards Hg^2+^

Nanostructured materials possess unique physical and chemical properties and possess potential applications in catalysis, sensors, and magnetic devices. Considering these into account, Chatteikaewtong et al. synthesized a terthiophene functionalized rhodamine fluorescence resonance energy transfer (FRET)-based chemosensor **6** and probe **6**-gold nanocomposite based-chemosensor **7** for Hg^2+^ recognition. It was observed that the fluorescence spectra and UV-Vis spectra of probe **6** exhibited a high-fluorescence band at 578 nm and an absorbance intensity of 555 nm, respectively, upon the addition of Hg^2+^ ions only, owing to the Hg^2+^ induced ring-opening of the spirolactam Rhodamine form **(**Scheme 6**)**. The absorption spectra of probe **7** exhibited a significant redshift, with the color change from colorless to black, and were credibly due to the aggregation of gold nanoparticles by Hg^2+^ chelation, and was evident by TEM images. A 2:1 binding ratio for the probe **6**−Hg^2+^ and probe **7**−Hg^2+^ complex was concluded, and the LOD of probe **7** was calculated to be 0.32 µM which was relatively lower [30] compared to that of probe **6** (LOD=1.34 µM), and the detection time was less than 30 s, thereby exhibiting a high sensitivity towards Hg^2+^ ions.

**Scheme 7:**
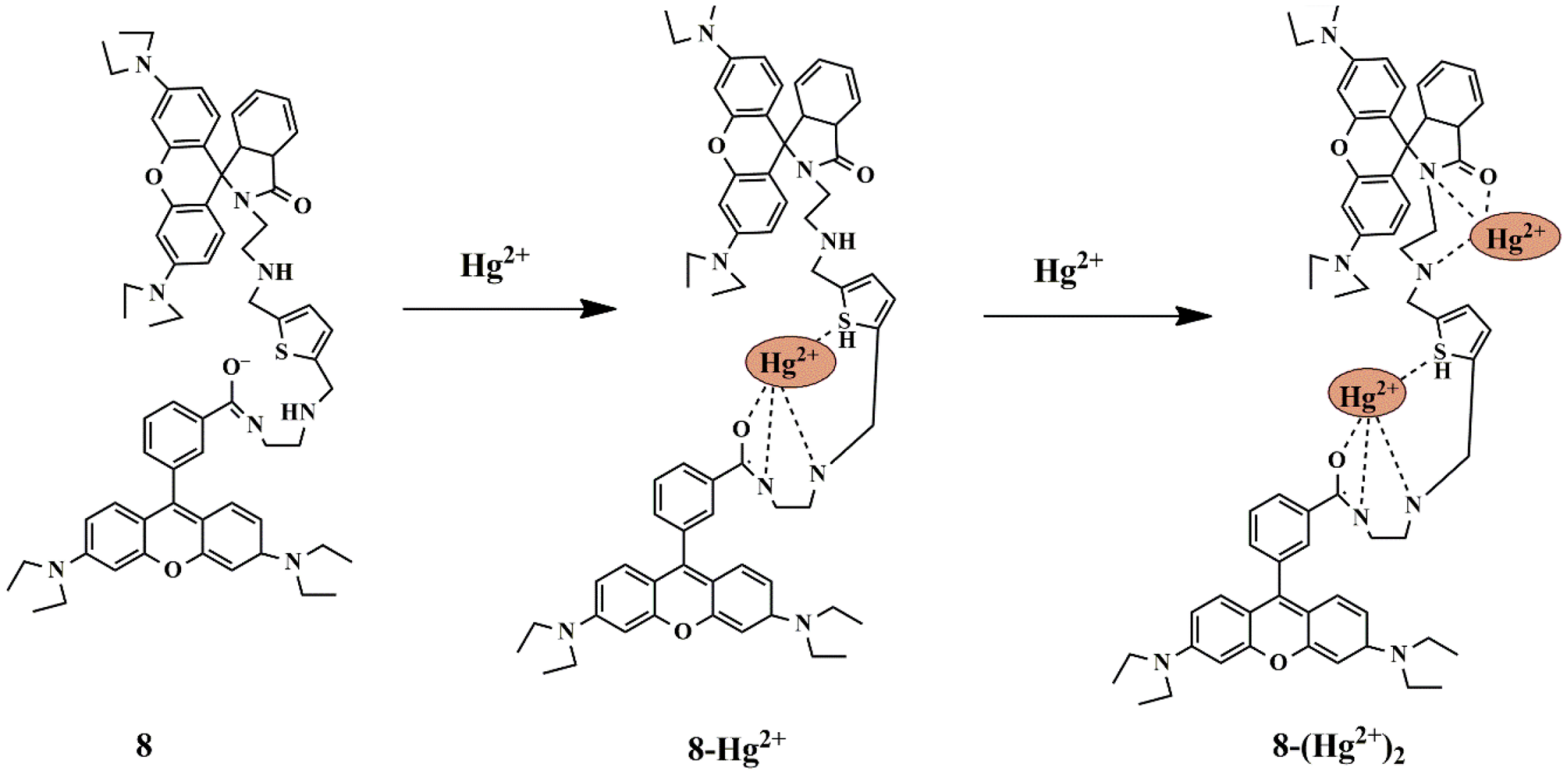
Sequential complexation of Hg^2+^ ions to sensor **8**

Considering the photophysical properties of thiophene and rhodamine, Ajoy Pal et al. synthesized a thiophene derivatized rhodamine chemosensor **8** [thiophene-2,5-di-(methyl-amino-ethyl rhodamine)] for Hg^2+^ detection. The sensor in CH_3_CN exhibited a maximum absorption peak at 557 nm and an ensuing color change to pink in the presence of Hg^2+^ only. Further, UV-Vis spectral studies and fluorescence studies were carried out in CH_3_CN/H_2_O mixture to confirm the sensing property of sensor **8** towards Hg^2+^, and it was evident that only Hg^2+^ ions imparted an immediate color change from colorless to pink with maximum fluorescence. However, the other tested metal ions exhibited negligible fluorescence enhancement. The absorbance and fluorescence changes could be assigned to the metal ion-induced spiro-ring opening of rhodamine moiety. The LOD and metal ion/ligand binding stoichiometry was 2.2 × 10^−8^ M and 1:2 respectively. The interaction between sensor **8** and Hg^2+^ was evidenced from ESI-MS analysis, which suggested that sensor **8** selectively binds to the first Hg^2+^ ion via one of its ‘amino-ethyl-amido’ units along with its central sulphur atom, which then promotes a spatial reorientation of the ligand and results in a faster binding of the second Hg^2+^ ion **(**Scheme 7**)**. To examine its reusability, CO_3_^2−^/HCO_3_^−^ and CH_3_COO^−^ have been found to induce the reversibility in sensor **8**−Hg^2+^ complexation and the structural transformation of Rhodamine [31], thereby rendering a reversible dual-channel signaling pattern.

**Scheme 8:**
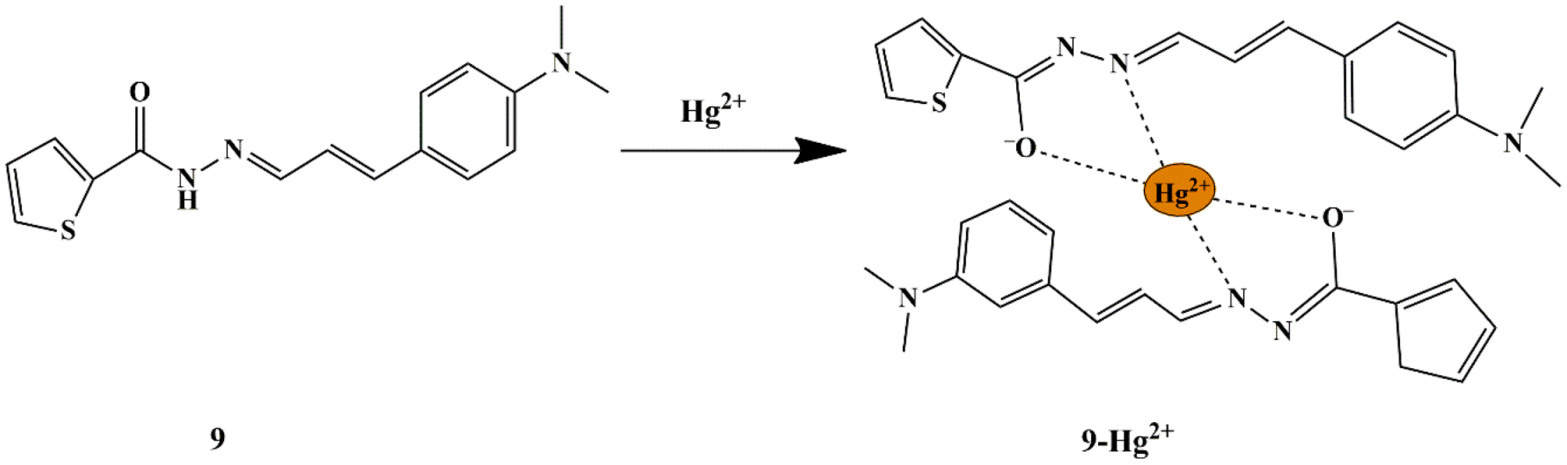
Possible sensing mechanism of sensor **9** towards Hg^2+^

Hanna et al. synthesized cinnamaldehyde-based colorimetric chemosensor **9** and the thiophene derivative (with hydrazide) was introduced to overcome the drawback of the less solubility of cinnamaldehyde in water. It was observed that the absorption band of 390 nm, exhibited by free sensor **9** in bis-tris buffer had a noticeable decrease, and a new broad absorption band of 518 nm emerged with the color change from pale yellow to orange, only upon the inclusion of Hg^2+^ ions due to the probe **9**-Hg^2+^ complex. The spectral response of **9** was noted in a broad pH range of 6-9. The binding constant was determined to be 2 × 10^9^ M^−2^ with 2:1 stoichiometry. The LOD was calculated to be 0.01 µM for Hg^2+^ and the sensing properties could be attributed to the ICT mechanism **(**Scheme 8**)**, supported by DFT calculations [32].

**Scheme 9:**
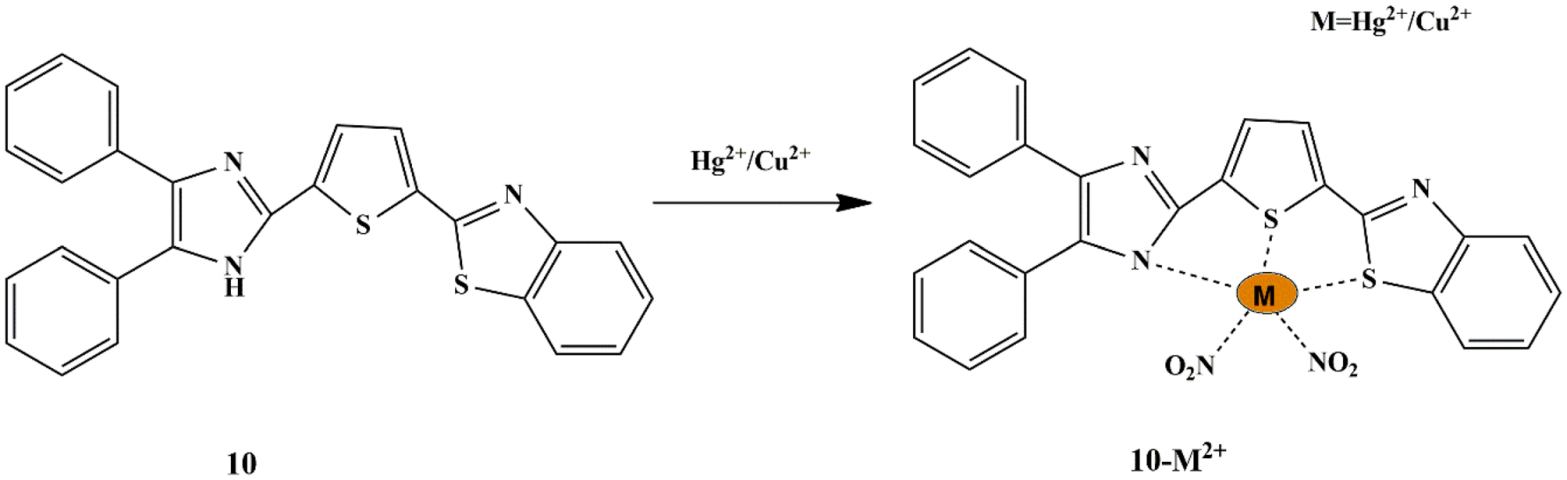
Plausible sensing mechanism of probe **10** towards Hg^2+^/Cu^2+^

Recently, plenty of chemosensors have been designed to separately detect Hg^2+^ and Cu^2+^ ions, since Cu^2+^ ion interferes with the detection of Hg^2+^ ions. Influenced by the difficulties in the selective sensing of Hg^2+^ and Cu^2+^ ions, Syed et al. developed a thiophene-based probe **10** for multiple metal-ion recognition, past distinct output signals. The naked-eye experiment revealed that the fluorescent green color of the free probe in HEPES buffer switched to blue with Hg^2+^ and non-fluorescent with Cu^2+^ ions respectively under UV light. The absorption spectra of the free probe **10** displayed a low energy charge transfer at 395 nm and a high energy band at 328 nm, which raised and shrunk only with Hg^2+^ and Cu^2+^ ions, respectively. Moreover, upon interaction with Hg^2+^, a negligible drop in the relative fluorescence intensity was spotted and upon interaction with Cu^2+^, significant fluorescence quenching was notable, which could be attributed to the CHEF and CHEQ mechanisms, respectively **(**Scheme 9**)**. By the results, it is noticeable that the fluorescence intensity of the probe **10**-Hg^2+^ complex was uninfluenced upon interaction with Cu^2+^ ions. Likewise, the quenched fluorescence emission of the probe **10**-Cu^2+^ complex does not get enhanced upon interaction with Hg^2+^, thereby providing a means to detect both ions with higher selectivity and distinct output emission signals. Furthermore, the reversibility studies were performed using EDTA, which suggested a reversible complexation mode between probe **10** and metal ions. The binding constants were 3.1 × 10^5^ for Hg^2+^ and 1.6 × 10^5^ for Cu^2+^, and the binding of the probe to Hg^2+^/Cu^2+^ was found to be in a 1:1 stoichiometric ratio. Furthermore, the LOD to detect Hg^2+^ and Cu^2+^ ions were ascertained to be 28 µM and 7.5 µM respectively [33]. Probe **10** has been reported to have good cell permeability to detect Hg^2+^ and Cu^2+^ in live cells and protein medium, thereby proving its potential to discern metal ions in the biological system. Besides, a molecular logic gate was also constructed and the probe has been explored as a coding/decoding fluorescent blue ink.

**Scheme 10:**
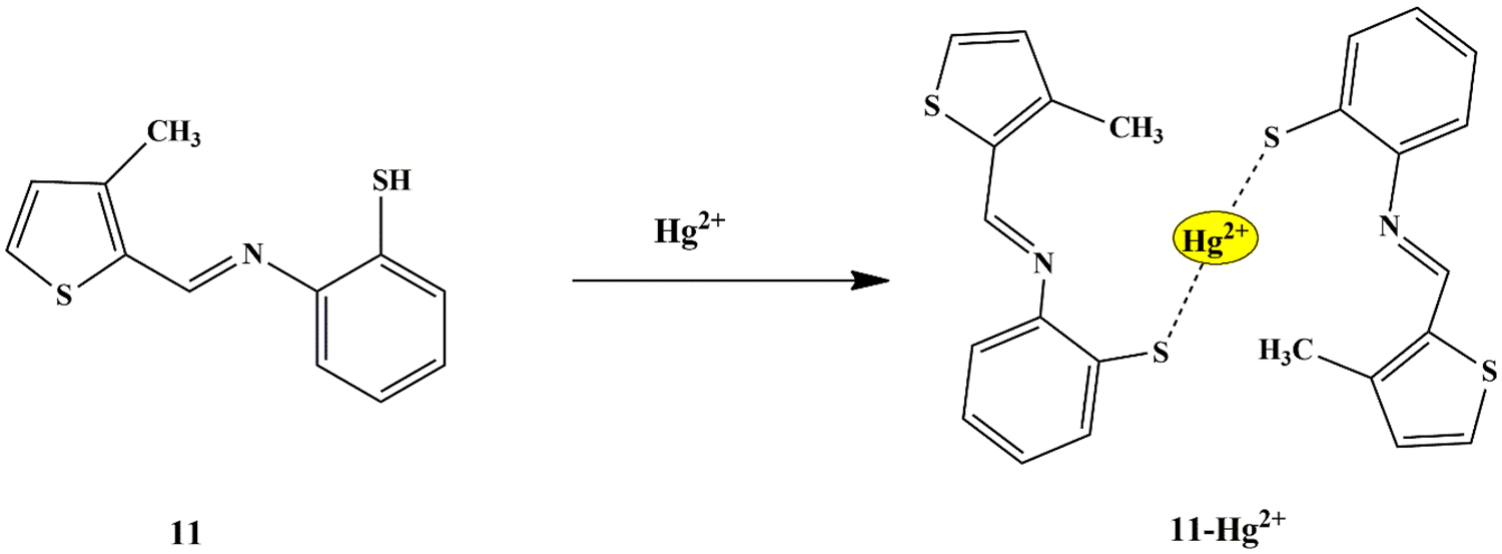
Possible mode of binding interaction of probe **11** with Hg^2+^

Divya Singhal synthesized a chromogenic and fluorogenic thiophene-based schiff base probe **11** [2-((3-methylthiophen-2-yl)methyleneamino)benzenethiol], for selective recognition of Hg^2+^ ions. It was seen that the probe showed an immediate color change from light yellow to yellowish-orange only for Hg^2+^ ions. Furthermore, UV-Vis absorbance and fluorescence studies were investigated in CH_3_OH/H_2_O (v/v 8:2), which revealed that the addition of Hg^2+^ displayed a new band at 430 nm in addition to 287 nm and 355 nm absorption bands of the probe, due to the ICT mechanism. The turn-on fluorescence emission, exhibited by probe **11** in response to Hg^2+^ ions was seen at 503 nm, which was due to the CHEF mechanism [34] and the probe was free from interference from other tested ions, thereby clearly indicating the selective sensing of Hg^2+^ ions and the binding interaction was further confirmed by NMR, Mass spectra, DFT studies, and electrochemical behavior **(**Scheme 10**)**. The reversibility studies of the sensing mechanism were performed using EDTA, which showed a fluorescence quenching and disappearance of the color of probe **11**, in contrast to that of probe **11**−Hg^2+^ sensing mechanism. A 2:1 stoichiometry for probe **11** and Hg^2+^ and a detection limit of 20 µM was noted.

### Lead

Lead is one of the noxious heavy metals, and its accumulation can result in lead poisoning, consequently leading to retarded brain development and nervous system disorders [35], convulsions, muscle paralysis [36], coma, and death. Very few fluorescent chemosensors have been reported for detecting Pb(II) due to their undesirable property of quenching the fluorescence emission through electron transfer [37] or spin-orbital coupling [38], which is highly disadvantageous for analytical purposes.

**Scheme 11:**
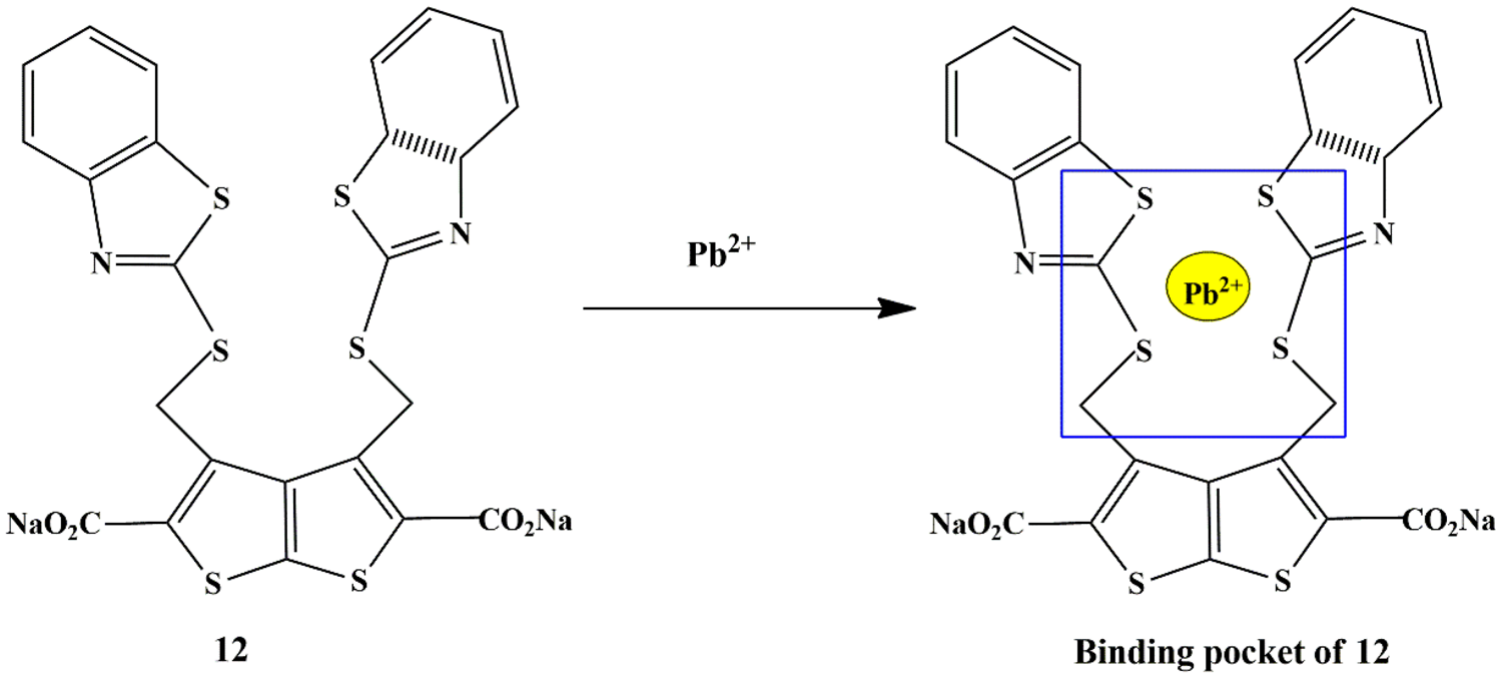
Binding pocket of probe **12** for Pb^2+^

Jing Cao et al. synthesized a novel water-soluble thiophene functionalized chemosensor **12** bearing benzo[d]-thiazole-2-thio unit for Pb^2+^ detection. It was observed that in the HEPES buffer, the probe exhibited an exceptional fluorescence intensity accompanied by a bathochromic shift in the absorption maxima, only in the presence of Pb(II), without any interference from other ions. This could be attributed to the binding pocket **(**Scheme 11**)** formed within probe **12**, which could be a probable binding site for Pb(II) [39], having a 1:1 binding stoichiometry ratio.

### Chromium

Chromium is one of the heavy metals and is widely used in metallurgy and pigments. It plays a role in regulating blood glucose levels [40] and the metabolism of proteins, carbohydrates, and fats in the body. However, chromium is carcinogenic, and its toxicity can result in Haemolysis and kidney failure.

**Scheme 12:**
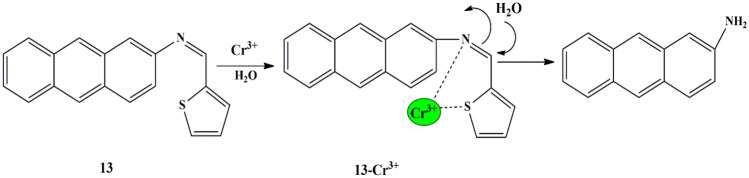
Proposed detection mechanism of probe **13** for Cr^3+^

Considering the above issues, Erman synthesized an anthracene appended-thiophene fluorescent probe **13** which possessed a low detection limit and a faster response time for Cr^3+^. It was observed that the free probe in CH_3_CN/H_2_O (v/v 6:4) had diminished fluorescence because of the PET process. However, a new strong emission peak appeared at 500 nm upon the addition of Cr^3+^. The LOD of the probe was calculated to be 0.4 µM, and it had a response time of less than 1 min. Reversibility studies were carried out by employing an excess of EDTA and it was observed that the sensing mechanism was irreversible. Moreover, the sensing mechanism is attributable to the concurrent coordination of Cr^3+^ ions with N atom on C=N moiety and S atom in thiophene, which induced the attack of water resulting in the formation of 2-aminoanthracene. To investigate the potential of probe **13** in practical applications, probe (10 µM) in CH_3_CN/HEPES solution (v/v 6:4) at pH 7 was introduced onto circular test papers. They did not exhibit emission at 366 nm under UV light in the absence of metal ions, while only Cr^3+^ ions exhibited a green emission [41] by a “turn-on” response **(**Scheme 12**)**, thereby making it an efficient tool for Cr^3+^ detection.

Conjugated polymers have acquired increased attention, owing to their high sensitivity and ease of measurement and, are therefore used as fluorescent sensors. Previously developed chemosensors have certain limitations, such as low water solubility and low sensitivity. To overcome this drawback, water-soluble π–π conjugated polymers could be employed to produce fluorescence, which is more remarkable compared to that of classic fluorophores. Considering these properties, Marco Laurenti et al. synthesized a water-soluble polythiophene-based fluorescent chemosensor probe **14** [Poly(3-ethoxythiophene-2,5-diyldimercaptosuccinic acid)] possessing chelating properties for the recognition of Pb^2+^ and Hg^2+^. The recognition properties of the probe were evaluated by UV-Vis and photoluminescence spectra of an aqueous solution of the probe. Initially, probe **14** exhibits an absorption maximum at 405 nm and 530 nm in the absorption and emission spectra, respectively. However, upon the addition of Pb^2+^ and Hg^2+^, fluorescence quenching was noticed. This could be accredited to the conformational change in probe **14**, due to the complex formed between the metal ions and the chelating DMSA moiety of the polymer [42].

**Scheme 13:**
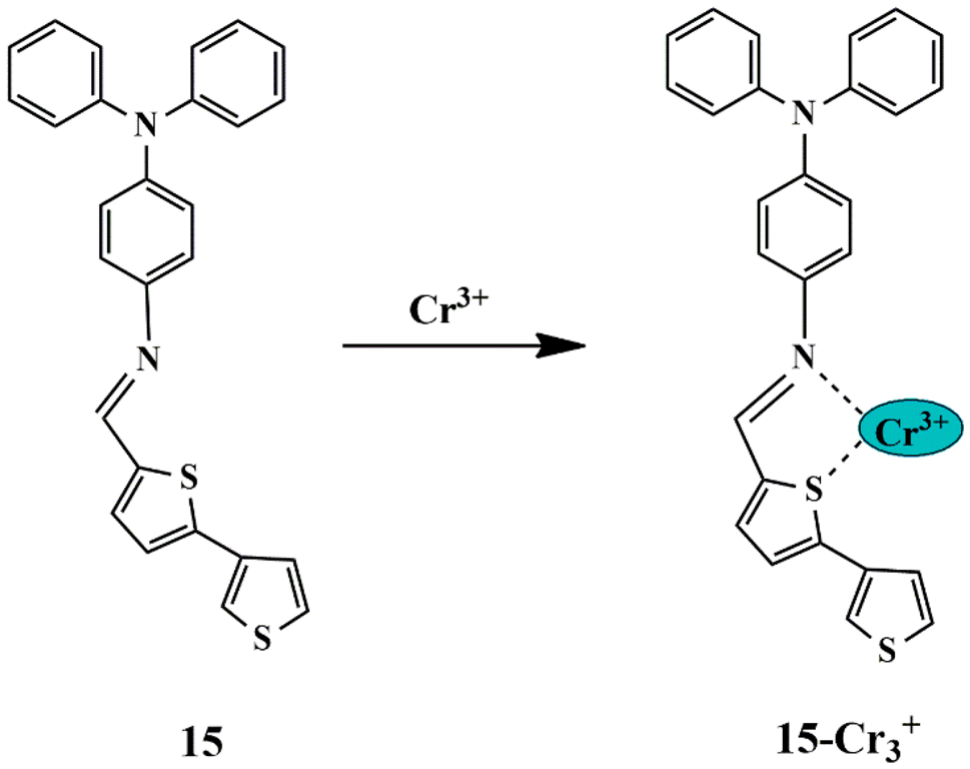
Proposed sensing mechanism of probe **15** to Cr^3+^

It has been known that schiff bases easily isomerize in the excited state but incline to display weak fluorescence. To overcome this drawback, thiophene moieties can be incorporated since thiophene exhibits significant optoelectronic properties. One such approach was made by Feyza et al. who designed a thiophene-based schiff base sensor **15** for Cr^3+^ detection ions in an aqueous solution. The probe displayed an enhancement in fluorescence intensity with a color change from yellow to Saxon blue upon binding to Cr^3+^, which is accredited to the chelation between Cr^3+^, S, and N atoms **(**Scheme 13**)**, thereby impeding the PET process. Besides that, the pH effect showed that there was enhanced fluorescence intensity upon increasing the pH, which attained a maximum (at pH 8) and then decreased, since Cr(OH)_3_ formation prevented further probe **15**-Cr^3+^ complex formation. Fluorescence studies showed that the fluorescence intensity of the probe improved with the increase in the amount of Cr^3+^ (λ_excitation_ = 320 nm) and Job’s plot analysis revealed that probe **15** and Cr^3+^ showed 2:1 binding stoichiometry. The association constant was calculated to be 2.8 × 10^4^ M^−1^ and the LOD [43] was found to be 1.5 × 10^−6^ M, and th optical behaviour of the probe was beneficial for Cr^3+^ detection in waste water.

**Scheme 14:**
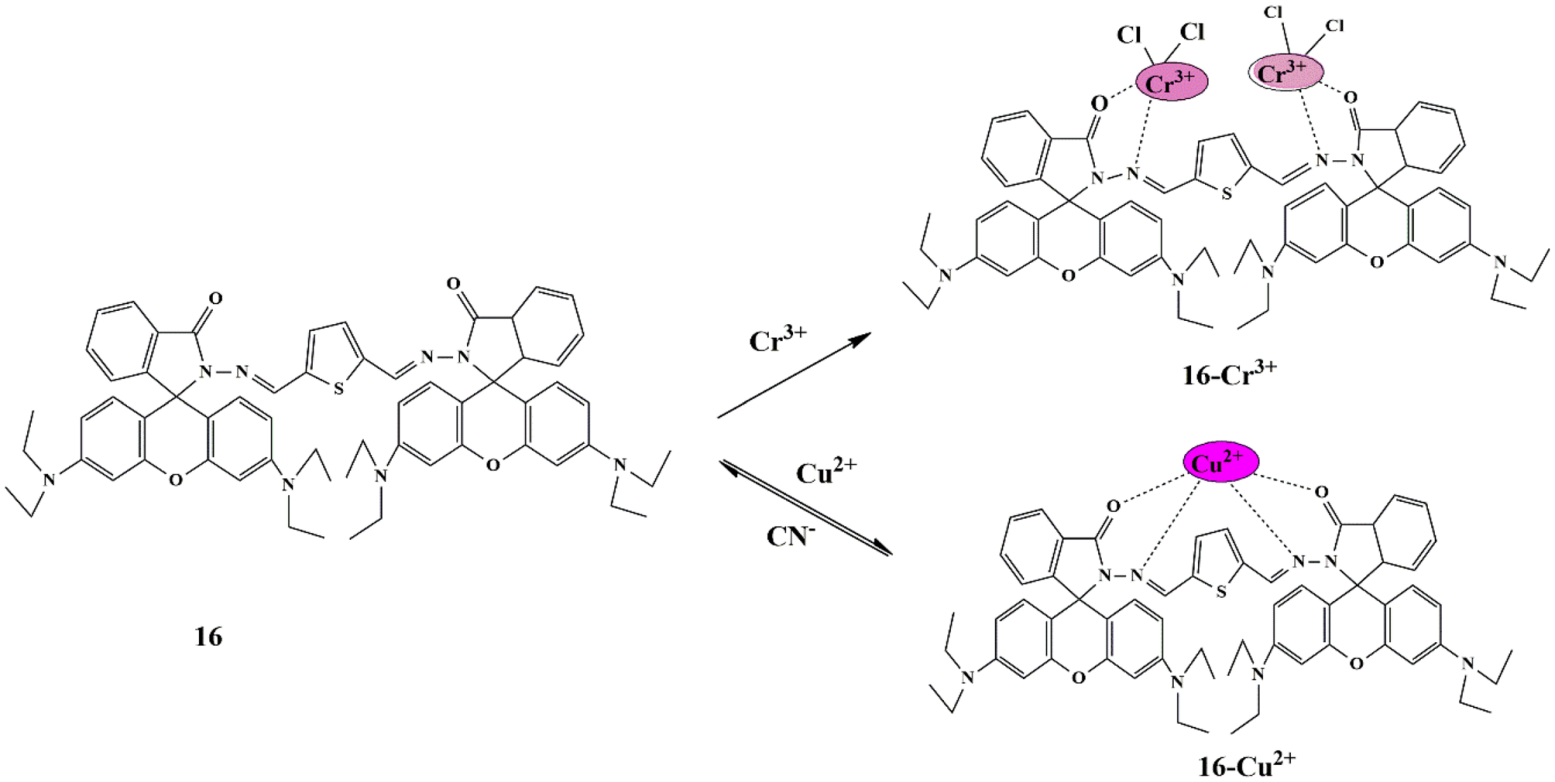
Proposed sensing mechanism of probe **16** for Cr^3+^ and Cu^2+^

Recently, a handful of chemosensors have been delineated for the simultaneous detection of both Cr^3+^ and Cu^2+^ ions. Hence, a novel approach was made by Natarajan et al. who synthesized a triple-action probe **16** [thiophenedicarboxaldehyde-rhodamine-B]. Probe in CH_3_CN/H_2_O (v/v 4:1) displayed a significant color change from light yellow to pink with Cr^3+^ and Cu^2+^ ions, while the remaining ions did not exhibit any color change, indicating that probe **16** was insensitive towards anions. But only CN^−^ ions displayed a color change when introduced to the probe **16**−Cu^2+^ solution, and a cascade recognition study confirmed the detection. The sensing property of probe **16** towards Cu^2+^, Cr^3+^, and CN^−^ could be due to the spirolactam ring-opening and spirolactam ring-closing of the rhodamine moiety, respectively **(**Scheme 14**)**. However, it was concluded that Cr^3+^ shows excellent enhancement in fluorescence intensity of probe **16** with 1:2 stoichiometry, due to the spirolactam ring opening [44] of the probe, whereas Cu^2+^ with 1:1 stoichiometry shows fluorescence quenching due to its paramagnetic nature. Moreover, the detection limits for Cr^3+^, Cu^2+^, and CN^−^ ions were 2.6 × 10^−6^ M, 1.01 × 10^−6^ M, and 9.3 × 10^−7^ M respectively. The reversibility of the probe was performed by introducing a CN^−^ into the probe **16**−Cu^2+^ complex, and good reversibility of Cu^2+^ detection sensing was observed.

### Zinc

Zinc is the third most abundant transition metal found in specific tissues of the body [45, 46]. It is responsible for the catalytic activity of few enzymes as well [47, 48]. But, excess zinc can lead to its toxicity in the body resulting in the damage of the pancreas and metabolism disturbance, consequently leading to neurodegenerative disorders and arteriosclerosis.

Several chemosensors have been reported to detect Zn^2+^ ions, such as 6-methoxy-8-quinolyl-para-toluenesulfonamide (TSQ), Zinquin, but they have limited activity because of their poor selectivity for Zn^2+^ ions. Motivated by the demand for low-cost and simple molecules for the selective recognition of Zn^2+^ ions, Dulal musib and his colleagues synthesized a novel ‘turn-on’ thiophene-based fluorescent probe **17** (Schiff-base of 2-aminophenol, thieno[2,3-b]thiophene-2,5-7,5 dicarbaldehyde). The probe showed enhanced fluorescence intensity in the presence of Zn^2+^, due to the aggregation-induced emission of probe **17** and Zn^2+^, whose luminescence was reduced by EDTA. Based on the outcomes, a logic gate was formulated for the selective detection of Zn^2+^ ions.

**Scheme 15:**
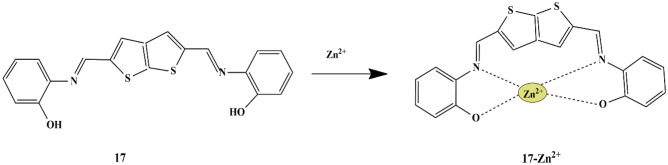
Detection mechanism of the fluorescent probe **17** towards Zn^2+^

Luminescence behavior in CH_3_CN at room temperature revealed that only Zn^2+^ ions exhibited changes in the luminescent properties of the probe, which suggested the Metal-to-Ligand charge transfer (MLCT) along with Aggregation induced emission (AIEE) [49, 50] **(**Scheme 15**)**. The AIEE property could be visualized by the appearance of deep yellow from colorless with the increase in H_2_O concentration. It was observed that the addition of EDTA results in a ‘turn-off’ fluorescence by the formation of a strong Zn^2+^−EDTA complex [8]. Furthermore, fluorescent titrations showed that, upon the addition of Zn^2+^ ions, the fluorescence emission band of probe **17** exhibited a redshift of 27 nm and a binding ratio of 1:1 was proposed.

**Scheme 16:**
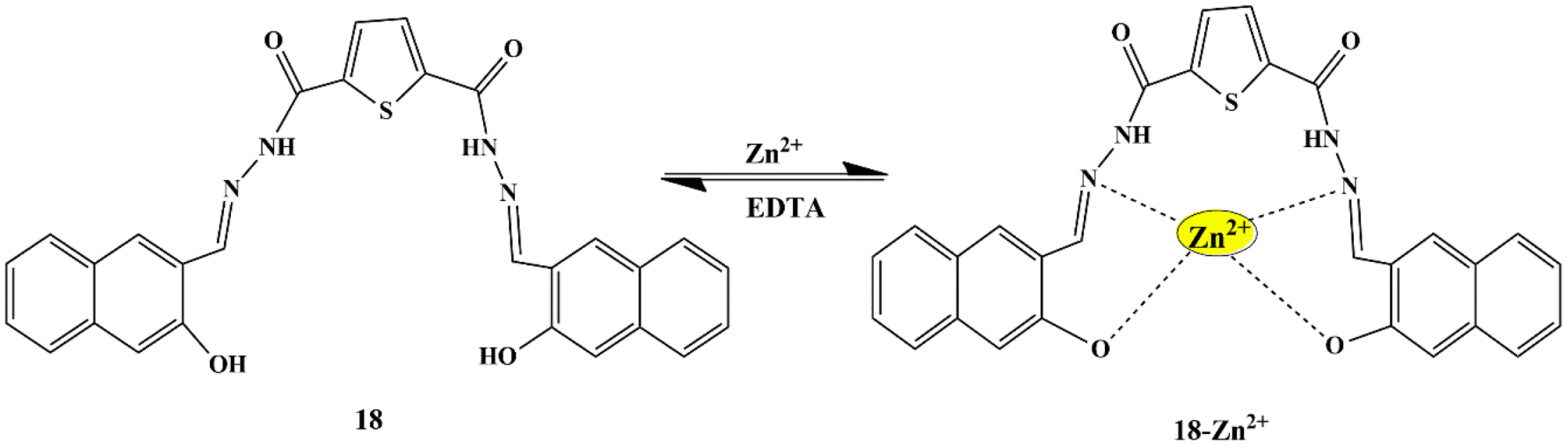
Possible mechanism of sensing of Zn^2+^ by probe **18**

Merlin Mary and Anandaram designed thiophene-appended carbohydrazide probe **18** as a ‘turn-on’ chemosensor for the detection of Zn^2+^ ions. The free probe displayed two distinct absorption peaks at 330 nm and 412 nm in the UV-Vis spectra due to Intra ligand charge transition (ILCT) [51, 52]. While, upon the addition of Zn^2+^ to the probe in the DMSO/H_2_O mixture (v/v 6:4), a new peak at 445 nm was observed, which proved probe **18**−Zn^2+^ complex formation **(**Scheme 16**)** and was further confirmed by FT-IR studies. Absorption spectral titration experiments revealed that the redshift could be observed for the probe **18**-Zn^2+^ complex with 1:1 binding stoichiometry and the LOD was found to be 1.51 × 10^−7^ M. Moreover, significant color changes (light yellow to fluorescent yellow) were observed with fluorescence emission occurring at 1890 nm, due to the coordination bond formed between probe **18** and Zn^2+^, and the fluorescence enhancement could be attributed to the CHEF effect of the complex formed [53].

**Scheme 17:**
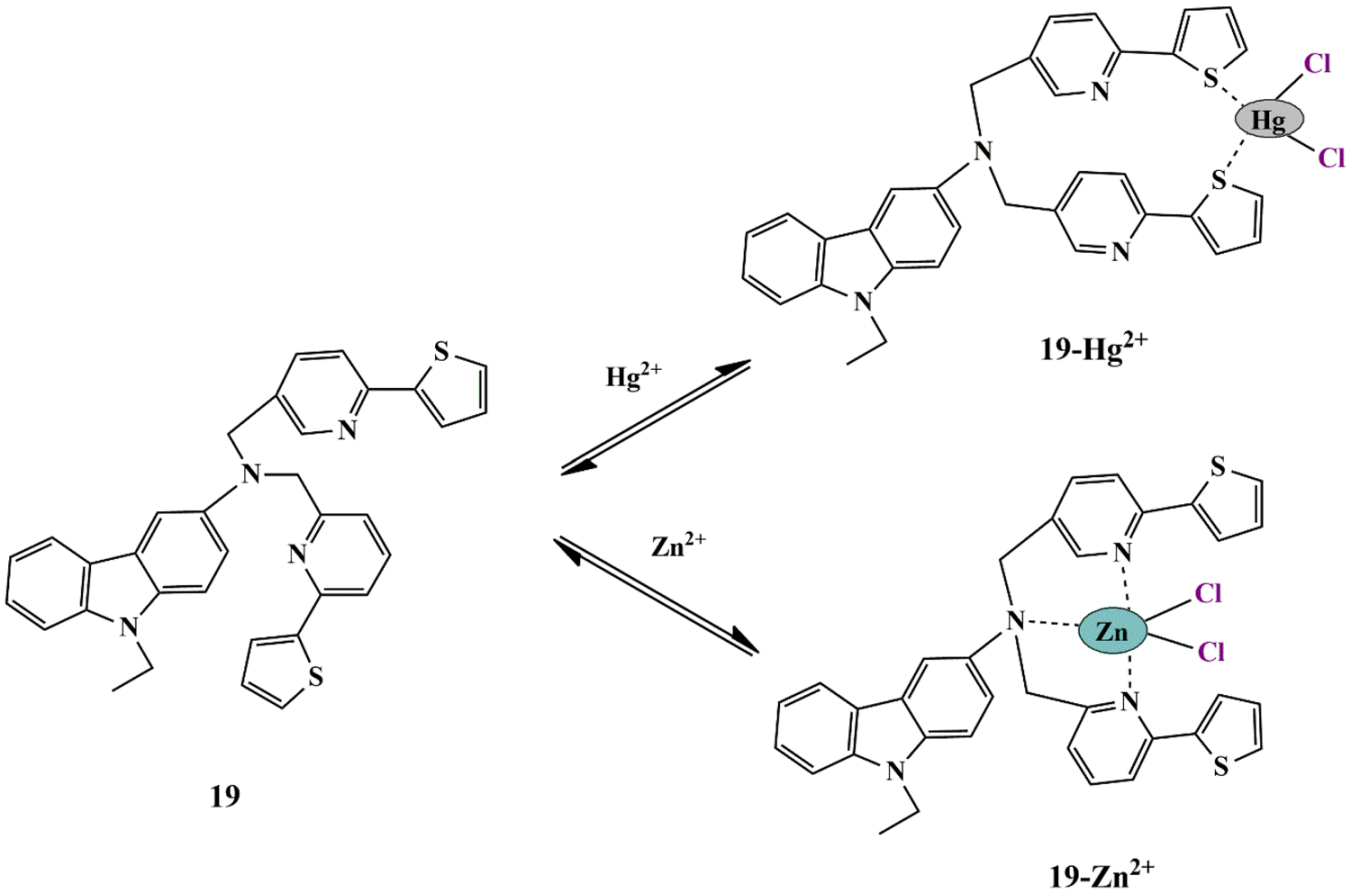
Possible mechanism of sensing of Hg^2+^ / Zn^2+^ by probe **19**

Although many probes have been synthesized for Zn^2+^ and Hg^2+^ ion recognition, thiophene-carbazole-based probes are utilized rarely. Hence, as a new approach, Ajit et al. incorporated thiophene moieties into the carbazole-based ratiometric probe **19**. Distinct UV-Vis spectral behaviors were observed upon sensing of Zn^2+^ and Hg^2+^ in HEPES buffer. The probe displayed a weak band at 385 nm upon the addition of Zn^2+^ ions, whereas it exhibited new strong redshift absorption bands followed by a naked eye visible color change from colorless to pale yellow upon the introduction of Hg^2+^ ions. Job’s plot indicated the 1:1 stoichiometry of probe **19**−Zn^2+^/Hg^2+^ complex **(**Scheme 17**)**, and Zn^2+^ binds to probe **19** by at least 1.17-fold stronger than that of Hg^2+^ as confirmed by emission studies [54]. For increasing concentrations of Zn^2+^ and Hg^2+^, the band at 418 nm gradually decreased and a new band at 520 nm is seen only for Hg^2+^ along with a visual fluorescent color change. At the same time, the probe responds to Zn^2+^ with distinct emission color change. The LOD for Zn^2+^ and Hg^2+^ was found to be 3.7 µM and 4.8 µM, respectively. Probe **19** demonstrated fluorescence bioimaging of raw cells, which was evident by the green fluorescence image of the raw cells in the presence of Zn^2+^ ions. Therefore, the probe could be used as an intracellular bio probe for dual sensing of ions.

**Scheme 18:**
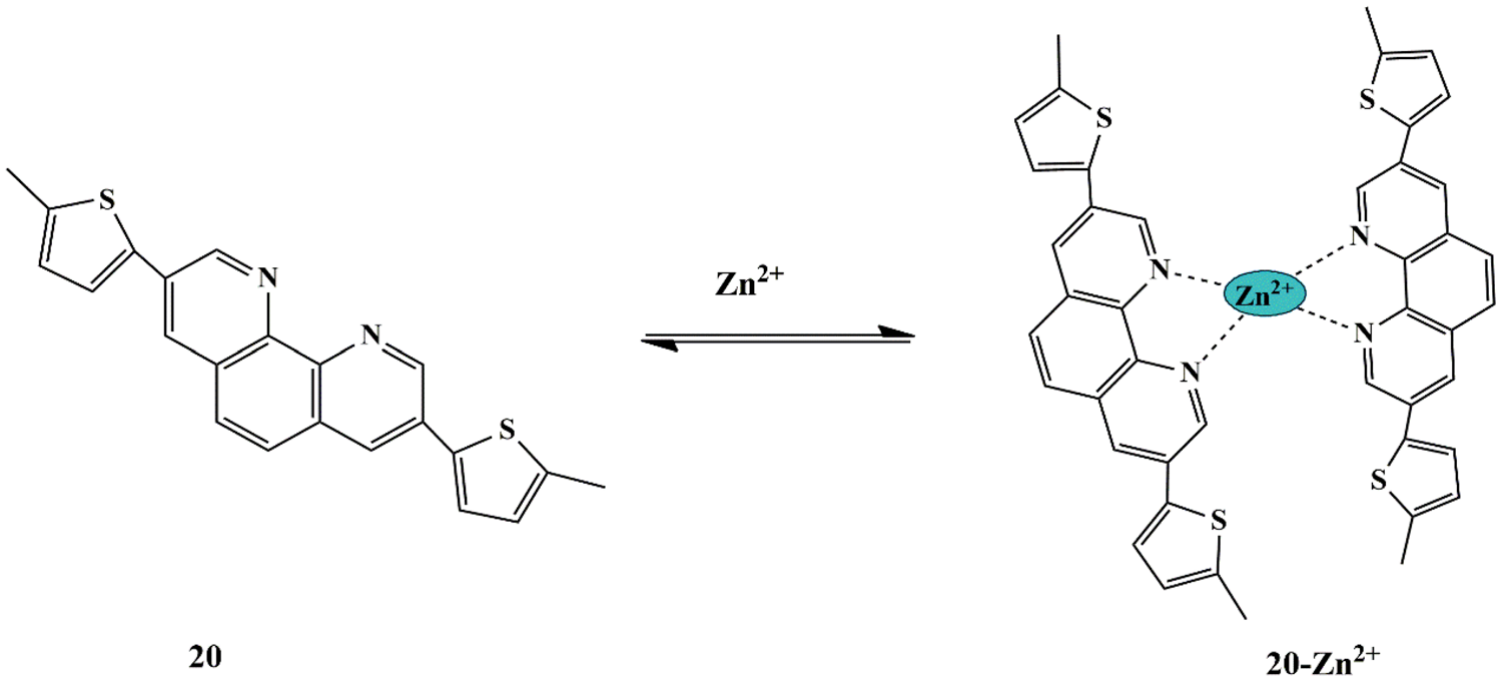
Suggested sensing mechanism of **20** towards Zn^2+^

Phenanthroline-based chemosensors, having strong chelating abilities and robust conjugated π-systems, have received enormous attention in recent years. Zhang et al. synthesized a novel phenanthroline-thiophene based chemosensor **20** for Zn^2+^ detection. The free sensor in Ethanol/water (v/v 9:1) exhibits an absorbance band at 282 nm, 359 nm, and 375 nm due to the ICT mechanism. Nevertheless, in the presence of Zn^2+^ ions, a new band is observed at 405 nm, accompanied by a red shift band, which is ascribed to LMCT between Zn^2+^ and probe **20** and enhanced ICT process, respectively. The fluorescence spectra of the free probe displayed weak fluorescence at 422 nm, whereas this emission band showed a bathochromic shift of 461 nm with an exceptional fluorescence upon the addition of Zn^2+^, which could be attributed to the CHEF effect due to the complexation of the two 5-methylthiophene groups at the 3 & 8 positions of phenanthroline with Zn^2+^ **(**Scheme 18**)**. The binding mode of probe **20**-Zn^2+^ was proposed to be 1:2 stoichiometry, confirmed by ESI-MS spectra. The probe **20** could also sense ClO_4_^−^ to a certain extent because of competitive coordination to Zn^2+^, thereby showing anionic interference, as evidenced by fluorescence quenching emission. Moreover, the probe could be employed in a broad pH range 2-10, evident by pH-dependent fluorescent [55] changes. Because of good cell permeability and reversible Zn^2+^ sensing property of the probe, it could be employed for practical applications such as biosensing of Zn^2+^ in living cells.

### Copper

Copper is a trace element required by the body for various activities, such as behaving as a catalytic cofactor for diverse metalloenzymes. The deficiency of copper in the body can lead to anemia, impaired organ function, and Menkes disease [56]. Contrarily, an increase in copper concentration beyond a limit can cause diarrhea, dyslexia, vomiting, headache, Wilson’s disease, Alzheimer’s, and Parkinson’s disease [57–59].

**Scheme 19:**
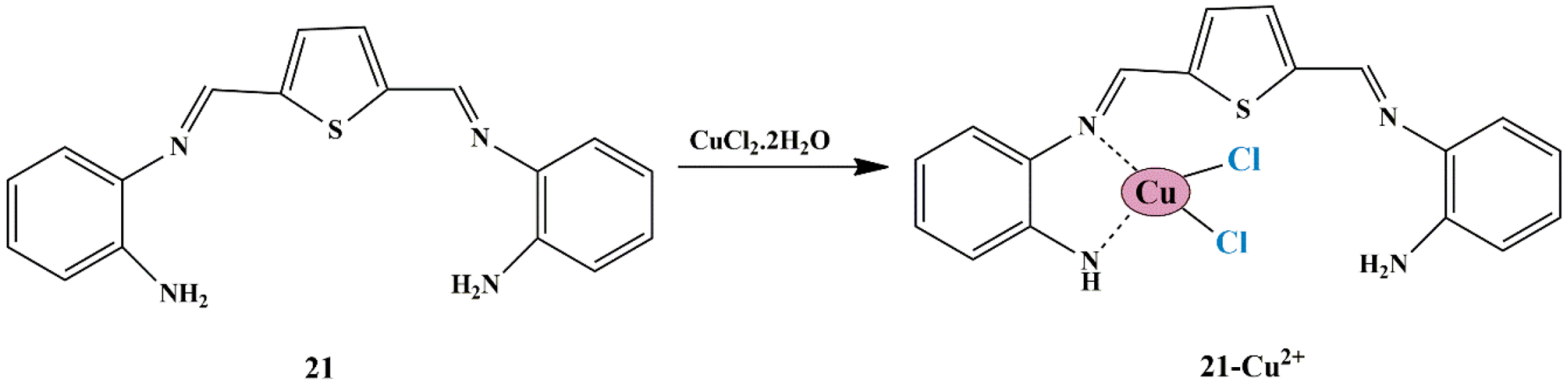
Binding mode of Receptor **21** to Cu^2+^

Encouraged by sorting out the above problems, Sivalingam et al. synthesized a ‘turn-on’ thiophene-based schiff base (receptor **21**). They evaluated its cation sensing properties in aqueous and biological samples since schiff base-type compounds are effectual metal chelators and could be utilized in fluorescent chemosensors. Receptor **21** could selectively detect Cu^2+^ in aqueous samples in the presence of other ions, as evidenced by the color change in the colorimetric analysis and an effective fluorescence response. The UV-Vis absorbance spectra revealed that a new band was obtained at 370 nm with Cu^2+^ ions (with the decrease in the band at 448nm exhibited by the receptor). The color and optical changes could be assigned to the coordination bond formed between receptor **21** and Cu^2+^ ion by transferring lone pair of electrons of amine and imine into the empty Cu^2+^ orbital **(**Scheme 19**)**. Evaluation of potential applications of the receptor as a bio-probe for Cu^2+^ detection in bio-imaging and bio-labeling research was achieved by demonstration of Cu^2+^ recognition in living cells, by using *E. coli*. It was found that receptor **21** could behave as a nanomolar (0.423 nM) Cu^2+^ sensor [57] with the binding constant of 4.23 × 10^5^, which closely matched with the results of DFT calculations.

**Scheme 20:**
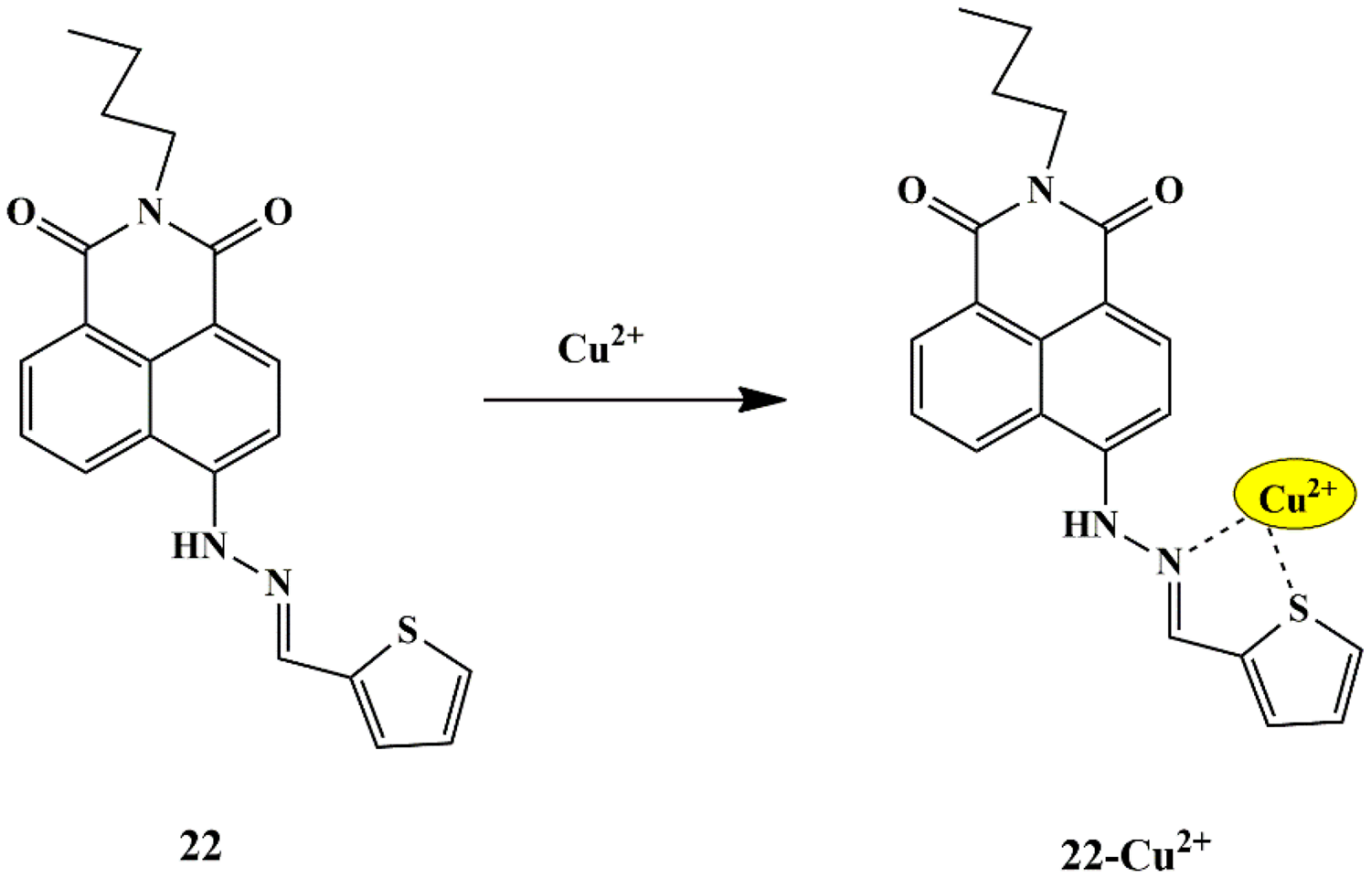
Plausible detection mechanism of probe **22** towards Cu^2+^

Using the same design guide, Guangijie et al. synthesized a new fluorescent schiff base probe **22** based on naphthalimide and thiophene moiety. The UV-Vis absorbance spectra displayed a drop in the absorption peak at 465 nm upon the addition of Cu^2+^ ions to the probe in CH_3_CN/HEPES (v/v 3:2) solution. The binding stoichiometry was determined to be in a 1:1 ratio, supported by ESI-MS spectra and Job’s plot. The probe was found to be effective in broad pH range, confirmed by pH-dependent studies. Fluorescence spectroscopic studies showed that probe **22** exhibited a strong fluorescence emission intensity at 575 nm, which quenched significantly only upon the inclusion of Cu^2+^ ions, which could be due to its paramagnetic nature **(**Scheme 20**)**. The LOD was determined to be 1.8 µM, and the probe showed an irreversible sensing mechanism upon EDTA addition as it failed to recover its fluorescent intensity. Furthermore, probe **22** could also be employed to detect Cu^2+^ ions in live cells [60], evidenced by the significant quenching of the green fluorescence, thereby demonstrating that the probe had the ability of fluorescence imaging in vivo.

**Scheme 21:**
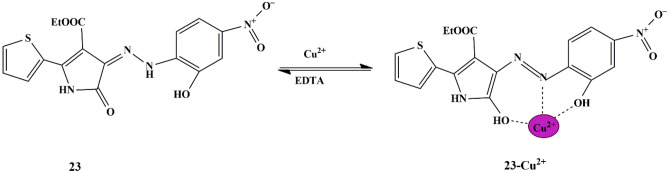
Possible mechanism for copper ion binding to probe **23**

Another novel approach was put forward by Ayesha et al., who synthesized a novel pyrrolinone ester derivative-based hydrazone dye **23** and employed it as a multi-functional probe. The fluorescence emission spectra of the probe in EtOH/water (v/v 1:1) displayed a significant quenching only for Cu^2+^ ions, thereby showing a ‘turn-off’ response due to probe **23**-Cu interaction [61]. Furthermore, the UV-Vis spectra provided a new characteristic absorbance band at 542 nm with a color change to purple that corresponds to the probe **23**−Cu^2+^ complex **(**Scheme 21**)**, and the absorbance band at 485nm (of Free probe) vanished. The detection limit was 0.85 µM, the association constant was reckoned to be 1.67 × 10^4^ M^−1^, with 1:1 stoichiometry. Reversibility studies were performed using EDTA, which suggested that the binding mode was reversible.

**Scheme 22:**
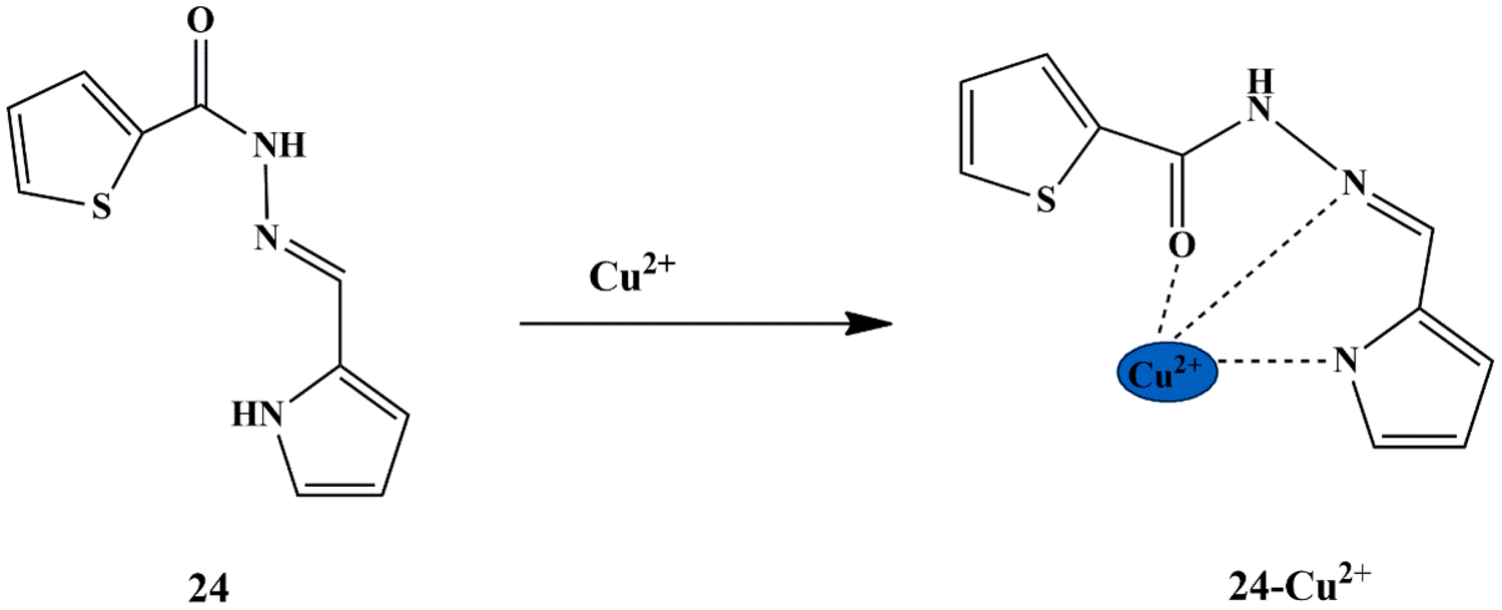
Suggested binding mode of sensor **24** to Cu^2+^ ions

**Scheme 23:**
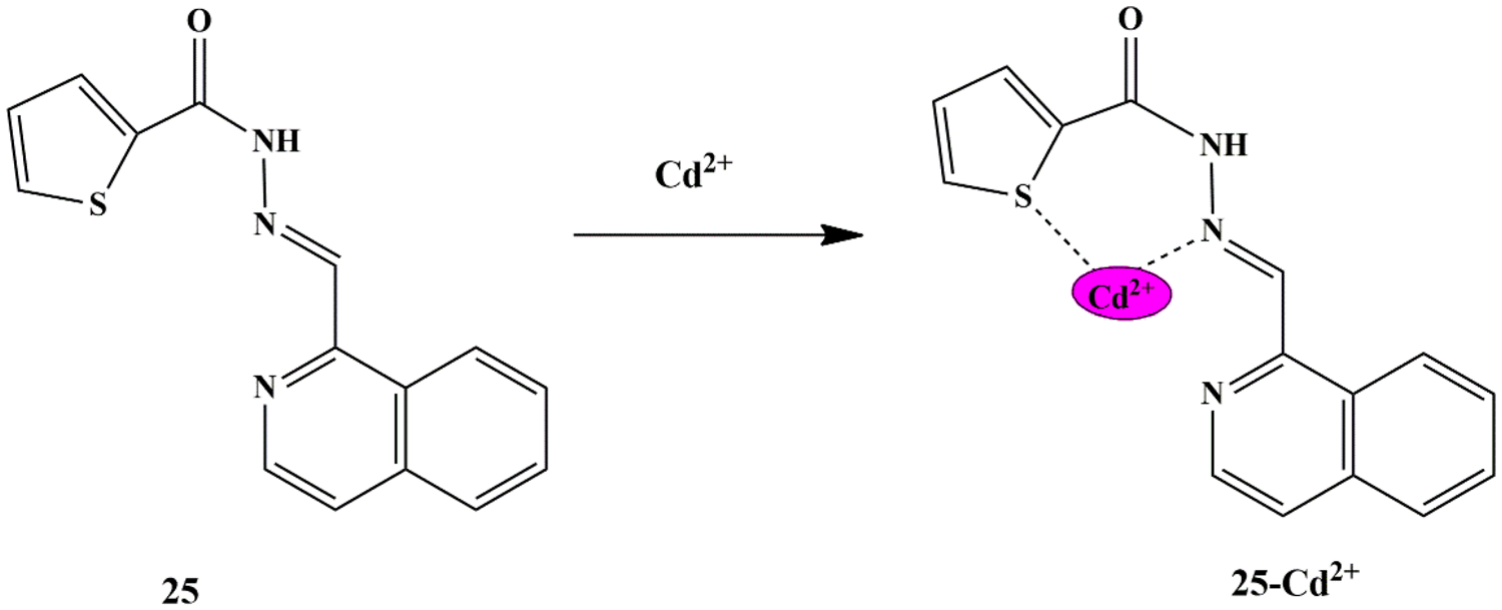
Suggested binding mode of sensor **25** to Cd^2+^

Tekuri and Trivedi synthesized a series of thiophene-2-carboxylic acid hydrazide-based chemosensors, among which **24** (Thiophene-2-carboxylic acid (1 H-pyrrol-2-ylmethylene)-hydrazide), and **25** (Thiophene-2-carboxylic acid isoquinolin-1-ylmethylene-hydrazide) responded for the detection of Cu^2+^ and Cd^2+^ ions. Chemosensor **24** displayed a striking yellow color only in the presence of Cu^2+^ ions, and chemosensor **25** exhibited a yellow appearance only in the presence of Cd^2+^, as evidenced by the UV-Vis titration study which showed a decrease in the absorbance intensity of **24** at 335 nm and **25** at 320 nm, and a new absorbance band at 402 nm (in **24**), 425 nm (in **25**) upon titration with Cu^2+^ and Cd^2+^ ions, respectively. These changes are attributable to the change in structural conformation of chemosensors upon interaction with Cu^2+^ and Cd^2+^, thereby demonstrating a coordinative interaction between the oxygen of carbonyl group and imine nitrogen in probe **24**, whereas Sulphur and nitrogen in probe **25** respectively **(**Scheme 22 and 23**)**. It was found that probe **24** and probe **25** exhibited a dynamic linear absorption response range, from 0 to 50 µM for Cu^2+^ ions and 0-30 µM for Cd^2+^ ions, with the LOD calculated to be 2.8 × 10^−6^ M and 2.0 × 10^−7^ M for Cu^2+^ and Cd^2+^ ions respectively [62]. The association constant was determined to be 7.8 × 10^3^ M^−1^ for probe **24**−Cu^2+^ and 3.7 × 10^4^ M^−1^ for probe **25**−Cd^2+^, and the respective binding was found to be in 1:1 stoichiometry.

**Scheme 24:**
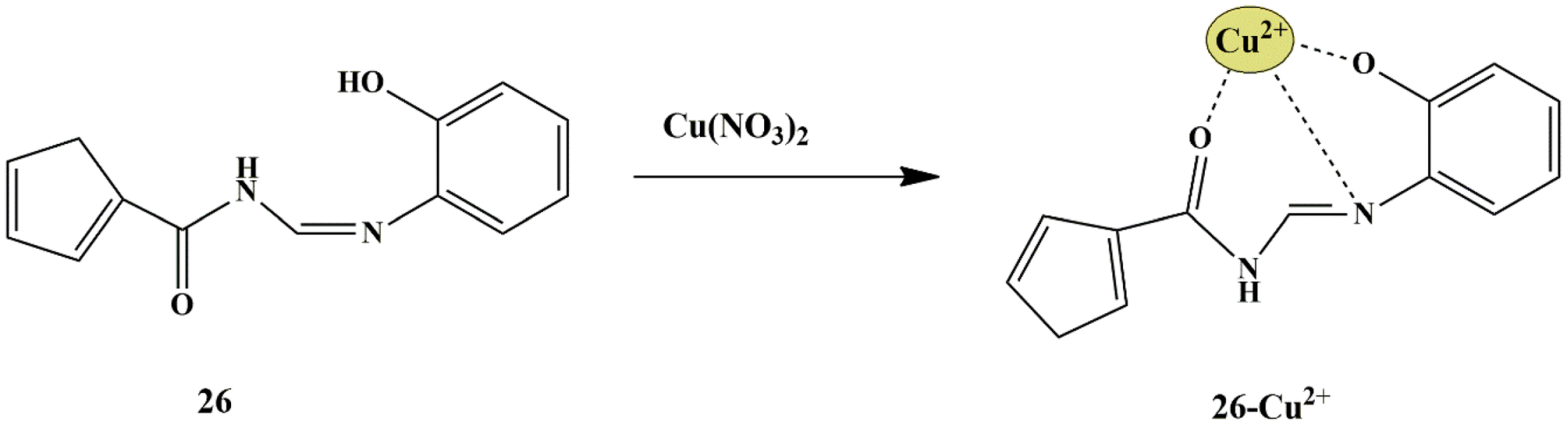
Sensing mechanism of probe **26** towards Cu^2+^

To detect copper in the aqueous system, Penchang et al. synthesized a thiophene-based colorimetric sensor **26**. From the UV-Vis absorption spectra results, it was evident that only Cu^2+^ increased the absorption band of the probe by 10 folds and displayed a prominent change in color from colorless to yellow in DMSO/H_2_O (v/v 1:1) solution due to the probe **26**-Cu^2+^ complex formation; whereas fluorescence quenching was observed due to the paramagnetic nature of Cu^2+^ ions. The probe also exhibited anti-interference ability for the detection of Cu^2+^, and the working of the probe was found to be in a broad pH range (6-10). The binding constant was determined to be 6.55 × 10^4^ M^−1^ with 1:1 stoichiometry of probe **26**-Cu^2+^ complex **(**Scheme 24**)** and the LOD was determined to be 0.217 µM. Furthermore, to scrutinize the practical applications of the probe, the TLC-based strip tests were executed by employing Cu^2+^ solution in water. After introducing Cu^2+^ on the TLC plate (immersed in probe in DMSO/H_2_O), color change from colorless to yellow [63] was noticed under sunlight, implying that probe **26** could function as a suitable tool for selective detection of Cu^2+^ ions.

**Scheme 25:**
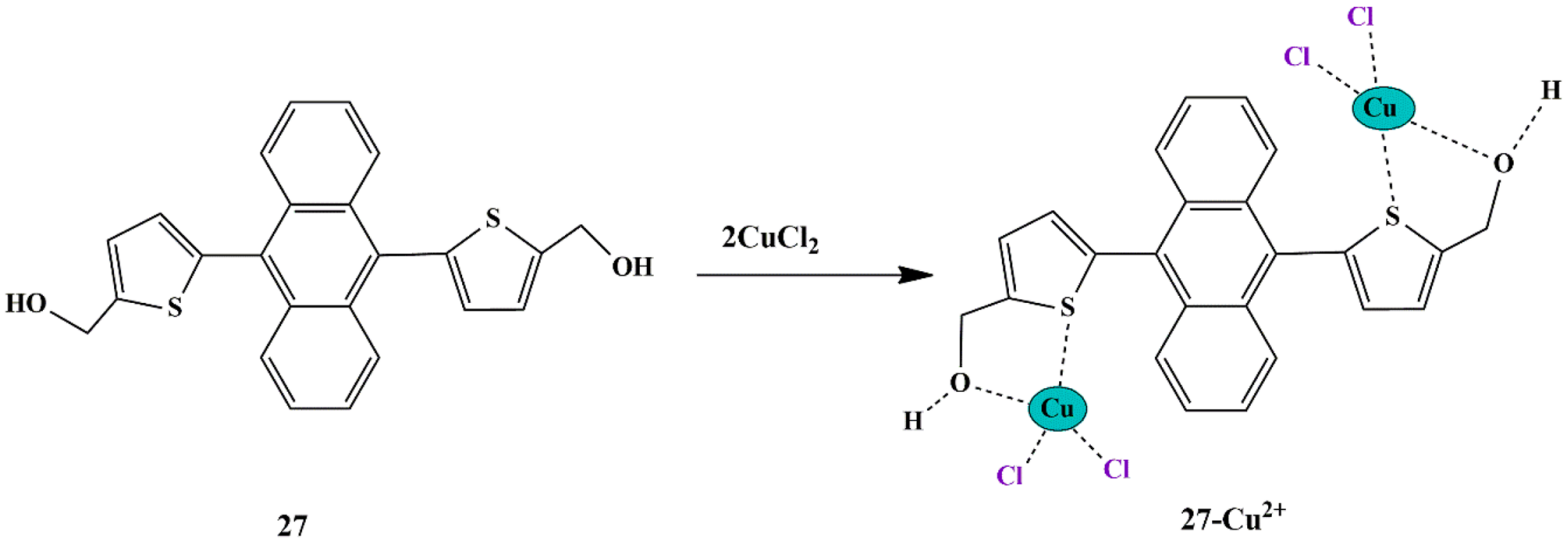
Photo-sensing mechanism of probe **27** towards Cu^2+^

Thuy et al. synthesized thiophene appended anthracene-based fluorescent probe **27** for the detection of CuCl_2_. The probe was able to explicitly identify CuCl_2_ from other metals since other metals exhibited intense blue fluorescence emission in the UV-Vis spectra. The fluorescence intensity at 460 nm decreased upon the introduction of 1mM CuCl_2_ in DMSO solution, and complete quenching could be observed at 30 mM CuCl_2_, which could be attributed to the overlapping of the fluorescence wavelength of probe **27** and the absorption wavelength of CuCl_2_ **(**Scheme 25**)**. The binding between CuCl_2_ and probe **27** was found to be in 1:2 stoichiometry [64] and the binding constant was found to be 0.0895 mM^−2^.

### Silver

Silver has been valued as a precious metal and has gained immense significance due to its potential applications in medicine, electronics and photography, catalysis, and nano-chemistry [65]. Due to its widespread use, it possesses various entry routes into the body, primarily through ingestion [66], inhalation [67], and skin contact. Excessive silver ions can integrate with amine, imidazole, and carboxyl moieties of different metabolites in the cytosol, consequently leading to undesirable effects on humans. Long-term exposure to silver can cause Argyrosis and Argyria [68].

**Scheme 26:**
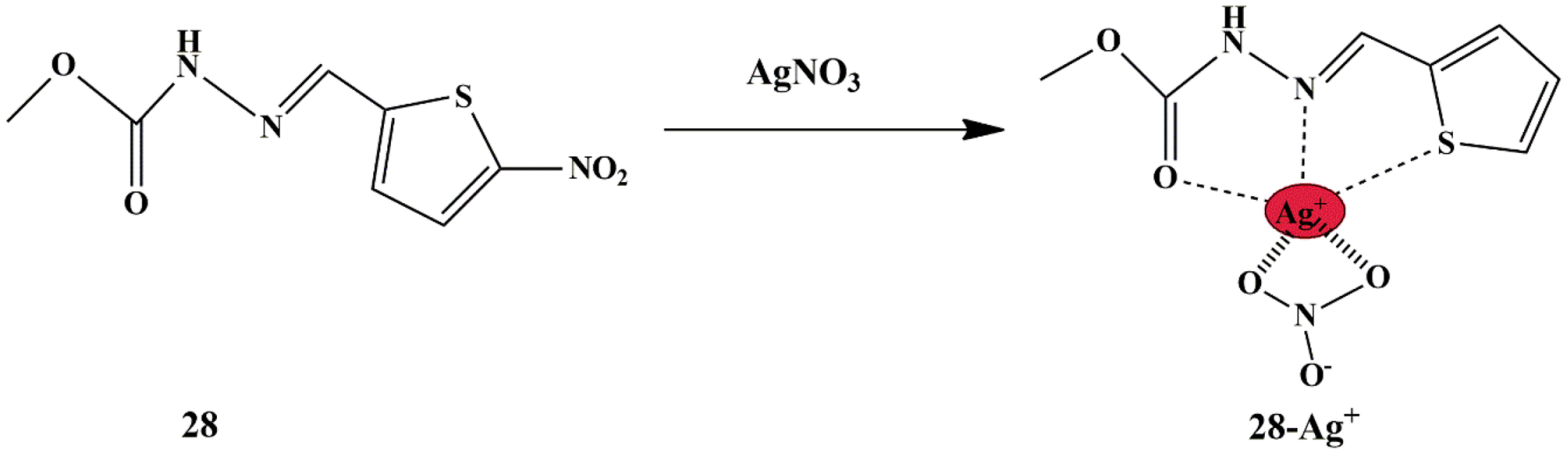
Proposed binding mechanism of probe **28** to Ag^+^

Bhuvanesh et al. synthesized thiophene-based probe **28**, which could detect Ag^+^ in aqueous media. There was a substantial fluorescence enhancement of probe in CH_3_OH/H_2_O (v/v 1:1) solution, indicating the possibility of ICT mechanism due to the coordination of Ag^+^ with probe **28** in 1:1 binding stoichiometry. The pH and time response studies revealed the effectiveness of the probe over a broad pH range and a rapid response time, suggesting that it could be employed for Ag^+^ detection in environmental and biological samples. The interaction is possible through ICT aided by the restricted torsional rotation between the C−C single bond of the probe **(**Scheme 26**)**, which was confirmed by mass spectra and the LOD was estimated to be 1.28 × 10^−7^ M.

Moreover, to test and evaluate the potentiality of the probe in the detection of Ag^+^ in biological applications, microscopic images of *E. coli* cells in the absence and presence of probe **28** and Ag^+^ were compared; and observed that in the presence of Ag^+^, the probe displayed highly intense red fluorescence due to the probe [**28**-Ag^+^] complex inside the cells, thereby enhancing the fluorescence intensity and it was hereby concluded that the probe could be employed in live-cell fluorescence imaging [69] for Ag^+^ detection. Additionally, it was observed that the probe could also be used for monitoring Ag^+^ ions in natural water samples, thereby paving a way for Ag^+^ detection in both Aqueous and biological samples.

**Scheme 27:**
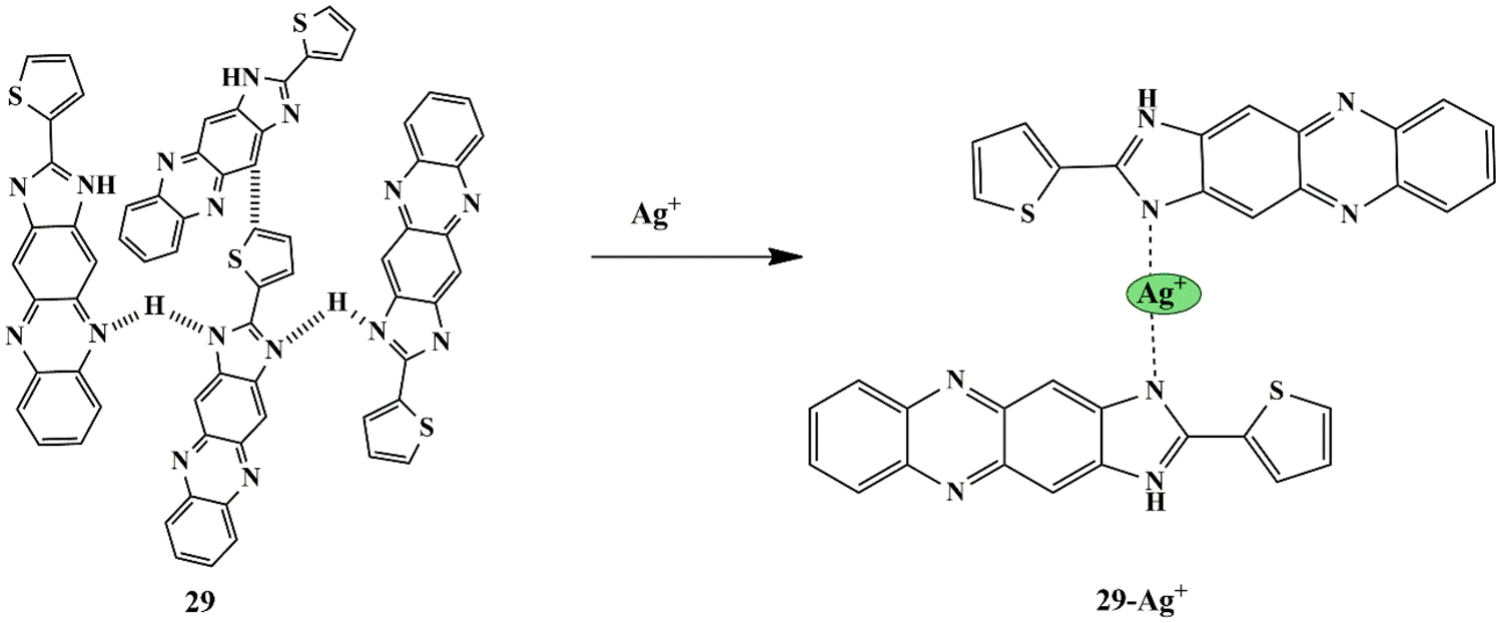
Possible sensing mechanism of probe **29** towards Ag^+^

A successful novel approach was made by Xeong et al. who developed a thiophene-functionalized imidazophenazine based self-assembled supramolecular sensor **29** to evaluate the recognition towards Ag^+^. The sensor reacted with a color change from yellow to light purple upon introducing Ag^+^ ions in DMSO/Water solution, accompanied by a redshift to 376 nm. Moreover from the fluorescence emission spectra, it was also noticed that the fluorescence intensity of the peak at 547 nm abruptly decreased with the addition of the Ag^+^ to the solution, confirming the selective sensing and detection of Ag^+^ ions over other interfering metal ions [70] **(**Scheme 27**)**. Moreover, the ‘on-off’ mechanism was evaluated by the addition of I^−^ ions to test the reversibility of the sensing mechanism, and was observed that the sensor could be considered an excellent on-off fluorescence sensor, which was evident by the alternating reviving and quenching processes in the fluorescence emission of the sensor **29**-Ag^+^ solution with 2:1 binding stoichiometry.

The LOD was found to be 8.18 × 10^−9^ M, which was much lower in comparison to the previously reported chemosensors.

### Calcium

Calcium is the fifth most abundant element in the human body and is a significant component of the bone, where it is stored as calcium phosphate. Calcium ions partake in the biochemical and physiological processes of the cells. In contrast to its benefits, excess calcium in the biological system can result in hypercalcemia, leading to abdominal pain, bone weakening, muscle cramps, and other neurological symptoms.

**Scheme 28:**
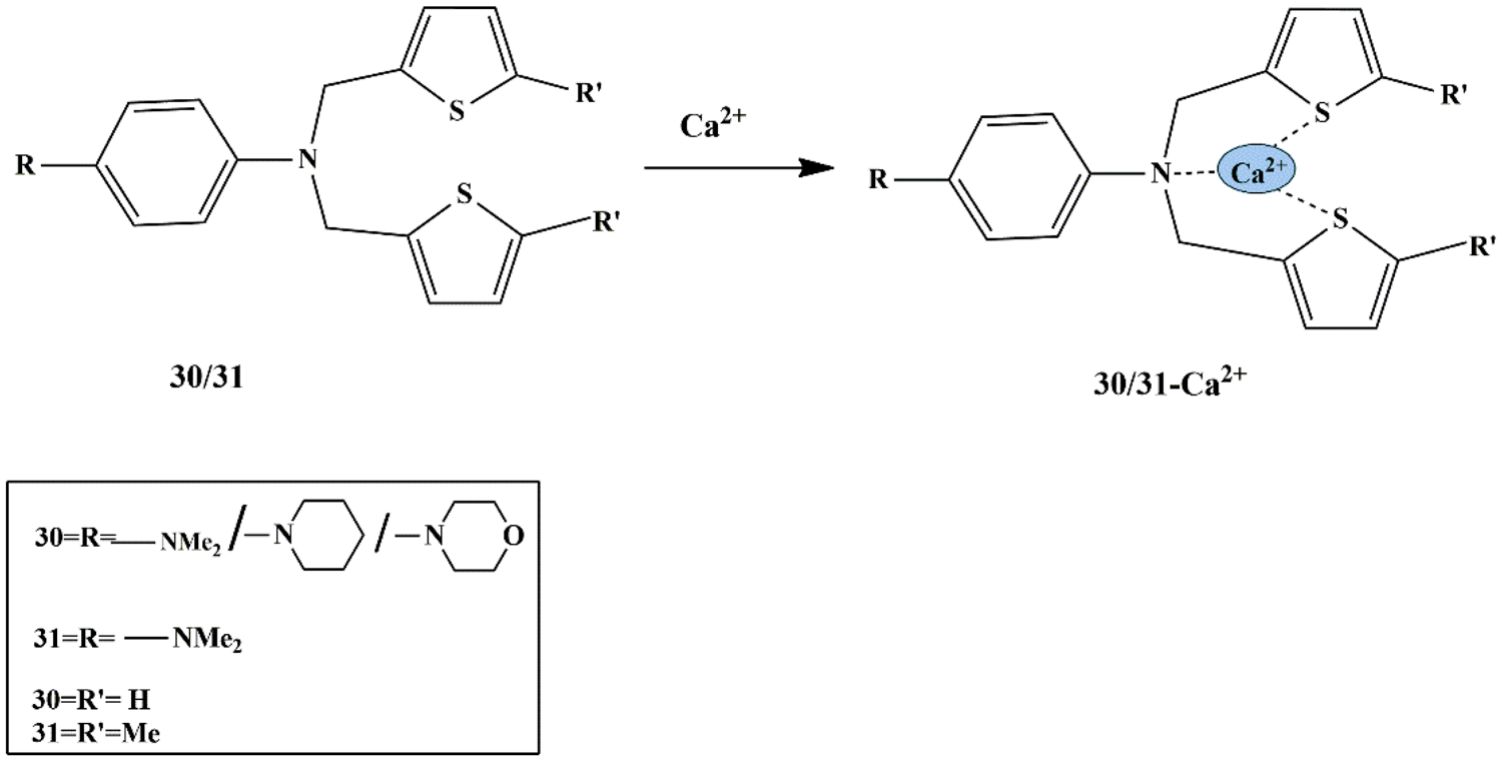
Binding of Ca^2+^ to sensor **30/31**

To achieve chemosensors for the selective binding of calcium ions, Rihab et al. synthesized two electroactive tetra-alkylated p-phenylenediamine chemosensors (**30** and **31**) relying on thiophene ligands. The cyclic voltammetry studies revealed that both chemosensors were selectively able to bind Ca^2+^ ions over Zn^2+^ ions. The free ligand exhibited two peaks, but only one peak was observed at a higher potential upon adding Ca^2+^ ions. This could be ascribed to the oxidation of the Calcium complex, which is formed between Ca^2+^ and two thiophenyl moieties and the nitrogen atom **(**Scheme 28**)**. Furthermore, the calcium chelation resulted in a significant change in the transducer by larger anodic potential shifts, and the metal to ligand ratio [71] was found to be 1:2 suggesting a [Ca(L)_2_]^2+^ complex formation. Chemosensor **30**, having the replacement of the dimethylamino group with the piperidinyl group, showed high selectivity towards Ca^2+^ detection, evidenced by electrochemical studies.

### Zirconium

Zirconium is widely used in ceramics, metal alloys, foundry equipment, the chemical processing industry, and electronic devices. Although, their regular use can produce high levels of residual zirconium ions, which consequently results in soil and water contamination. Inhaling zirconium compounds can give rise to pulmonary granulomas, skin and lung granulomas. Accordingly, it is crucial to develop chemosensors having high sensitivity and selectivity for the instantaneous detection of Zr^4+^ ions in environmental and biological samples.

**Scheme 29:**
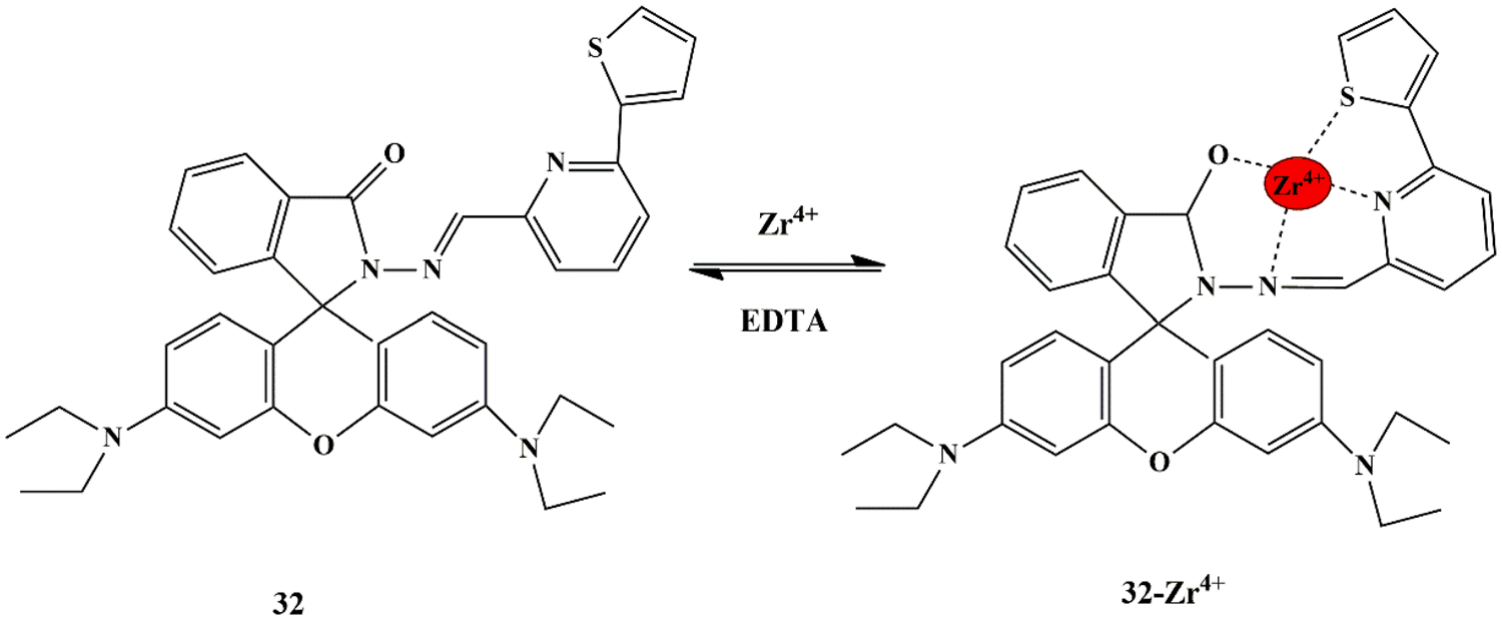
Proposed sensing mechanism of probe **32** towards Zr^4+^

In this regard, Ajit et al. synthesized a novel pyridine-thiophene appended rhodamine-based probe **32**, and the recognition properties of the probe towards different metal ions were evaluated by spectroscopic studies using CH_3_OH/H_2_O (v/v 4:1) mixture. In the absence of metal ions, the solution of the probe was weakly fluorescent and colorless because of the ring-closed spirolactam, which then turned into deep orange upon introduction of Zr^4+^, due to the spirolactam-ring opening and generation of the delocalized xanthene moiety **(**Scheme 29**)**. Emission and absorption titrations of probe **32** display fragile spectral characteristics in the emission spectrum, whereas the introduction of Zr^4+^ ions enhanced the fluorescence intensity significantly with an intense orange fluorescent emission band at 582 nm. Moreover, the sensing mechanism was reversible upon adding EDTA due to the disappearance of the formed complex, and the LOD was calculated to be 16.99 µM.

Additionally, to demonstrate the practical bio-sensing capability of the probe, fluorescence imaging experiments were carried out in vivo in *C. albicans* cells. Fluorescence imaging was evaluated, which showed that probe **32** alone was non-emissive, but pollen cells loaded with Zr^4+^ exhibited red fluorescence from the intracellular area. The evidence of the viability of cells (after loading with probe and Zr^4+^) throughout the imaging experiment was confirmed by Bright field microscopy [72], indicating that the probe could be used for examining intracellular Zr^4+^ in living cells.

### Iron

Iron is the most necessary microelement responsible for the proper functioning of physiological processes in living organisms. Iron is an essential constituent of hemoglobin and myoglobin and is also required for growth, neurological development, cellular functioning, and synthesis of some hormones. However, excess Iron can cause Iron poisoning, whose symptoms include diarrhea, vomiting, abdominal pain, and other ill effects on humans.

**Scheme 30:**
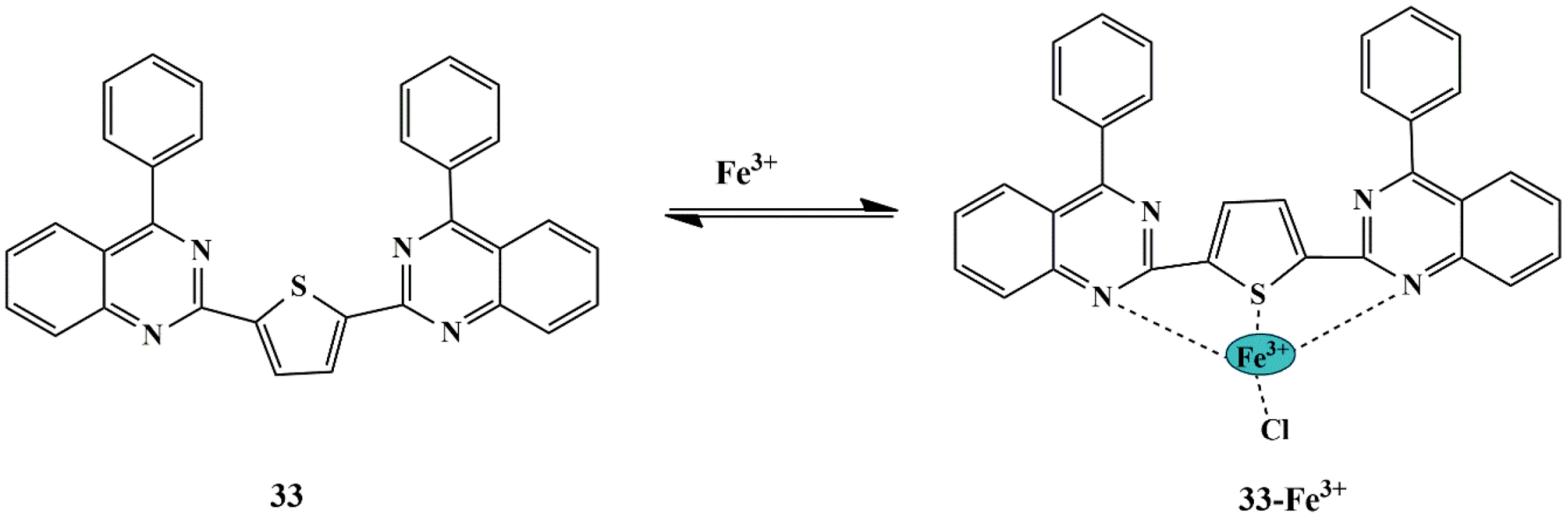
A suggested possible mechanism for sensing of Fe^3+^

To develop fluorescent chemosensors to detect Fe^3+^, Kannikanthi et al. synthesized a fluorescent chemosensor **33** [2,5-bis(4-phenylquinazolin-2-yl)thiophene]. The free sensor in CH_3_CN displayed an absorption band at 362 nm, and it exhibited a color change from colorless to greenish-yellow, and an occurrence of a new band at 419 nm, only in the presence of Fe^3+^ ion. Fluorescence quenching at 427 nm upon the addition of Fe^3+^ ions, which could be attributed to the ICT between thiophene and phenylquinazoline units along with the MLCT **(**Scheme 30**)** between the sensor and Fe^3+^ ion in 1:1 binding mode. The LOD was calculated to be 1.6 × 10^−8^ M. Reversibility equips the reusability of the chemosensor in practical applications, and hence reversibility studies were evaluated using EDTA. Upon addition of EDTA to the sensor **33**-Fe^3+^ complex, the fluorescence of sensor **33** is reimposed, thereby implying that the probe could be employed as a reversible sensor for Fe^3+^ ions. Furthermore, to investigate its applications in nanoscale computations, the probe could also be employed to construct an inhibit-type logic function with Fe^3+^ and EDTA being the chemical inputs and fluorescence emission [73] outputs at 533 nm.

**Scheme 31:**
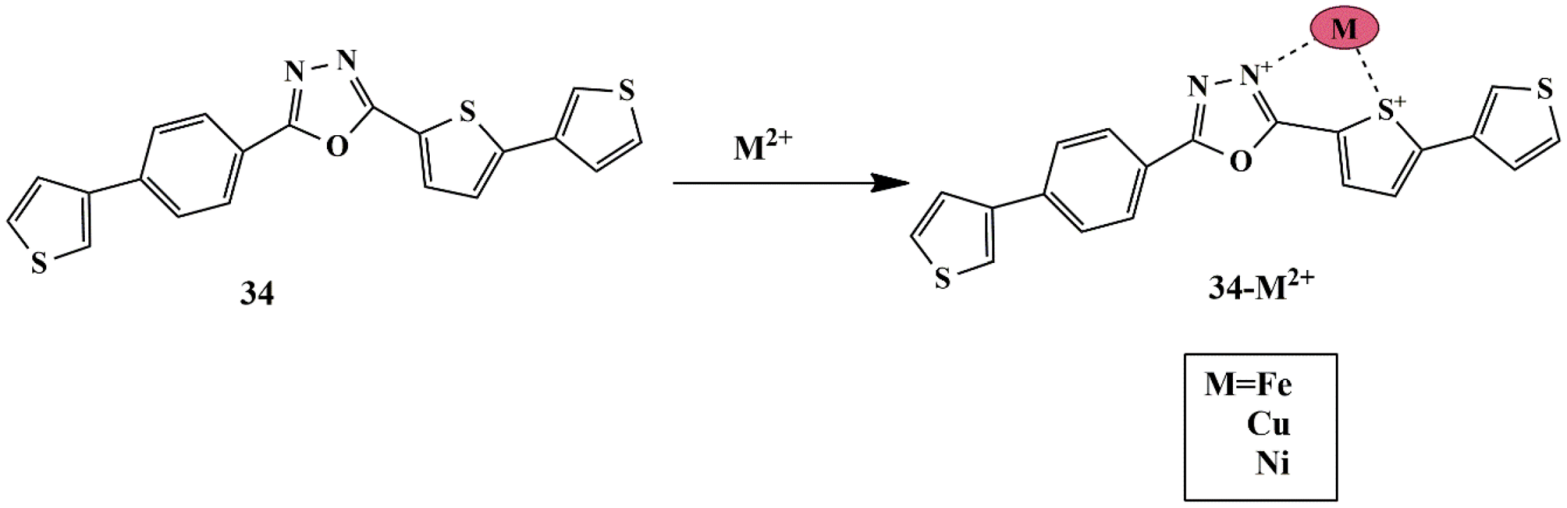
A proposed binding mechanism for **34** towards Fe^2+^/Ni^2+^/Cu^2+^

Considering high photoluminescence properties of 1,3,4-oxadiazole moiety, Lohit Naik et al. synthesized a turn-off fluorescent chemosensor based on thiophene substituted 1,3,4-oxadiazole derivative **34**, for the detection of multiple metal ions. The sensitivity of probe **34** in C_2_H_5_OH/water, CH_3_OH/water, and CH_3_CN/H_2_O mixture (v/v 1:1) at pH 7.2, was investigated by UV-Vis absorbance spectroscopy. It was observed that, in all three mixtures, the absorbance peak and fluorescence emission peak of the probe were seen at 275 nm at 405 nm, respectively. It was observed that in the C_2_H_5_OH /H_2_O mixture, the fluorescence emission quenched to a greater amount upon the addition of Fe^2+^, owing to the paramagnetic nature of Fe^3+^ and could be ascribed to the CHEQ mechanism. In CH_3_CN/H_2_O and CH_3_OH/H_2_O mixture, the absorbance peak accounted maximum for Cu^2+^ at 225 nm and Ni^2+^ at 400 nm, respectively, due to the CHEF mechanism **(**Scheme 31**)**. The binding of probe and metal ions was in 1:1 stoichiometry and the detection limit for Fe^2+^, Ni^2+^, and Cu^2+^ was calculated to be 2.977 × 10^−6^ M, 0.895 × 10^−6^ M, 0.593 × 10^−6^ M, respectively. The detection time was quick [74] (<6.5 min) signifying that probe **34** can be implemented for the rapid detection of multiple metal ions.

**Scheme 32:**
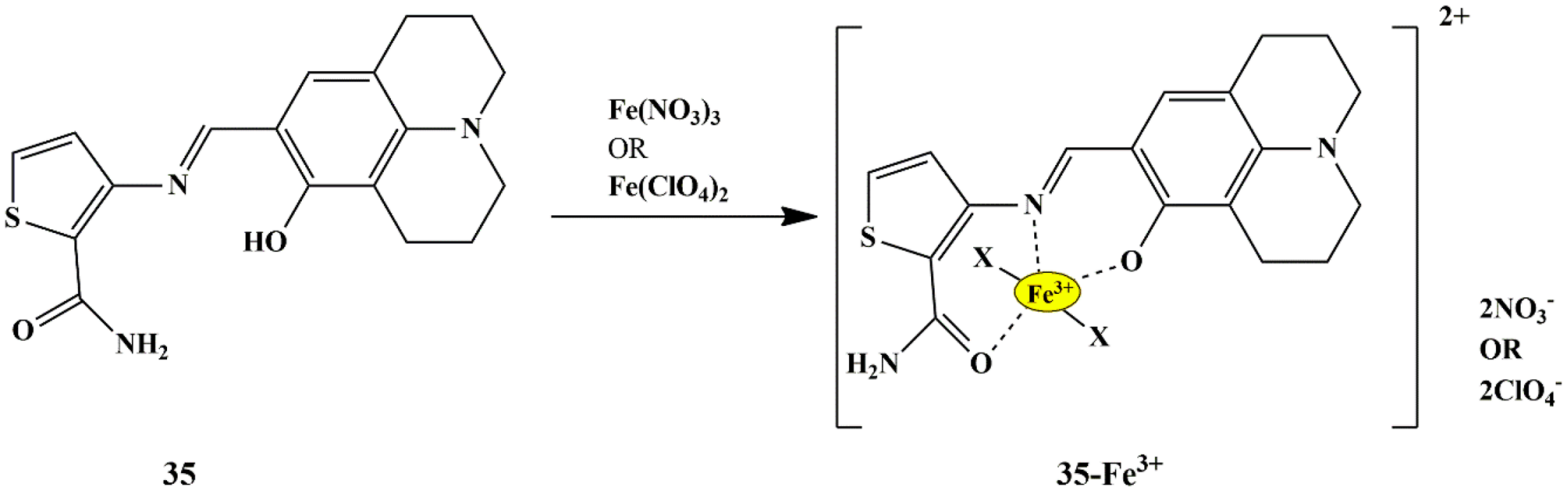
Proposed binding mechanism of sensor **35** to Fe^3+^

Owing to the paramagnetic nature of Fe^3+^, earlier reported fluorescent chemosensors functioned through fluorescence quenching mechanism, which was a considerable drawback in case in-situ monitoring and in vivo imaging [75]. As an out-turn, fluorescent and colorimetric probes operating by ‘turn-on’ response gained an enormous amount of attention. One such representative was the thiophene-based colorimetric sensor **35**, developed by Jung et al. for the sequential detection of Fe^3+^/^2+^ in a near-perfect aqueous solution. The sensor in bis-tris buffer showed distinct visual color changes from yellow to brown only upon the addition of Fe^3+^/^2+^, with significant spectral changes due to MLCT mechanism **(**Scheme 32**)** and a 1:1 metal ion/ligand complexation was proposed. The sensor could sense Fe^3+^ at pH range 4-8, which was confirmed by pH-dependent studies, and could be employed as a recyclable sensor to detect Fe^3+^ as evidenced by the reversibility studies using EDTA [76]. The LOD for Fe^3+^ and Fe^2+^ was determined to be 0.51 µM and 1.51 µM, respectively, which was lower than the EPA (Environmental Protection Agency) guideline for drinking water, indicating that sensor **35** could be employed in the real-time monitoring of Fe^3+^ in drinking water.

**Scheme 33:**
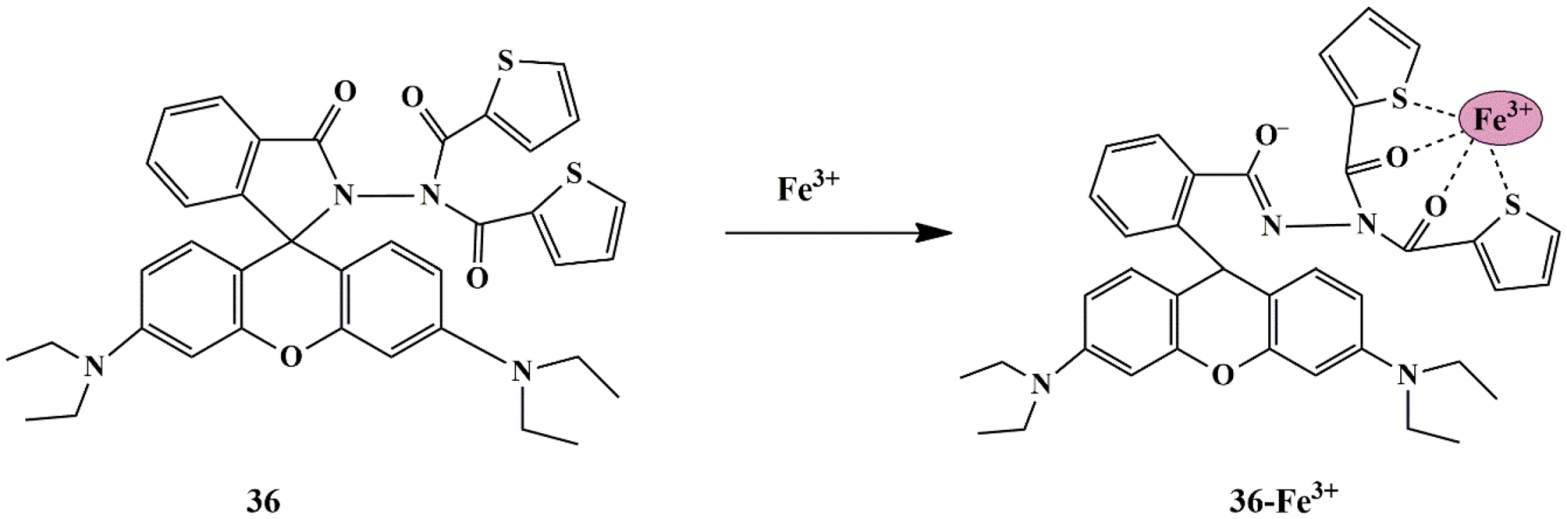
Proposed binding mechanism of probe **36** to Fe^3+^

As an innovative approach, Lieu et al. designed an N,N-dithenoyl-rhodamine based probe **36**, for Fe^3+^ recognition. All the tested cations except Fe^3+^ didn’t display any change in fluorescence spectra of the probe solution (CH_3_OH/H_2_O v/v 1:1) due to the closed conformation of the rhodamine spirolactam, whereas Fe^3+^ ions showed a strong fluorescence band complemented by an immediate color change from colorless to pink, due to the ring-opening reaction of rhodamine spirolactam **(**Scheme 33**)**. The fluorescence titration revealed that increasing Fe^3+^ amount results in an apparent peak at 588 nm, which further increased along with the color change from colorless to pink, suggesting the ring-opening of the rhodamine spirolactam [77] and the increased coordination interactions between Fe^3+^ ions and Oxygen and Sulphur on the three amides and thiophene of probe **36** respectively, as confirmed by H-NMR analysis. It was found that the LOD was 3.74 µmol/L, and the binding stoichiometry between the probe **36**-Fe^3+^ complex was 1:1 ratio. Additionally, the probe could effectively detect Fe^3+^ ions in living cells with good membrane permeability and less toxicity.

**Scheme 34:**
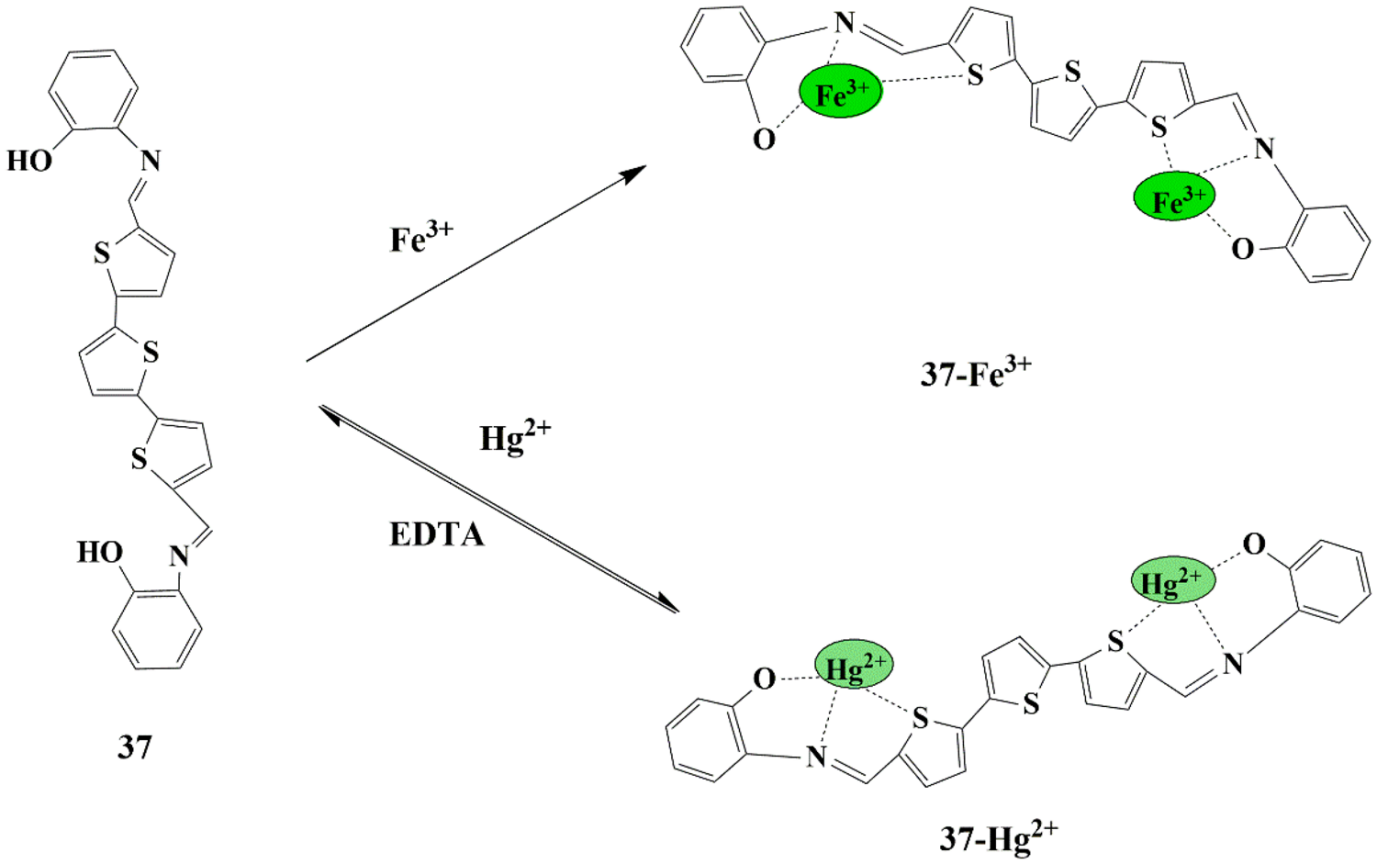
Detection mechanism of probe **37** for Fe^3+^/Hg^2+^ ions

Very few multifunctional or dual-mode fluorescent chemosensors have been designed for the detection of Fe^3+^ and Hg^2+^ in aqueous media, and hence in this regard, Tau Sun and his colleagues synthesized an oligothiophene- Schiff based chemosensor **37**, which could detect both Hg^2+^ and Fe^3+^ simultaneously. The fluorescence spectral studies were performed in CH_3_CN/H_2_O (v/v 4:6) since it was found that the probe was partially water-soluble. The usage of organic phase guarantees higher availability of sensor molecules in the medium. The free sensor exhibited weak fluorescence, which could be imputed to the PET process [78]. However, the addition of Fe^3+^/Hg^2+^ ions to the sensor solution displayed an exceptional fluorescence enhancement at 520 nm due to the CHEF effect, and the fluorescence intensity increased with an increase in the amount of Fe^3+^/Hg^2+^ ions, which could be attributed to the reduced PET process as a result of the complexation of sensor **37** with Fe^3+^/Hg^2+^ **(**Scheme 34**)**. The results obtained from the fluorescence spectra were in conformity with the results of the naked-eye experiment, which displayed the color change from light yellow to fluorescent green in the presence of Fe^3+^ and Hg^2+^ ions. The LOD for Fe^3+^ /Hg^2+^ was estimated to be as 3.52 × 10^−8^ M and 5.0 × 10^−8^ M. Besides, binding interactions studied by NMR, FT-IR spectral analysis, indicated a 1:2 metal/ligand binding stoichiometry and evidenced that phenol Oxygen, aldimine Nitrogen, and thiophene Sulphur of sensor **37** was entailed in the complex formation with Fe^3+^/Hg^2+^. Reversibility studies in EDTA indicated the irreversible binding interaction between the chemosensor and Fe^3+^ and reversible interaction with Hg^2+^. Furthermore, the fluorescence intensity of the sensor **37**–Fe^3+^/Hg^2+^ complex exhibits a ‘turn-on’ emission in a pH range (4-12) and persists in the ‘turn-off’ emission in acidic pH (pH < 4), suggesting that the sensor could be employed in a wide pH range, by exhibiting distinct ‘on-off’ responses.

### Cobalt

Cobalt is an ultra-trace element in the biological system and is a key constituent of cobalamin, better known as Vitamin B_12_, and is required for the proper metabolism of iron, and plays a crucial role in the hemoglobin synthesis [79]. Nevertheless, high or long-term exposure to cobalt can result in cancer, respiratory disorders, skin problems such as dermatitis, reduced cardiac output.

**Scheme 35:**
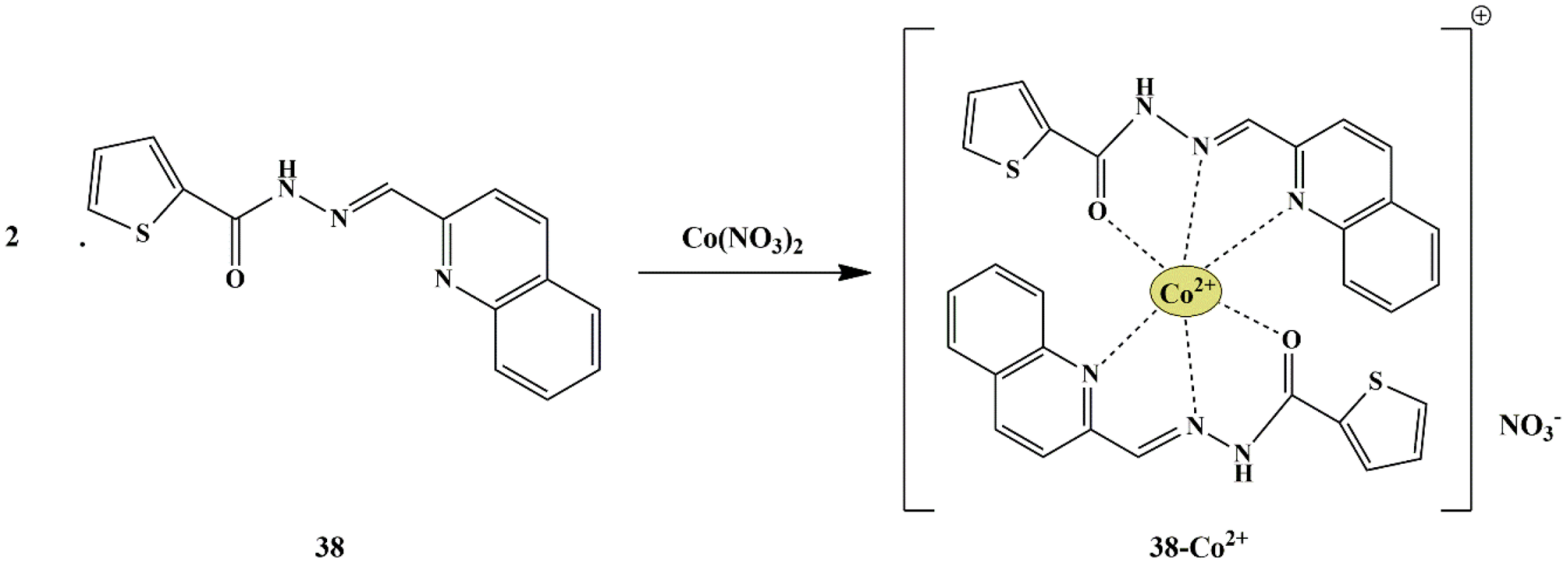
Proposed binding mode of Chemosensor **38** to Co^2+^ (2:1)

**Scheme 36:**
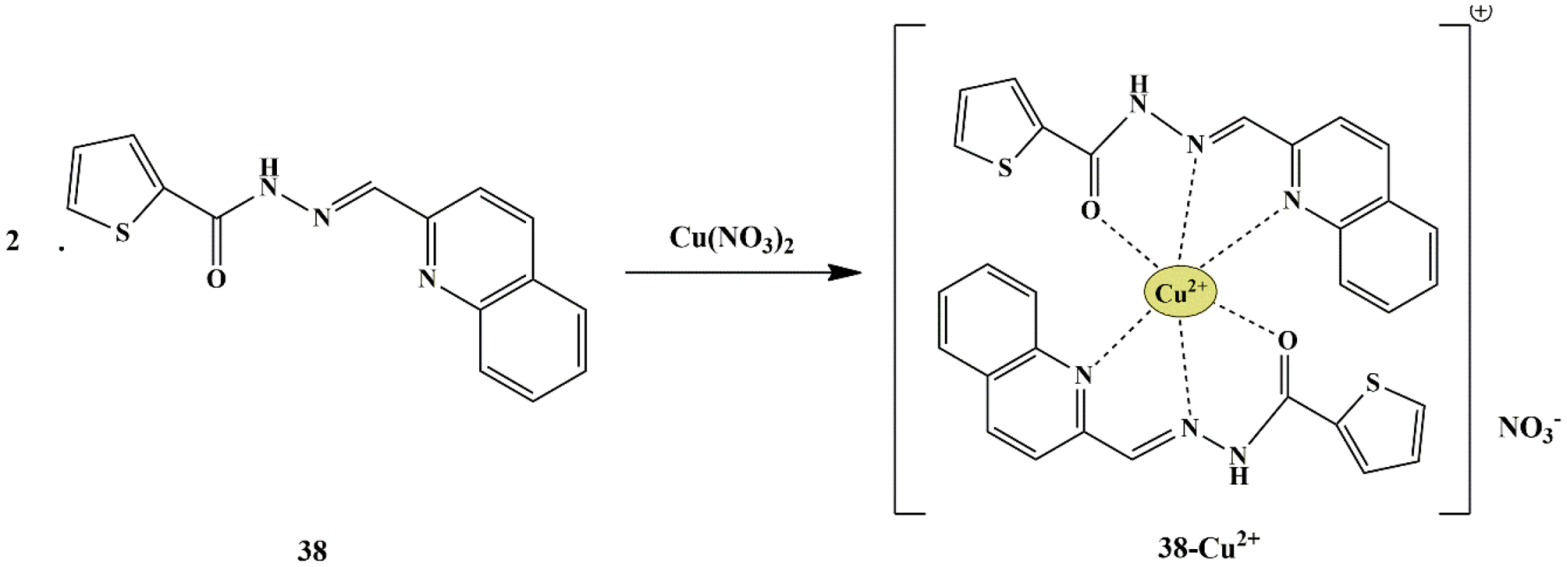
Proposed binding mechanism of Chemosensor **38** to Cu^2+^

In this regard, Jung et al. synthesized thiophene-2-carbohydrazide based chemosensor **38** and evaluated its sensing properties in aqueous media. The colorimetric detection of the sensor was investigated towards different metal ions at 400 nm in tris-buffer/DMSO (v/v 95:5) mixture. Amidst the tested metal ions, only Co^2+^ and Cu^2+^ exhibited immediate color change from colorless to yellow, in addition to distinct spectral changes. The UV-Vis titration revealed that the absorption band of chemosensor at 325 nm decreased remarkably, with the appearance of a new band at 400 nm, only for Co^2+^ which can be attributed to the co-ordination of chemosensor with Co^2+^ in 2:1 stoichiometry, further promoting Ligand to Metal charge transfer (LMCT) **(**Scheme 35 and 36**)**. The LOD for Co^2+^ was calculated to be 0.19 µM and the binding of chemosensor **38** with Co^2+^ and Cu^2+^ was found to be reversible with a chelating agent such as EDTA. Besides, the Co^2+^−2. (sensor **38**) complex can be employed as a colorimetric sensor for CN^−^, which was evident from a color change of yellow to colorless [80] in the aqueous solution.

**Scheme 37:**
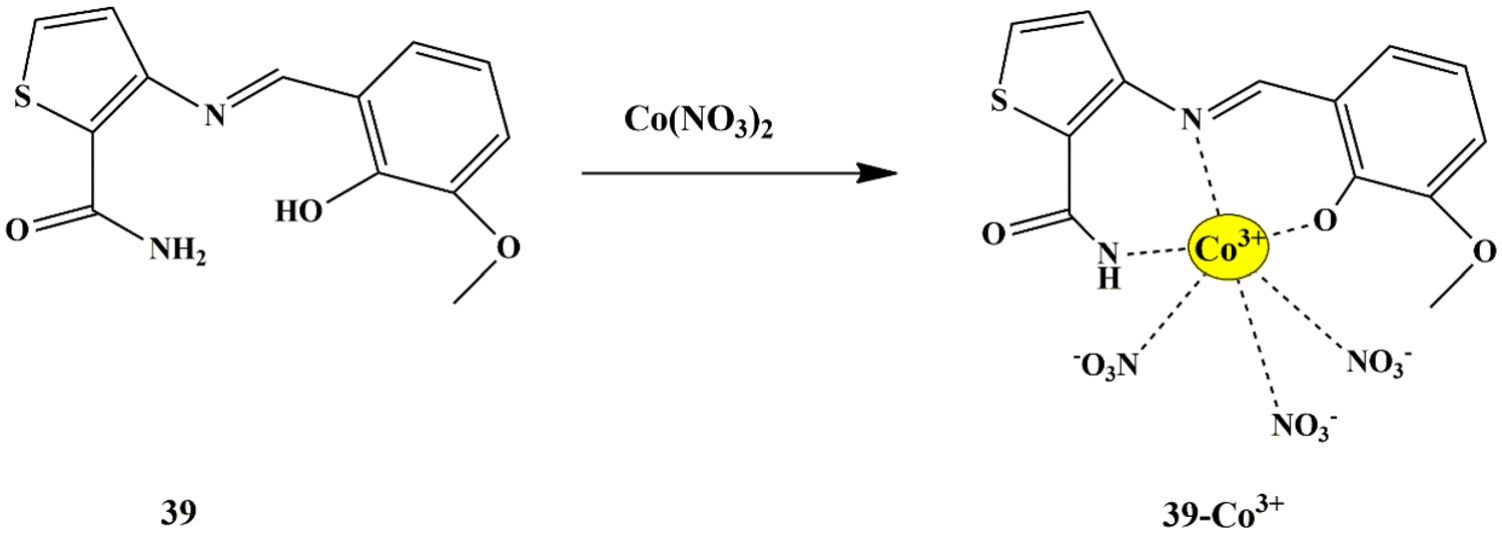
Plausible sensing mechanism of sensor **39** towards Co^3+^

Chang et al. developed a thiophene-carboxamide-based colorimetric chemosensor **39** for detecting Cu^2+^ and Co^2+^ in a near-perfect aqueous solution. The sensor could selectively respond to Cu^2+^ and Co^2+^ ions by a color change from colorless to yellow at 417 nm. Furthermore, the binding properties of the sensor with Cu^2+^ and Co^2+^ ions were investigated through UV-Vis titrations, which revealed that the sensor **39**-cation complex had 1:1 binding stoichiometry. There was no interference from other ions except Cu^2+^, during Co^2+^ detection since Cu^2+^ was tightly bonded to the sensor and this interference could be eliminated by adding excessive F^−^ ions, which could be used to distinguish between probe **39**-Cu^2+^ and probe **39**-Co^2+^ complex by color change. The LOD for Cu^2+^ and Co^2+^ was found to be 0.14 µM and 0.88 µM, respectively. The possible mechanism for detecting Co^3+^ (oxidation of Co^2+^ to Co^3+^ by O_2_ molecules, based on ESI mass result) and Cu^2+^ could be attributed to the ICT and LMCT mechanism **(**Scheme 37**)**. Furthermore, the binding modes of the sensor to Cu^2+^ and Co^2+^ were found to be a 1:1 binding stoichiometry [81].

### Palladium

Palladium is widely used in industrial processes due to its role as a catalyst in many organic synthesis reactions such as Suzuki coupling, Heck reaction, Wacker process. Although the toxicity of palladium is believed to be low, it coordinates with nucleic acids, proteins, and other macromolecules such as VitB_**6**_, thereby impeding many cellular functions if accumulated in the body.

**Scheme 38:**
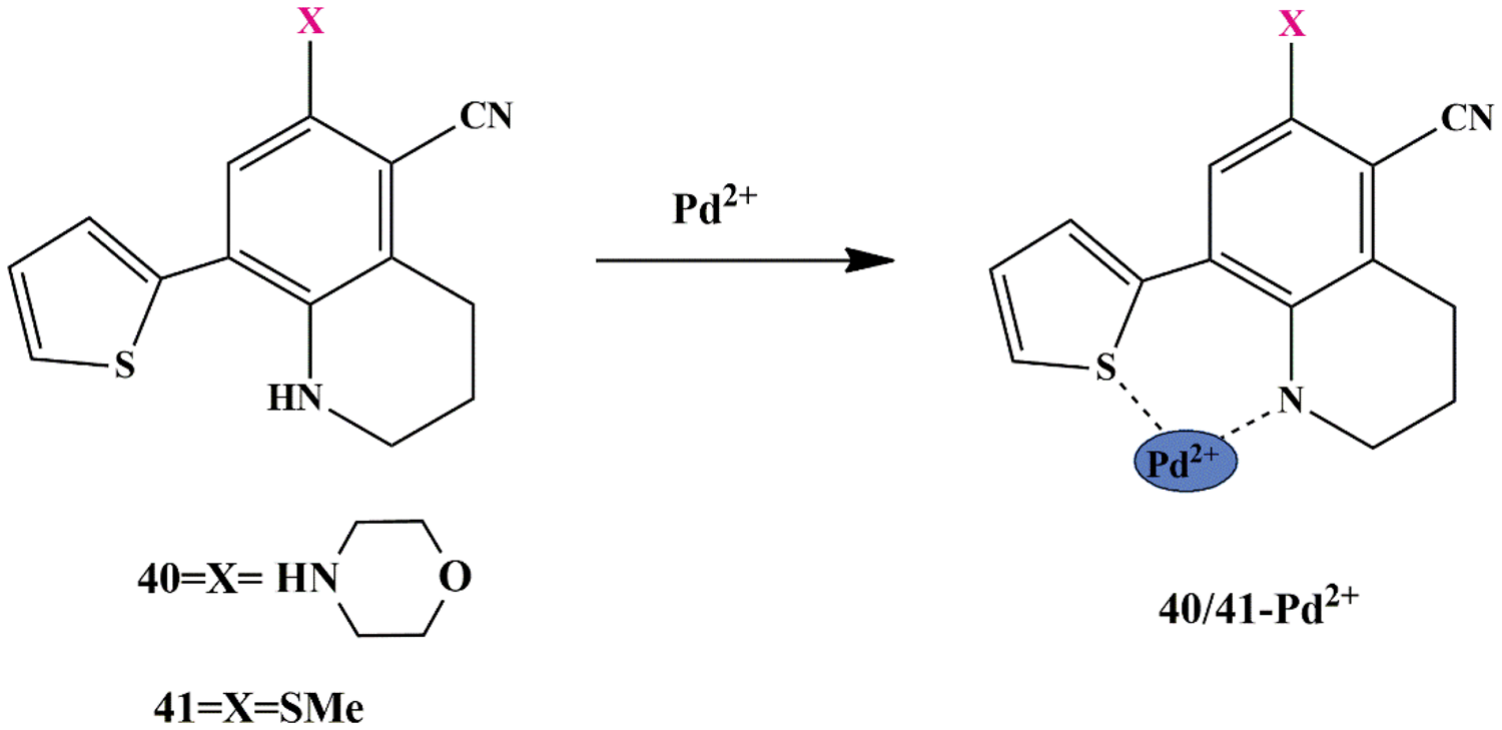
Detection mechanism of **40** and **41** for Pd^2+^ ion

Palladium ion has an open-shell electronic configuration and hence could behave as a fluorescence quencher and considering this aspect, Shally et al. designed thiophene appended tetrahydroquinoline-based ‘turn-off’ chemosensors **40** (with morpholino group) and **41** (with methylthio group). The UV-Vis spectrum shows the absorption peak at 390 nm and 378 nm for free **40** and **41** sensors respectively, but the peak at 390 nm faded and a new peak appeared at 325 nm for **40** whereas, the absorption peak of probe **41** at 378 nm vanished and a new peak was generated at 430 nm, upon the addition of Pd^2+^ ions, as evidenced by the color change to yellow from colorless in probe **41** only. In the presence of Pd^2+^ ions, both the fluorescence emission intensities of both sensors were significantly quenched, with probe **41** experiencing a more substantial quenching compared to **40**. Job’s plot suggested stoichiometry binding of both sensors with Pd^2+^ ion is in 1:1 ratio. Besides, both were found to behave as bidentate ligands [82], by binding through Sulphur of thiophene and hydroquinoline N-atom to Pd^2+^ **(**Scheme 38**)**, as confirmed by HRMS spectra, NMR spectra, and DFT studies. The LOD of probes **40** and **41** was calculated to be 49 × 10^−9^ M and 44 × 10^−9^ M. These chemosensors illustrated better efficiency and promising results with a lower LOD.

It has also been observed that thiophene units as spacers in combination with crown ethers exhibit modulatory sensory behavior to obtain selective cationic (Hg^2+^, Pb^2+^, Cu^2+^, Pd^2+^) chemosensors [83]. One such novel approach was made by Rosa et al. and wherein they synthesized new (oligo) thiophene bipendant-armed ligands and were assessed as chemosensors in the presence of Na^+^, Ag^+^, Pd^2+^ and Hg^2+^ in methane-sulfonic acid by spectrofluorimetric titrations. Compounds bearing bithiophene moiety were found to behave as a palladium chemosensor since enhanced fluorescence intensity was noticed. Subsequent additions of Na^+^ and Pd^2+^ gradually enhanced the emission band intensity, which could be attributed to the fact that complexation of Na^+^ at the crown ether occurs, followed by interaction of Pd^2+^ with sulphur atoms [84] of the bithiophene unit.

## Thiophene Based Chemosensors for the Detection of Anions

Anions comprise an indispensable component of various clinical, industrial processes. Due to their beneficial and detrimental role in the living systems, there is a scope for anion recognition in the future employing fluorescent chemosensors, thereby quantifying and detecting the anions relatively inexpensive and easier. Few receptors assimilated with transition metal ions such as copper, nickel, zinc, and ruthenium normally respond to an analyte by manifesting perceptible color or optical changes through metal-anion interactions in a solution under neutral conditions. Some of the common anions are cyanide (CN^−^), bisulfite (HSO_3_^−^), Fluoride (F^−^), Chloride (Cl^−^), Bromide (Br^−^), Iodide (I^−^), Acetate (AcO^−^), Perchlorate (ClO_4_^−^ ).

### Cyanide

Cyanide ions are one of the most toxic ions because of their excellent binding ability with ferric (Fe^3+^) ions in heme, which obstructs binding with reduced divalent ferrous (Fe^2+^) ion and interrupts the electron transfer biological oxidation, causing internal asphyxia. The brain will be first damaged due to the condition of hypoxia, which consequently leads to respiratory problems and death [85]. Cyanide toxicity also induces convulsions, loss of consciousness. Because of its wide application in diverse sectors, developing a simple, quick method for detecting CN^−^ ions is reasonably necessary.

**Scheme 39:**
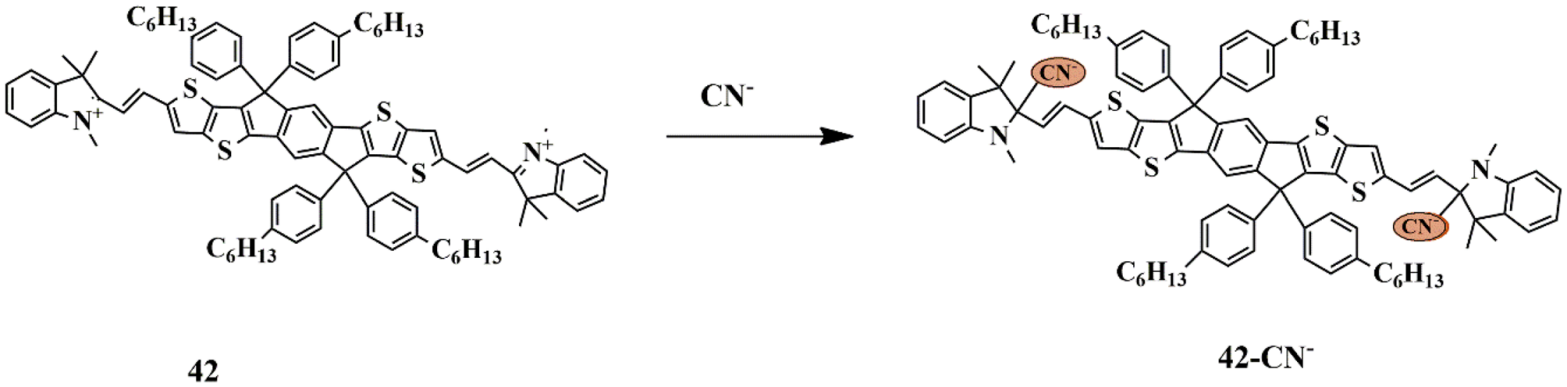
Detection mechanism of sensor **42** towards CN^−^

Dan Wen synthesized a novel chemosensor **42** for quick detection of cyanide (CN^−^) ions. UV-Vis spectral studies confirmed that the absorbance band of 625 nm, exhibited by the sensor in CH_3_CN/H_2_O (v/v 1:1) gradually reduced, and new absorbance bands at 430 nm and 458 nm occurred, with a color change from blue to yellow only for CN^−^ ions, which is attributable to the blocking of ICT between the hemicyanin moiety and sensor **42** due to the nucleophilic attack of CN^─^ to indolium group **(**Scheme 39**)**. Moreover, investigating the practical applications of the probe, it was observed that sensor **42** filter paper could detect CN^−^, which is evident by the color change from blue to yellow and the LOD was noted to be 0.89 µM [86], implying that the probe could be implemented for the detection of CN^−^ ions.

**Scheme 40:**
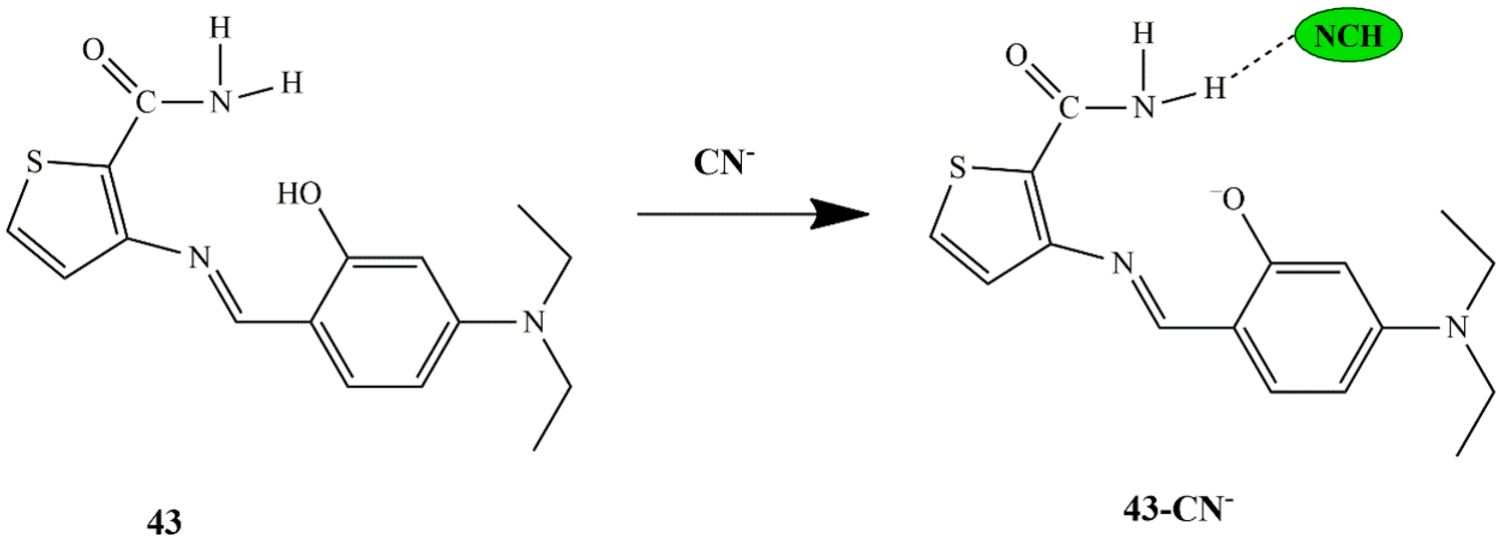
Recognition mechanism of probe **43** for CN^−^ ions

Min et al. developed a new probe **43** [((E)-3-((4-(diethylamino)-2-hydroxybenzylidene) amino)-2,3-dihydrothiophene-2-carboxamide)] for CN^−^ detection. UV-Vis titrations studies revealed that the peak at 430 nm decreased, with the occurrence of two new bands at 320 nm and 470 nm upon the addition of CN^−^ ions to the probe solution. It was able to selectively sense CN^−^ ions through fluorescence enhancement. ESI-MS data and job plots were analyzed, and the binding mode was in 1:1 stoichiometry. The LOD for CN^−^ was 44.6 µM, and the detection process was proposed through H-NMR titrations, which showed that deprotonation of the probe could result in the ICT suppression, thereby inducing fluorescence ‘turn-on’ of probe **43**-H^+^ **(**Scheme 40**)**. From the results, it was then confirmed that the probe [87] could be utilized for the selective detection of CN^−^ by the fluorescent turn-on method in aqueous systems.

**Scheme 41:**
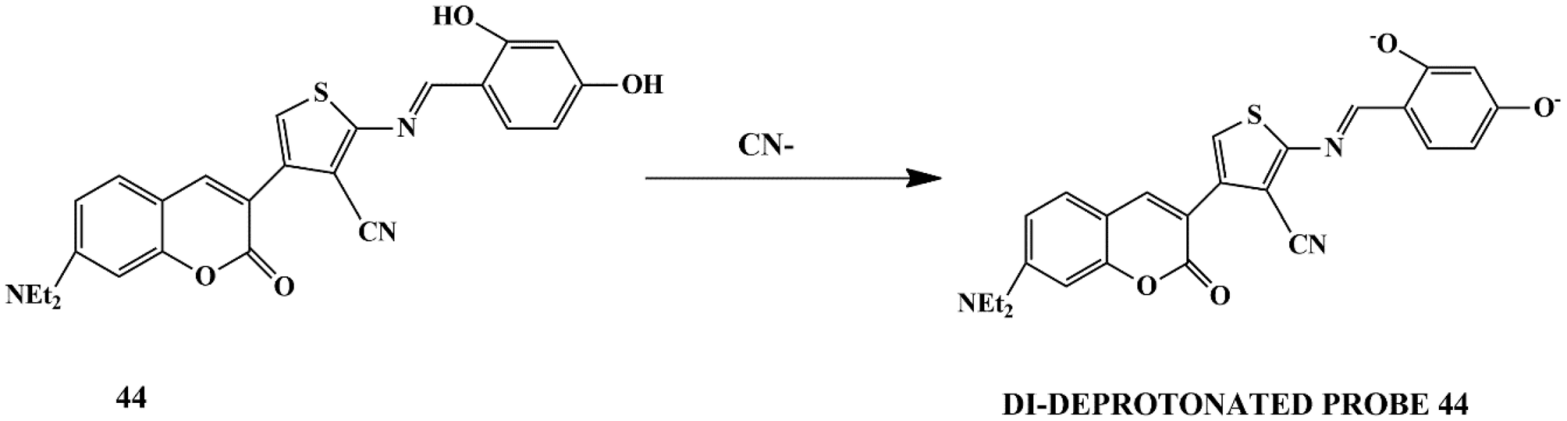
Turn-on Di-deprotonated chemosensor **44**

Various chemosensors have been established for the CN^−^ detection that is soluble in aqueous media to mimic the real environment. But, developing a chemosensor for the selective detection of CN^−^ ions in the presence of highly competitive ions such as F^−^, I^−^ remains a challenge. In this regard, Meryem and his colleagues synthesized a coumarin-thiophene-based schiff base chemosensor **44**. The anion sensing properties were carried out in DMSO and water and further compared. The chemosensor in DMSO with only F^−^, CN^−^ and AcO^−^ ions showed few spectral changes in the UV-Vis titration spectra, whereas colorimetric sensing showed that only F^−^, AcO^−^, CN^−^, H_3_PO_4_^−^ ions exhibited a color change of the solution from yellow to orange. However, chemosensor **44** in DMSO/H_2_O (v/v 1:9) exhibited selectivity only towards CN^−^ ions with a 1:1 binding stoichiometric ratio, which was evident by the increased fluorescence intensity in the fluorescence spectra. H-NMR spectroscopy studies, corroborated by DFT and TD-DFT results confirmed the mechanism of the sensing process, which displayed the deprotonation [88] of hydroxyl moiety on the phenyl part of the schiff base upon reaction with CN^−^ ions **(**Scheme 41**)**. Nevertheless, the reversibility studies were carried out in TFA, and the sensing reaction was found to be reversible. The detection limit for CN^−^ ions in an aqueous solution was 0.32 µM, which was relatively lower than in organic media (LOD=1.69 µM). The results indicated that the chemosensor could quickly detect CN^−^ via a di-deprotonation and ICT mechanism in the aqueous solution. Furthermore, the possibility of chemosensor being used in practical applications was evaluated using tap water, and it was observed that chemosensor **44** showed high-intensity green fluorescence on reaction with CN^−^ ions, thereby paving the way for real-time monitoring of anions in aqueous and environmental samples. Moreover, the probe was also explored to be DNA-detection agent due to the intercalation of **44** into duplex DNA.

**Scheme 42:**
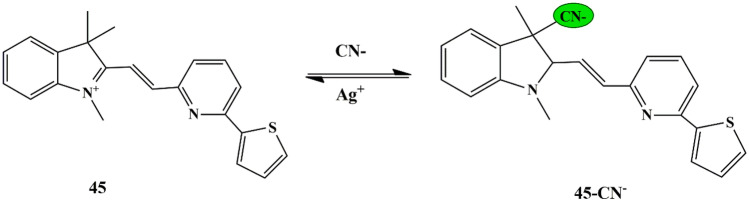
Proposed sensing mechanism of **45** for CN^−^ ions

A ratiometric probe was reported by Ajit Kumar et al. wherein an indole conjugated thiophene-pyridyl-based probe **45** was employed for CN^−^ detection. Fluorescence Titration of the probe with CN^−^ exhibited a blue shift along with fluorescence quenching of the band at 619 nm and the emergence of a new band at 504 nm. The absorbance titrations exhibited spectral changes, with a noticeable color change from dark brown to colorless in the presence of CN^−^ ions and could be assigned to the blocking of ICT due to the adduct formed by the nucleophilic addition of CN^−^ to indolyl cation of probe **45** (Scheme 42**)**; confirmed by TD-DFT calculations [89] and HRMS spectra, with 1:1 stoichiometric ratio between the probe and CN^−^ ions. Furthermore, fluorescence competitive titrations were carried out, which revealed that the probe was highly sensitive towards CN^−^ ions. For the practical application of the probe, the test paper strips based on probe **45** were prepared, which could detect CN^−^ with an immediate color change. It could also be used for biomedical applications for sensing CN^−^ at significantly lower concentrations.

**Scheme 43:**
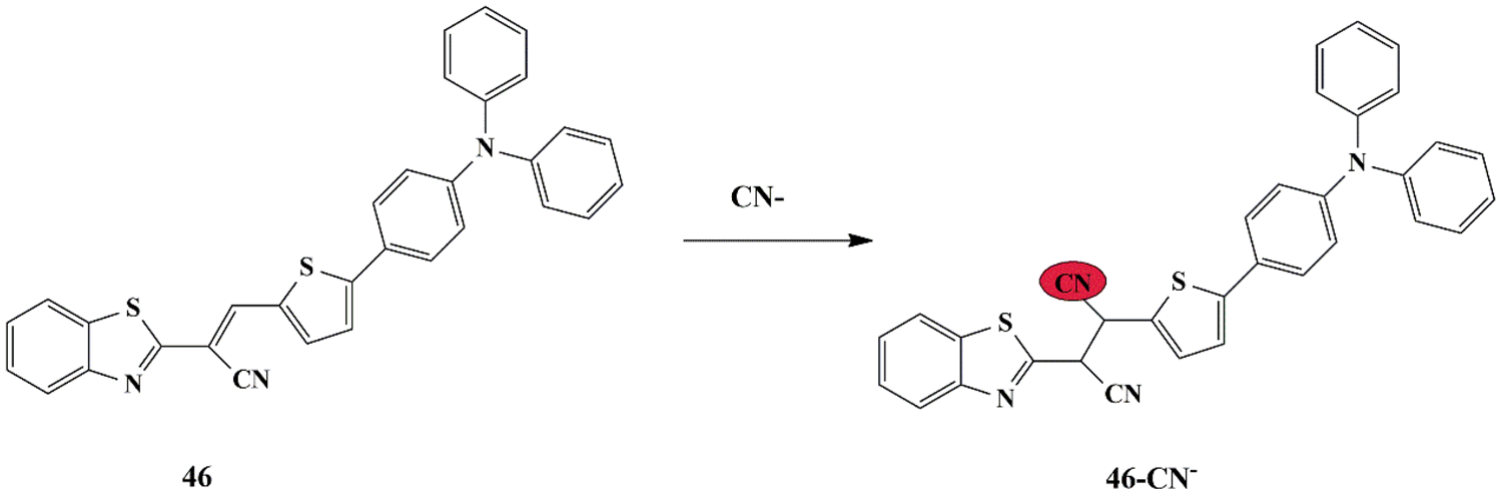
Plausible sensing mechanism of probe **46** for CN^−^ ions

Yan Zhao synthesized an off-on type fluorescent chemosensor **46**, which has a triphenyl amine bearing thiophene derivative of benzothiazole as a CN^−^ sensor. The free probe showed a broad absorbance band at 545 nm in PBS buffer in the UV-Vis absorption spectra, which decreased along with the emergence of a new band at 340 nm upon addition of CN^−^. It was also seen that strong ‘turn-on’ fluorescence enhancement is notable only for the CN^−^ ions, without any interference by other anions. The Job’s plot indicated a 1:1 binding mode between probe and CN^−^ and the LOD for the CN^−^ ions is 4.24 × 10^− 8^ M. The fluorescence enhancement is ascribed to the interruption of ICT of the probe [90] due to the attack of CN^−^ ions at the cyano vinyl group **(**Scheme 43**)**, as corroborated by NMR studies and DFT calculations. Furthermore, probe **46** could be used in real-time monitoring of CN^−^ ions by test strips in aqueous solution, evidenced by the color change from mild violet to light yellow-green fluorescence, under UV light only in the presence of CN^−^ and could also be implemented for fluorescence imaging of live cells.

**Scheme 44:**
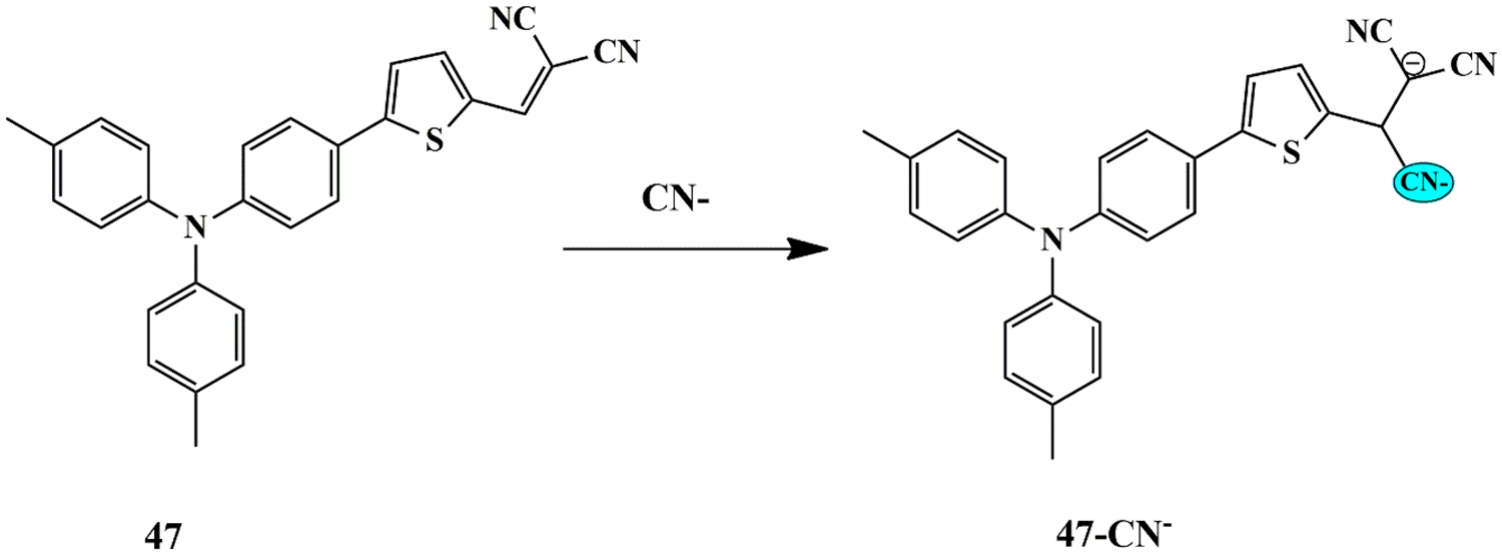
Proposed sensing mechanism of probe **47** for CN^−^ ions

Numerous sensing mechanisms for CN^−^ have been proposed for fluorescent chemosensors via different mechanisms, with nucleophilic addition being the highly selective mechanism to sense CN^−^ ions. Utilizing this sensing mechanism, Zi Hua et al. synthesized and evaluated the sensing properties of a novel triphenylamine thiophene-based (with dicyanovinyl moieties) probe **47**. The free probe exhibited pink non-fluorescent color due to the ICT mechanism [91], which further faded in the presence of CN^−^ ions, accompanied by a fluorescence emission enhancement at 480 nm. Furthermore, the UV-Vis absorbance spectra revealed that only for CN^−^, spectral changes and color change of light pink to colorless under naked-eye and bright blue under UV light was observed. The sensing mechanism is attributable to the nucleophilic addition reaction to form probe [**47**-CN^−^] adduct **(**Scheme 44**)**. Moreover, a low LOD was determined to be 51 nM, which indicates that the probe could be employed to detect low levels of CN^−^ in drinking water, which also met the requirements of WHO for CN^−^ detection. Additionally, probe **47** could be employed in bio-imaging to detect CN^−^ ions in HeLa cells in a concentration-dependent manner, with lower cytotoxicity and good cell permeability.

**Scheme 45:**
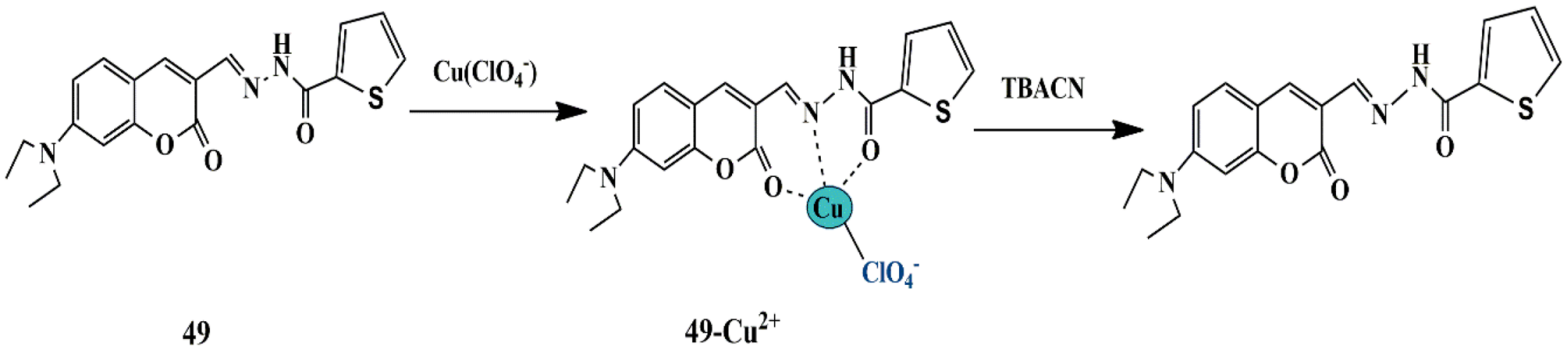
Suggested detection mechanism of probe **49** for CN^−^ ions

In recent years, chemosensors relying on copper ion displacement for CN^−^ detection have achieved significant attention. Successful examples are the coumarin-based schiff bases **48** and **49** chemosensors possessing furan and thiophene as the side groups respectively, developed by Kangnan and group. Both the chemosensors showed high affinity towards Cu^2+^ ions, which was evident by the spectral changes and fluorescence quenching, indicating the complex formation of **48/49**-Cu^2+^ and PET processes, respectively. Moreover, probe **48/49**-Cu^2+^ complex could detect CN^−^ ions, with obvious and immediate color change from red to yellow-green, which is assigned to the strong binding ability of Cu^2+^ towards CN^−^ ions, consequently resulting in the Cu^2+^ removal from the complex and recovery of the probes, thereby showing the copper displacement mechanism [92] **(**Scheme 45**)**. The detection limits of probe **48**-Cu^2+^ and **49**-Cu^2+^ for CN^−^ were 0.019 µM and 0.02 µM, respectively. The reversibility of these chemosensors was evaluated and was found to exhibit a ‘turn on-off’ mechanism with the introduction of CN^−^. Therefore, both probes could be re-used several times for CN^−^ detection.

## Fluoride

Fluoride is naturally present in the human body in bones and teeth and drinking water is the primary source of fluoride ions. However, excess fluoride is hazardous and can be detrimental to life by causing severe dental fluorosis or skeletal fluorosis [93]. It is highly beneficial to develop a highly effective chemosensor that can selectively detect F^−^ ions with the naked eye response.

**Scheme 46:**
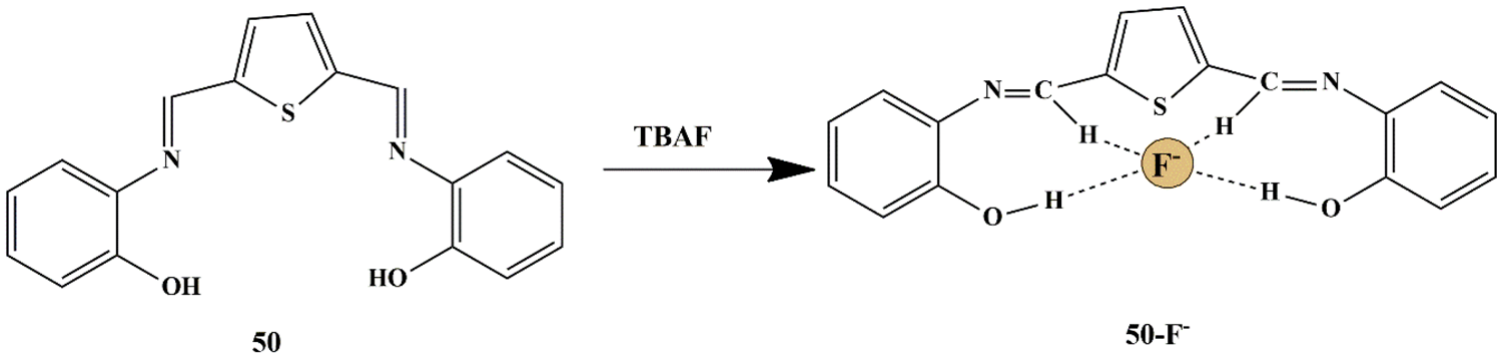
Probable sensing mechanism of **50** towards F^−^ ions

One successful approach was made by Renuga et al. who synthesized fluorescent receptor **50** [2,20 -(1E,10 E)-(thiophene-2,5-diylbis(methan-1-yl-1-ylidene)] for selective detection of F^−^ ions in the presence of competing anions in CH_3_CN. A color change was observed from fluorescent green to orange in the presence of F^−^ ions only, which could be attributed to the H-bonding between the OH^−^ group of receptor **50** and F^−^ ions **(**Scheme 46**)**. Upon using spectrophotometric techniques, a new red-shifted absorption band centered at 520 nm was observed for an increase in F^−^ ions [94], which could be explained by the Internal Charge Transfer mechanism. The binding constant was calculated as 1.3 × 10^3^ and the binding between the receptor and anion was in 1:1 binding stoichiometry. Further, fluorescence spectra displayed that the receptor produced a strong emission band at 510 nm and 545 nm because of its strong fluorescent nature, manifesting that the chemosensor can be utilized to detect F^−^ in the presence of competing anions.

**Scheme 47:**
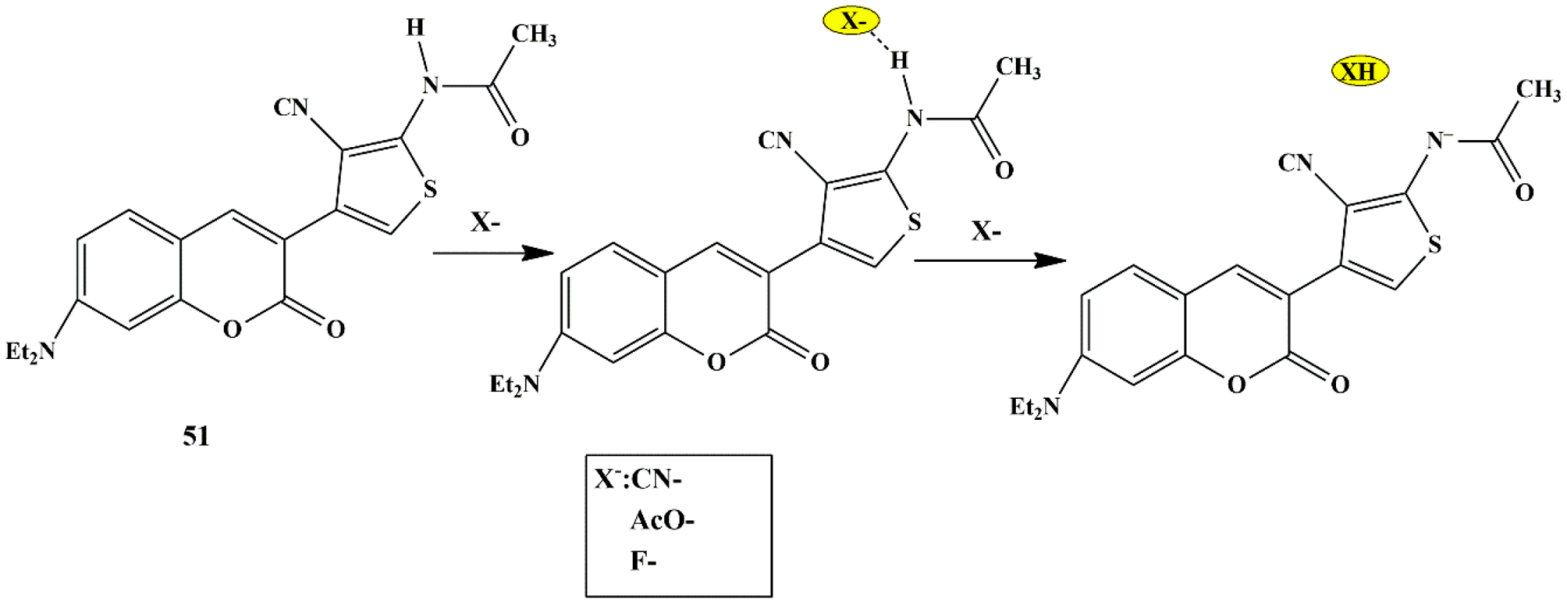
A suggested mechanism for the detection of anions by **51**

Few Thiophene derivatives have also been reported for the sensing of anions, due to their optical property. For instance, Ufuk Yanar et al. synthesized novel coumarin-acetamido thiophene-based colorimetric and fluorometric chemosensor **51** for ratiometric sensing of anions. The chemosensor exhibited visible colorimetric changes (color change from light yellow to deep yellow) and emission quenching in the presence of CN^−^, F^−^ and AcO^−^ in DMSO. Nevertheless, the probe was more sensitive to CN^−^ than F^−^ and AcO^−^ at the stoichiometric binding ratio of 1:2:5. Molecular orbital analysis was studied, which explained that the fluorescent property and the quenching property of probe **51** were because of the π — π*‎ transition on coumarin and ‎ charge transfer transition from donor to acceptor unit, respectively. The mechanism of fluorescence quenching was due to ICT [95] **(**Scheme 47**)**, which was supported by Computational studies.

An innovative approach was made by Neeraj et al. who attempted and succeeded in controlling the selectivity of the sensor by modification of the side groups of the hydrazone Schiff bases of sensors, which were derived from 1,8-naphthalimide and substituted furan/ thiophene ring systems. All four sensors responded selectively to F^−^ and CN^−^ ions with an abrupt color change from yellow to blue. The fluorescent emission intensity was quenched significantly with negligible interferences from other tested anions. It was concluded that a stoichiometric binding ratio of 2:1 exists between probes **52/53** (having thiophene/ furan rings respectively) and F^−^ ions, while 1:1 binding mode exists between probes **54/55** (having furan with -NO_2_ and furan with -CH_3_ group respectively) with F^−^ and CN^−^ respectively. Sensors **52** and **53** were found to exhibit the lowest detection limit of 0.04 ppm and 0.07 ppm for F^−^ and CN^−^ ions. Moreover, the mechanism of detection was due to the Hydrogen bonding succeeded by deprotonation, resulting in the ICT process. In addition, discriminative detection of anions was performed with Cu^2+^ ions, and it was found that sensors **54** and **55** exhibited selective recognition of F^−^ over CN^−^ ions; which was evident by spectral changes and the ‘naked eye’ color change from bright yellow to dull yellow, whereas yellow to green for sensor **54** and **55** respectively [96]. Only sensors **54** and **55** could effectively detect both F^−^ and CN^−^, whereas **52/53** was less effective in CN^−^ detection because of the loss of CN^−^ fluorescence by the addition of other anions. The applicability of sensors was examined by test strip experiments, which exhibited significant color changes upon the detection of F^−^/CN^−^ ions.

**Scheme 48:**
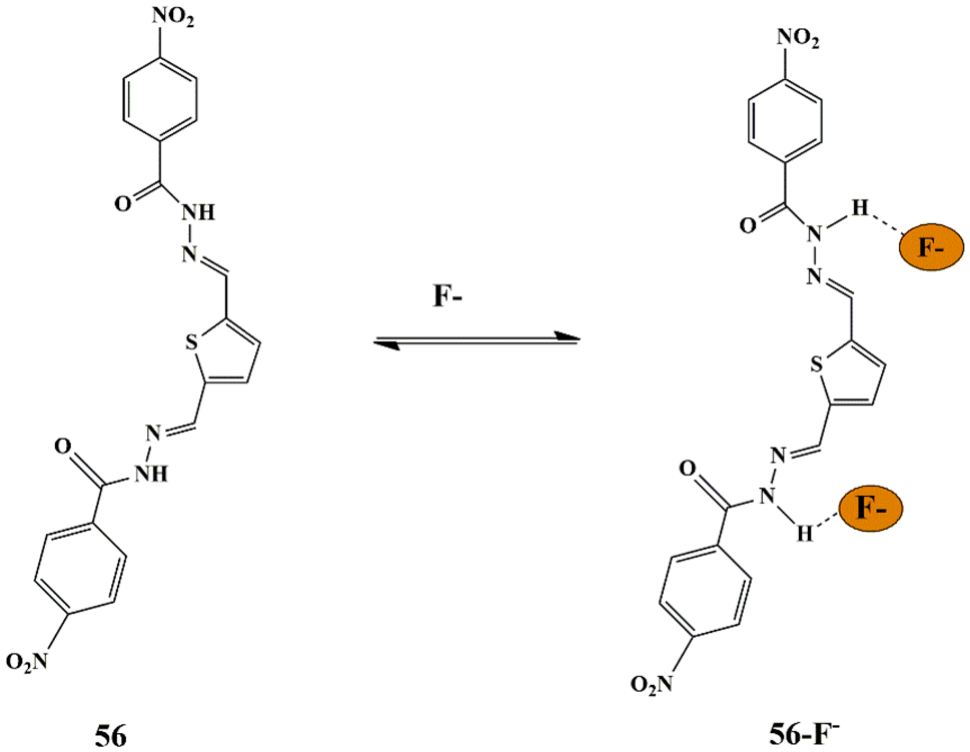
Proposed sensing mechanism of probe **56** for F^−^ions

Following the same design guide, Siddarth et al. synthesized a new bis Schiff base **56** with dual –NH functionality for F^−^ detection. The chromogenic response of the probe in CH_3_CN solution was found to be selective to F^−^ over other anion analytes, which was evident by the naked-eye color change from colorless to red. The UV-Vis absorbance spectra of the free probe exhibited two peaks at 260 nm and 380 nm, which were then subjected to red-shift along with the occurrence of a new band at 478 upon the addition of F^−^ ions. The sensing mechanism could be assigned to the Hydrogen bonding and deprotonation [97] between probe **56** and F^−^ ions **(**Scheme 48**)** with 1:2 stoichiometry, corroborated by H-NMR studies and DFT studies. The LOD was calculated to be 6.9 × 10^−7^ M, with a response time of 20 s, thereby meeting the requirements of real-time monitoring of F^−^ ions.

**Scheme 49:**
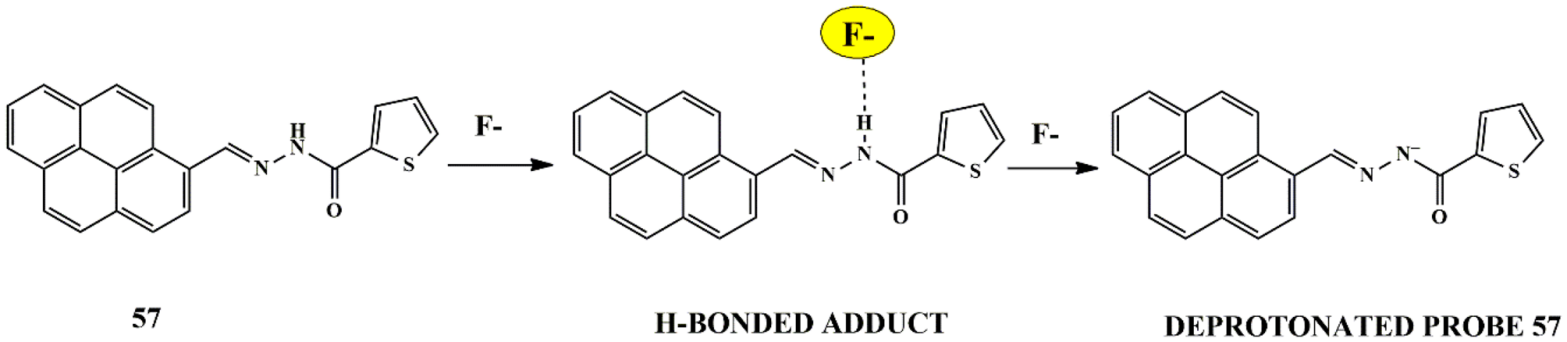
Proposed detection mechanism of **57** for F^−^ ions

Recently, various AIEE based fluorescent dyes based have been synthesized, since they could be an efficient optical tool exhibiting distinctive properties with promising results, and therefore could be employed in bioimaging, organic light-emitting devices, and chemosensors. One such approach was made by Pranjalee et al. who synthesized a pyrene-thiophene-based probe **57** based on the AIEE property for F^−^ detection. The probe exhibited a new peak at 450 nm with a hypochromic shift in the UV spectra, along with the visible color change from colorless to bright yellow upon interaction with F^−^ ions, without the perturbation of detection by other anions. Furthermore, the UV-titration experiments revealed a new peak at 450 nm, which indicates H-bonding between F^−^ and probe **57**, thereby inducing the deprotonation of NH group with increasing F^−^ ion concentration, resulting in push-pull effect, further confirmed by NMR and DFT studies. The binding mode was found to be in 1:1 stoichiometry ratio, and the LOD was estimated to be 2.02 × 10^−7^ M. The AIEE property of probe **57** was evaluated in the CH_3_CN/H_2_O mixture and could be attributed to the hindered intramolecular rotation and the formation of J-type aggregates [98] **(**Scheme 49**)** which suppresses the non-radiative decay of excited state. The potentiality of the sensor to sense F^−^ was also evaluated by test strip experiments. The sensor displayed a visible color change of strips from colorless to yellow (under UV light) upon detection of F^−^, thereby signifying its potential application for F^−^ recognition.

Presumably, Thienothiophene (TTs) moiety has rarely been employed as a donor group in Donor-Acceptor systems possessing excellent sensing properties. Thienothiophenes is one of the structurally derived thiophene isomers, which has an electron-rich conjugated structure, and is integrated with polymers and other small compounds to enhance their photophysical and electronic properties, thereby upgrading the device performance. In the past few years, thiophenes bearing electron-deficient borane in the main and side chains of oligomers, and polymers have gained significant importance, and representative examples are the chemosensors **58** and **59**, possessing conjugated thieno[3,2-b]thiophene and cross-conjugated thieno[2,3-b]thiophene, developed by Gulsen et al. for F^−^ detection. The vital photophysical behaviors of both sensors were due to the ICT mechanism between thienothiophenes and two bulky dimesitylboron units. Sensor **58** exhibited exceptional bathochromic shifts in both absorption and fluorescence spectra as compared to sensor **59**. Furthermore, both sensors showed positive emission solvatochromism with an increase in solvent polarity owing to their quadrupolar structures. Among the two sensors, **58** was found to be an efficient blue emitter. The characteristic spectral changes in the absorption and emission spectra and noticeable color change were observed upon binding of only Lewis base F^−^ to the Lewis acid organoboron group [99]. The results concluded that probe **59** responded through turn-on and probe **59** responded through ‘turn-off’ response, thereby possessing high sensitivity and selectivity toward F^−^ anions without interference from other anions. The high quantum yield, good solvatochromic behavior, and intense interaction with F^−^ anions entitle these compounds to be used as potential luminescent chemosensors.

One more similar approach was made by the same group of researchers Gulsen et al. who synthesized four novel Organoboron copolymers consisting of thienothiophene and selenothiophene analogs with thiophene π-spacer. The sensors **61** and **63** having cross-conjugated selenopheno[2,3-*b*]thiophene and thieno[2,3-*b*]-thiophene, respectively exhibited weak spectral changes due to the poor electronic couplings of cross-conjugated donors and mesityl boron acceptor in the polymer chain. In contrast, probes **60** and **62** exhibited strong spectral changes in the UV-Vis absorption spectra. Furthermore, it was found that probe **60** exhibited the lowest bandgap as compared to the other copolymers, and it also exhibited the largest stokes shift [100]. All sensors exhibited a turn-off response accompanied by a color change from yellow to colorless, among which probe **63** showed selective sensitivity of F^−^ because of the presence of less polarizable sulfur atom of thiophene compared to selenium and its cross-conjugated structure. Therefore, these copolymers could be employed as promising candidates for F^−^ detection.

### Iodide

Iodine is a trace element, which mainly exists in the form of iodide in the human body, and is involved in biological functions, for instance, thyroid gland function. Apart from that, elemental iodine has been exploited in diverse applications, for instance, in the manufacturing of dyes, synthesis of some organic chemicals, and medicine. On that account, it is crucial to develop sensitive chemosensors to detect I^−^ in food, pharmaceutical products, and biological samples.

Kannikanti et al. synthesized a turn-off fluorescent chemosensor **64** [2,5-bis(4-phenylquinazolin-2-yl) thiophene] for the sensing and detection of I^−^ ions. The recognition of probe towards anions was studied by absorption and emission spectra, which revealed that the spectra remained unaltered until I^−^ ion was added to the CH_3_CN solution of the probe. The absorption of the probe was observed at 291 nm and 362 nm with a color change from colorless to yellow. Correspondingly, the fluorescence emission intensity of the probe quenches upon interaction with I^−^ ion with 2:1 stoichiometry. The LOD for I^−^ was found to be 1.7 × 10^−7^ M [73]. Furthermore, to evaluate its potentiality in practical applications, probe **64** was utilized to construct a field test kit for the qualitative analysis of I^−^ in aqueous samples.

**Scheme 50:**
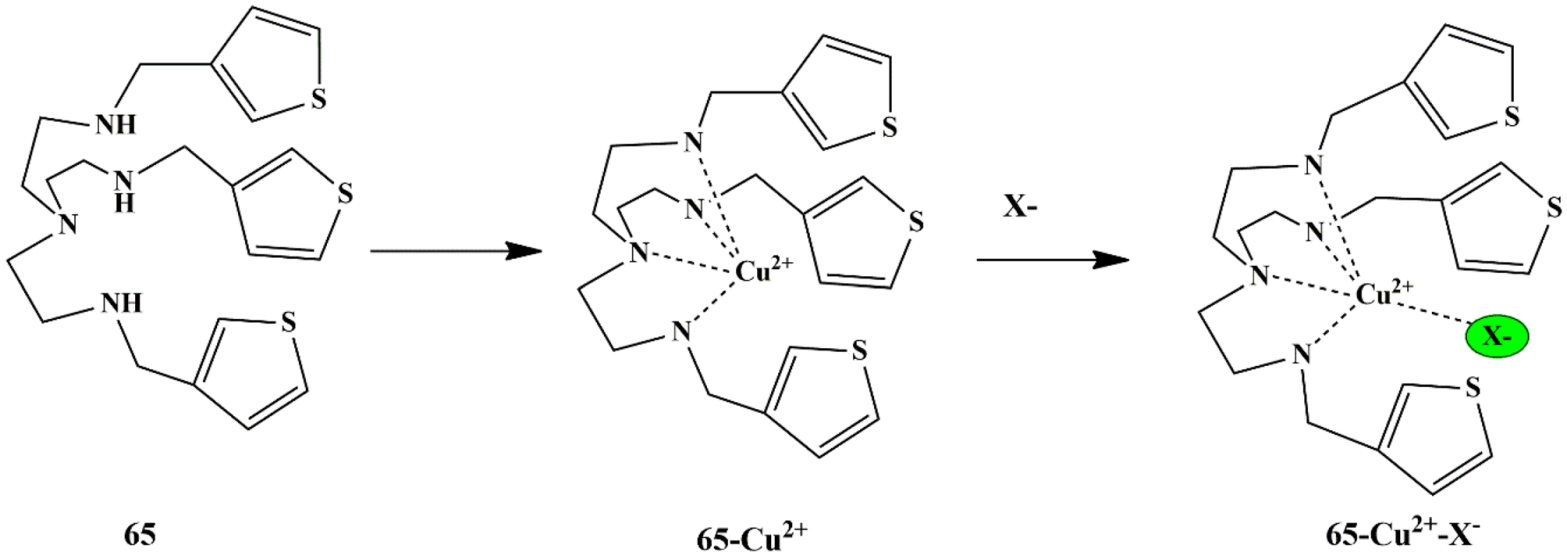
Proposed halide bound complex

Syed et al. synthesized a novel thiophene-based tripodal copper (II) complex receptor **65** to detect halides. It showed distinct color changes in CH_3_CN solution from blue to aqua, lime, turquoise, and greenish-yellow in the presence of F^−^, Cl^−^, Br^−^, I^−^ ions, respectively. The binding properties of the receptor were evaluated by UV-Vis titration experiments, which exhibited a 1:1 stoichiometric complex of receptor **65** with each halide. Furthermore, it was observed that the receptor responded distinctly to different halides concerning the intensity and absorption maximum, due to axial ligation of the respective halide to the coordinatively unsaturated copper center, resulting in the halide complex formation **(**Scheme 50**)**. The order of receptor **65** bindings to the halides was observed in the order of F^−^>Cl^−^>Br^−^>I^−^, which is ascribed to the basicity of the respective halide. Along with this, the effect of solvent polarity on receptor was studied by using CH_3_CN/H_2_O mixture in addition to CH_3_CN, and it was found that the receptor-halide complex showed a weaker binding trend compared to the observed binding trend [101] in pure CH_3_CN, because of increasing solvent polarity.

Conjugated polyelectrolytes have acquired significant attention for many years because of their optical properties and potential applications in optical sensors and biological imaging. They exhibit outstanding characteristics, for instance, their energy transfer which is quite useful as biomarkers by fluorescence quenching and FRET mechanism, high sensitivity, and selectivity by electrostatic binding to target analytes. Moreover, diblocked copolymers of polythiophenes (PT) and Polyflourene (PF) are valuable, versatile materials. One such representative is the conjugated AB diblock copolymer **66** [poly[9,9-bis(2-ethylhexyl)fluorene]-b-poly[3-(6-trimethylammoniumhexyl) thiophene]bromide] designed by Sofia et al. for the selective sensing of anions. The absorbance spectrum of the free sensor exhibited structured emission at 400-500 nm and 520-500 nm due to the PF and PT units, respectively, along with distinct fluorescence emission resulting from incomplete energy transfer between the PF and PT units [102]. The halide sensing of probe **66** was evident by the decrease in fluorescence quenching of the PT unit in the presence of halides, clearly following the quenching order of I^−^> Br^−^> Cl^−^, which could be attributed to heavy atom effects and electron transfer. Nevertheless, the sensor could also be employed for DNA quantification by the formation of nanostructured networks.

### Bisulphite

Bisulfites have various applications in wastewater treatment, in the prevention of corrosion, and in DNA sequencing. They also play an essential role in pharmaceuticals, food, and beverage industries since HSO_3_^−^ can hinder oxidation, microbial and enzymatic reactions. However, anthropogenic activities ensue a substantial input to the atmospheric burden of sulfur compounds regionally and globally, which could be threatening to human health and the environment. Sulfur dioxide (SO_2_) is one such primary air contaminant. When inhaled can be converted into bisulfites and sulfites, thereby causing asthmatic attacks, negatively influencing cardiovascular diseases and lung cancer allergic reactions if inhaled excessively. Therefore, there is a high demand for the development of chemosensors for HSO_3_^−^ detection.

**Scheme 51:**
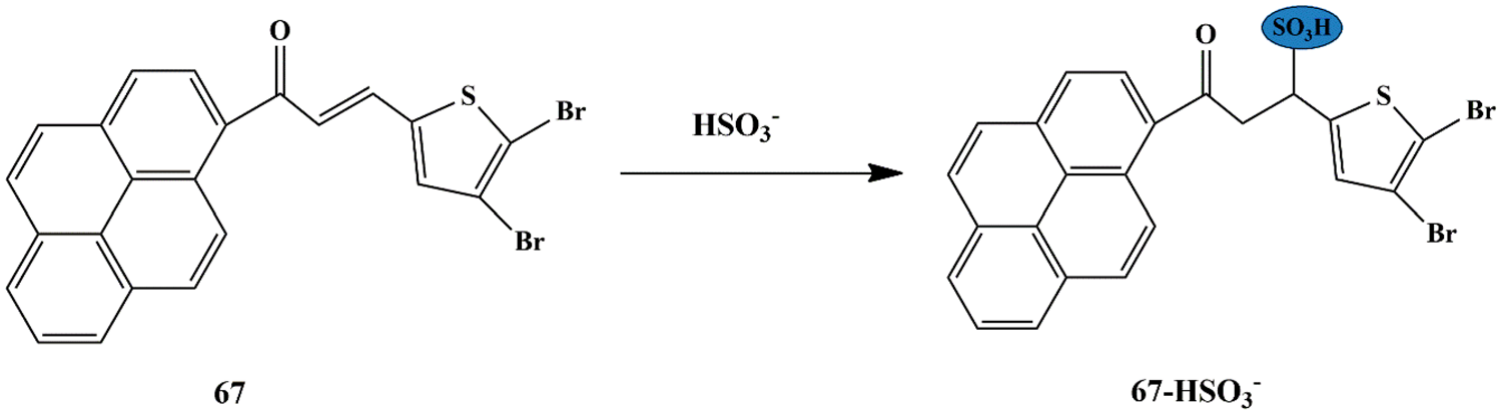
Possible mechanism of probe **67** binding to Bisulphite

Considering the above issues, Jianbin et al. synthesized a Thiophene attached pyrene-based probe **67** and investigated its sensing properties towards HSO_3_^−^ ions. From the absorption and Fluorescence spectra in H_2_O/ethanol solution (v/v 9:1), it was observed that the absorption peak of the probe at 332 & 411 nm reduced gradually, while the fluorescence intensity at 530 nm reduced along with the simultaneous emergence of a new band at 420 nm upon the addition of HSO_3_^−^ ions. Furthermore, the fluorescence spectra showed that the tested anions showed negligible fluorescence intensity except for HSO_3_^−^, which displayed a dramatically enhanced fluorescence intensity of the probe, and this could be attributed to the Michael addition reaction **(**Scheme 51**)** between probe **67** and HSO_3_^−^, confirmed by HRMS spectra.

To evaluate the potentiality of the probe in biological applications, fluorescence imaging was accomplished in A549 cells, *C. elegans*, and zebrafish, which displayed a decrease of yellow fluorescence emission by the free probe and enhancement of blue fluorescence emission upon the incubation of probe **67** with HSO_3_^−^ in the cells, which indicated that the probe possessed a good cell membrane permeability [103] and could be employed in the detection of exogenous and endogenous HSO_3_^−^ ions in vivo.

**Scheme 52:**
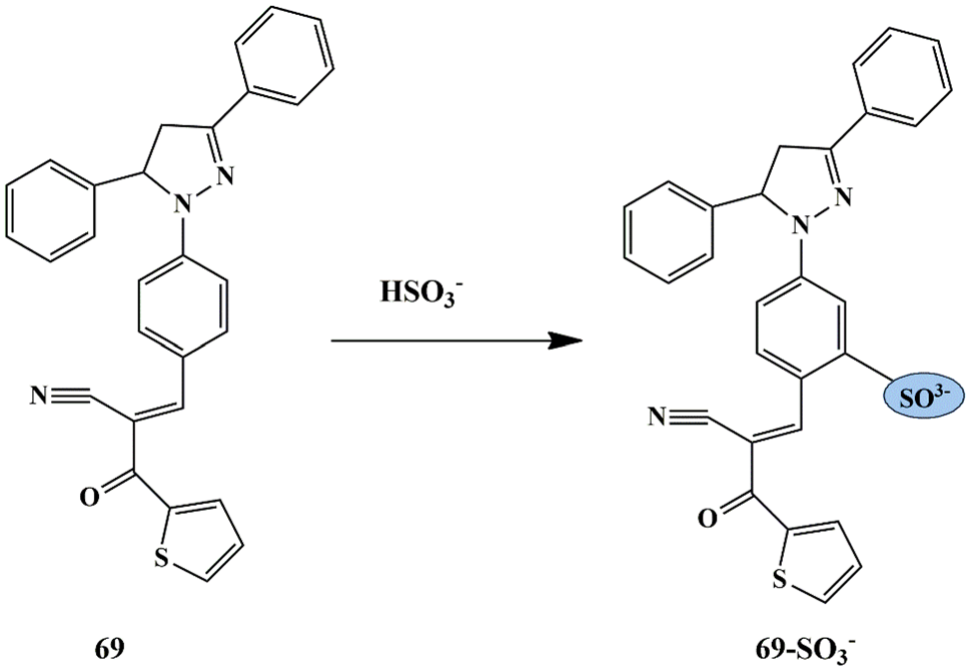
Suggested mechanism of probe **69** binding to Bisulphite

Tomascz et al. synthesized two Off-On pyrazoline-based chemosensors **68** and **69** having two different end groups being phenyl and thiophene moieties respectively, which were capable of detecting sulphite anions. Both the probes exhibited weak ICT fluorescence, which is obstructed after the nucleophilic sulfite ion attachment to the α, β-unsaturated ketone part of the probe, confirmed by NMR and DFT studies. Subsequently, a blue shift of the absorption and emission spectra of the probes was seen, corresponding to a visible naked eye color change from orange to colorless. Among the two probes, **69** was found to be effective in the selective detection of HSO_3_^−^ by significant quenching due to the presence of the thiophene group **(**Scheme 52**)**. Nevertheless, the practical application of the probes was illustrated by utilizing a paper test strip method for HSO_3_^−^ detection. It was observed that the paper discolored from orange or yellow to blue [104], confirming its potentiality to detect HSO_3_^−^ in water samples. In this study, a simple replacement of end groups by thiophene surpassed the response time by 1.2 times and the detection limit by 1.5 times.

**Scheme 53:**
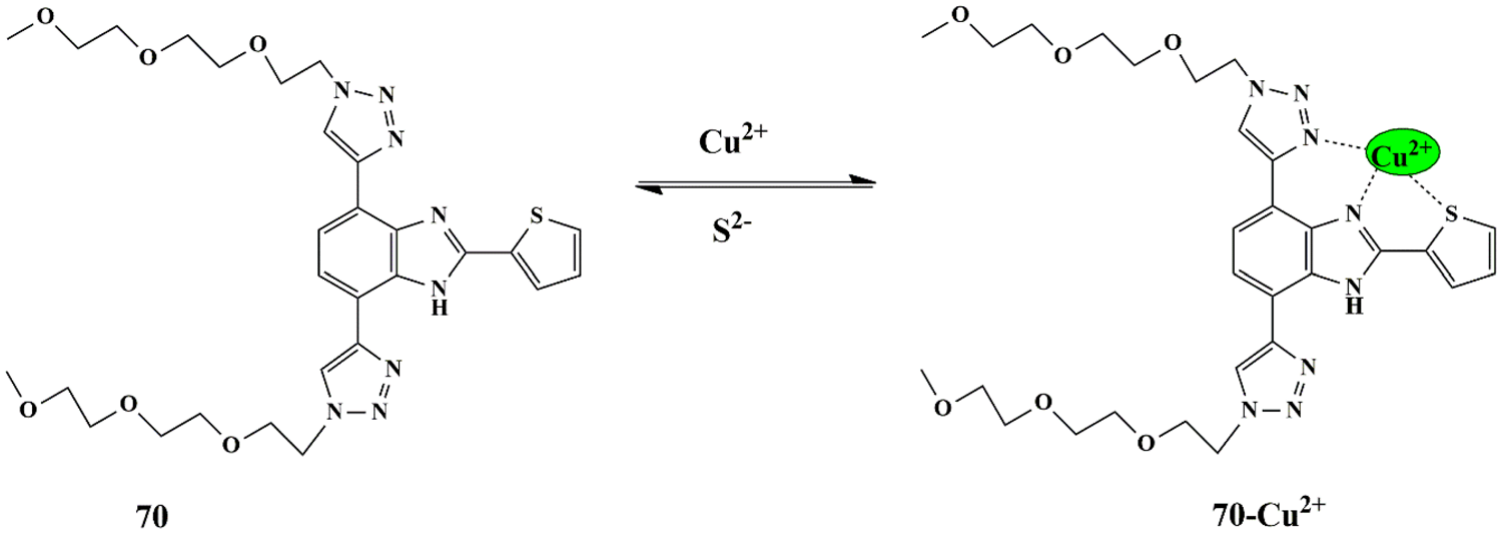
Plausible detection mechanism of Probe **70**−Cu^2+^ ensemble for S^2−^ ions

Encouraged by the need for water-soluble fluorescent chemosensors, Chak Shing et al. synthesized a Thiophene-benzimidazole-based chemosensor **70** to detect S^2−^ ions in Water. The strong blue fluorescence of the free probe was observed to be quenched due to the formation of the probe **70**-Cu^2+^ ensemble **(**Scheme 53**)**. It restored fluorescence by the presence of S^2−^ anions (4 equivalents) only, exhibiting a ‘turn-on’ response. The LOD of **71**-Cu^2+^ towards S^2−^ was found to be 354 nM. Furthermore, Cytotoxicity studies of live cells revealed that the probe showed low toxicity and could be a potent sensor for detecting S^2−^ intracellularly [105] with an excellent cell permeability, thereby showing potential applications in biological systems.

**Scheme 54:**
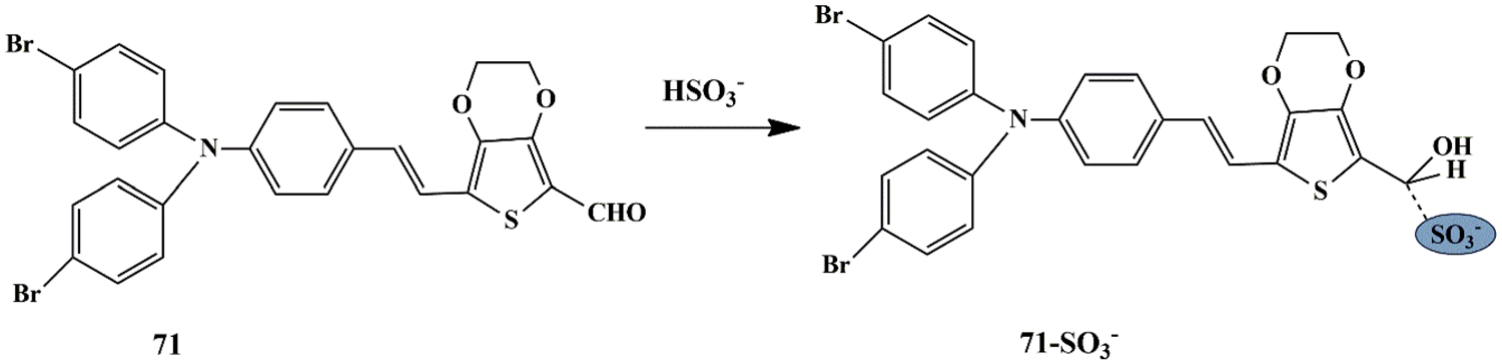
Possible mechanism of probe **71** binding to Bisulphite

Although few sensors have been reported for sulfite anions, dual-channel chemosensors for the detection of hydrogen sulfite are quite limited, and hence as an emerging approach, Xiaohong et al. designed a series of compounds possessing electron-donating thiophene moieties attached to triphenylamine (TPA) with aldehyde groups. Only probe **71**, having ethylene dioxy-thiophene and a thiophene group, could exhibit spectral changes in the UV-Vis spectra along with a notable color change from yellow to colorless for HSO_3_^−^ ions. Furthermore, it was found that probe **71** could selectively detect HSO_3_^−^ ions over other anions, which was evident by the color change from yellowish-green to blue, attributable to the nucleophilic addition of the aldehyde to hydrogen sulfite resulting in aldehyde-hydrogen sulfite adduct [106] (Scheme 54**)**, thereby tuning the ICT efficiency, and this mechanism was corroborated by ESI-MS spectra. The detection limit was determined to be low as 0.9 µM, which was indicative of the higher sensitivity of the probe towards HSO_3_^−^ anions.

### Hypochlorite

Hypochlorite is widely being used in industries and households as an effective cleaning agent to remove stains and whiten clothes, disinfectants, and air fresheners. One of the most potent oxidizing agents is sodium hypochlorite, which is mainly used as a bactericide to treat drinking water and eliminate foul odors. The residual reactive chlorine formed during the disinfection of water can form disinfection by-products such as trihalomethanes, known to be carcinogenic. Additionally, hypochlorite toxicity can induce oxidation damage of the extracellular components, consequently leading to Arthritis, respiratory disorders, cancer. Concerning the danger of ClO^−^ ions, it is crucial to keep a track of hypochlorite ions in aqueous and biological samples.

**Scheme 55:**
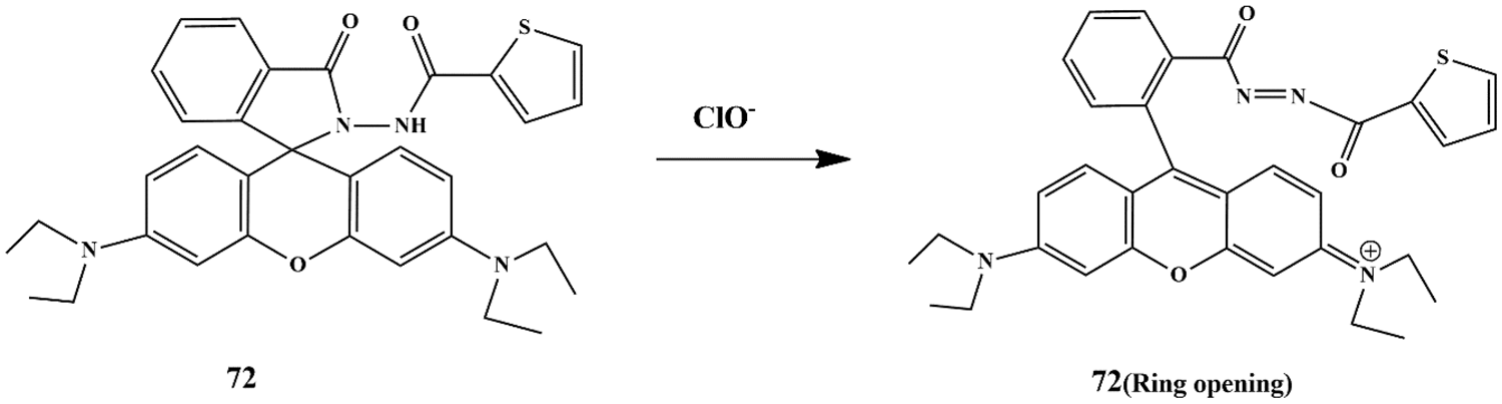
Suggested mechanism of probe **72** in response to ClO^−^ ions

Ying et al. designed a fluorometric and colorimetric probe **72** based on rhodamine-B fluorophore and thiophene-2-carbohydrazide. An instant ‘naked-eye’ color change of the probe in Tris-HCl buffer solution, from colorless to pink, and the appearance of red fluorescence (λ_excitation_ = 365 nm) under UV light, upon the addition of ClO^−^ ions was indicative of the ring-opening reaction of the spirolactam of rhodamine **(**Scheme 55**)**. Besides, a new absorption peak and fluorescence peak at 556 nm and 578 nm emerged, which further suggested that ClO^−^ could persuade a ring-opening reaction of rhodamine moiety, verified by HRMS spectra. Hypochlorite titration experiments were also performed, in which the probe exhibited spectral changes with increasing concentrations of ClO^−^ ions along with deepened color change. It exhibited a fast response time of 10 s with a detection limit of 7 µmol/L. Furthermore, the practical application of the probe was tested for aqueous samples by filter paper strips, which showed a color change upon detection of ClO^−^ ions. It was also observed that probe **72** could be used for fluorescent bioimaging and mitochondrial targeting [107] of HeLa cells, thereby being an effective tool for monitoring ClO^−^ ions.

### Thiocyanate

Thiocyanate is pervasive and abundantly found in the extracellular fluids of mammals and enters into the body primarily from the diet. Thiocyanate serves various applications as additives or starting materials for dye synthesis, chemical tracers and finds applications in textiles and manufacturing industries. However, thiocyanate toxicity symptoms have been related to the nervous system, such as motor impairment, anxiety, thyroid gland disorders such as hypothyroidism, skin, and kidneys. Despite the prevailing circumstances, the development of chemosensors for SCN^−^ detection is minimal.

One successful attempt was the probe **73** [4-methyl-2,6-bis((thiophen-2-ylmethylimino)methyl)phenol], developed by Sudipta et al. for SCN^−^ detection. The absorption spectrum of free probe in H_2_O/CH_3_OH (v/v 1:4, HEPES buffer) solution exhibited two intense peaks at 350 nm, and 450 nm, which gradually diminished and increased respectively, upon the addition of SCN^−^ ions and this is attributable to the CH=N isomerization. Furthermore, the fluorescence emission intensity enhanced along with a blue shift upon SCN^−^ addition and the detection of SCN^−^ was not affected by other bio-relevant anions. The SCN^−^ recognition could be ascribed to the Hydrogen bond formation [108] between probe **73** and SCN^−^ ion and 1:1 stoichiometry were proposed. The probe’s LOD for SCN^−^ was found to be 0.88 µmol/L with a quick response time (<5 min).

**Scheme 56:**
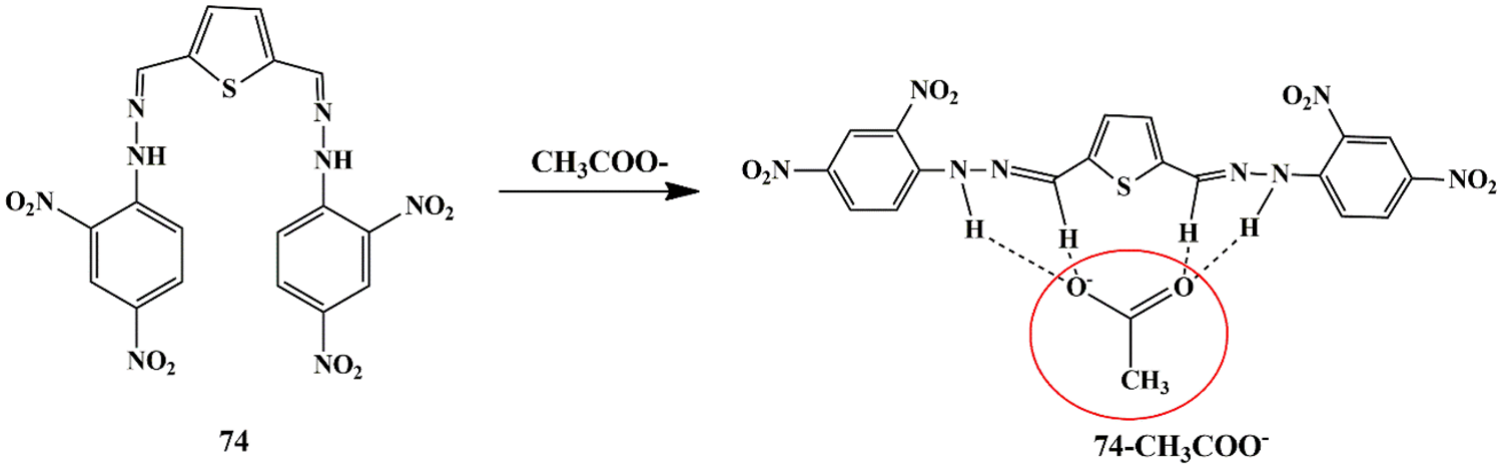
Suggested mechanism of probe **74** in response to Acetate ions

Although diverse, efficient colorimetric sensors have been reported for anion detection and sensing, their applicability towards the real specimens or samples, is still limited. In this regard, Sivalingam et al. synthesized a thiophene-based dihydrazone derivative probe **74** to detect anions in an organic and aqueous medium. Among all anions, the probe exhibited strong affinity towards F^−^, AcO^−^ and H_2_PO_4_^−^ ions in DMSO/H_2_O (v/v 10:0 & v/v 9:1), evident by a visual color change from orange to violet and yellow to brown in aqueous and organic medium, respectively, along with a strong absorption band at 590 nm. Probe **74** gains importance in sensing AcO^−^ in any form such as TBA acetate, transition metal acetate, and sodium acetate in the aqueous medium. The sensing mechanism can be assigned to the hydrogen bond formation between N-H and AcO^−^ ions (Scheme 56**)** with a 1:1 stoichiometry binding mode [109] for all receptor-anion complex. Ultimately, the probe could qualitatively estimate F^−^ and AcO^−^ in real samples such as commercially available toothpaste and vinegar samples by colorimetric technique.

**Scheme 57:**
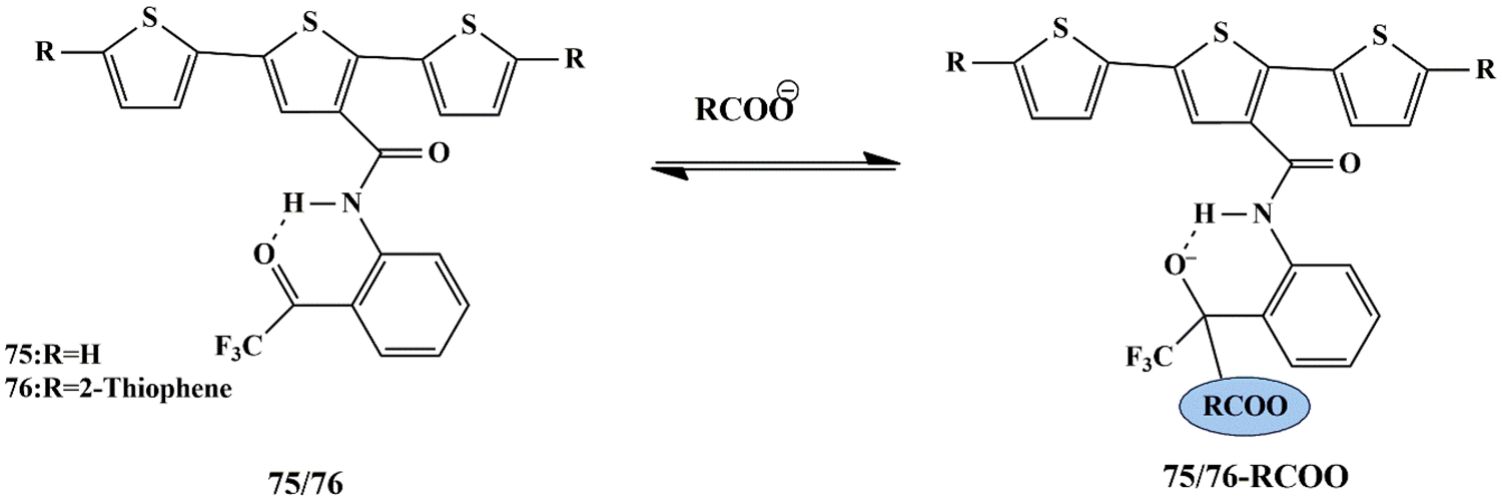
Suggested mechanism of sensor **75/76** in response to Acetate ions

Oligothiophenes have excellent photophysical properties, such as their intrinsic fluorescence and simplistic polymerization properties, due to which it has received considerable attention. Despite their advantageous properties, small efforts have been put forward for the development of oligothiophene-based fluorescent chemosensors. One such great effort was put forth by Dae et al., who reported efficient turn-on oligothiophene-based fluorescent chemosensors (**75** and **76**) for the detection and sensing of carboxylate anions with a binding mode of 1:1 stoichiometry. Both sensors exhibited fluorescence quenching in CH_3_CN due to the n − π* transition of the trifluoroacetophenone group. In contrast, they exhibited remarkable fluorescence enhancement for CN^−^, AcO^−^, F^−^, and H_2_PO_4_^−^, among which the selectivity of probe **75** was highest for AcO^−^, evident by a slight bathochromic shift. The fluorescence enhancement could be accounted for due to Carbonyl adduct formation, supported by H-NMR and F-NMR studies, and the sensing mechanism was through the elimination of n − π* transition and presence of intramolecular Hydrogen bonding [110] (Scheme 57**)** which enhances the conformational rigidity of the adduct, thereby promoting fluorescence enhancement. Therefore, these highly efficient fluorescence turn-on chemosensors could be further explored in biomolecules for Carboxylate anion detection.

Chemosensors have been a powerful tool pertaining to its ease of use, reliability and practical applicability. High selectivity and sensitivity of the chemosensors is achieved by introducing various moieties to the fluorophore core system. In the current review, we have listed out thiophene based fluorescent and colorimetric chemosensors (Table 1) along with their detection of ions, LOD and their applications.

**Table 1:** Different chemosensors with the ions detected, detection limits and applications

Chemosensors	Ions detected	LOD	Applications
**1**	Al^3+^	0.41µM	Used as biocompatible detector for Al^3+^ in Biological systems (HeLA cells)
**2**		8.92 × 10^−8^ M	Evaluation of drinking water quality
**3**	In^3+^	0.61 µM	Sensing of In^3+^
**4**		0.36 µM	−
**5**		−	Sensing of Hg^2+^ in Cosmetics
**6**	Hg^2+^	1.34 µM	
**7**		0.32 µM	−
**8**		2.2 × 10^−8^ M	
**9**		0.01 µM	
**10**	Hg^2+^ and Cu^2+^	28 µM and 7.5 µM	Detection of Hg^2+^ and Cu^2+^ in protein medium (BSA), Dalton’s Lymphoma cells (DL), in contaminated water samples and test strips and explored as coding/decoding fluorescent blue security ink and construction of molecular logic gate.
**11**	Hg^2+^	20 µM	Sensing of Hg^2+^ in partially aqueous medium
**12**	Pb^2+^	−	−
**13**	Cr^3+^	0.4 µM	Sensing of Cr^3+^ on circular test papers
**14**	Pb^2+^ and Hg^2+^	−	−
**15**	Cr^3+^	1.5 × 10^−6^ M	Detection of Cr^3+^ in wastewater samples
**16**	Cr^3+^	2.6 × 10^−6^ M	Detection of Cr^3+^ in live HeLa cells
	Cu^2+^	1.01 × 10^−6^ M	−
	CN^−^	9.3 × 10^−7^ M	−
**17**	Zn^2+^	−	Construction of logic gate for selective potential and biological sensing of Zn^2+^
**18**		1.51 × 10^−7^ M	Designing of molecular logic operation for Zn^2+^ detection
**19**	Zn^2+^ and Hg^2+^	3.7 µM and 4.8 µM	Intracellular probe for dual sensing of ions
**20**	Zn^2+^	−	Biosensing of Zn^2+^ in living cells.
**21**		0.423 nM	Bio-probe for Cu^2+^ detection in bio-imaging and bio-labeling research
**22**	Cu^2+^	1.8 µM	Detection of Cu^2+^ -Fluorescence imaging in vivo
**23**		0.85 µM	Colorimetric detection of ammonia and response to pH effect.
**24**		2.8 × 10^−6^ M	Detection of Cu^2+^ ions in environmental water samples
**25**	Cd^2+^	2.0 × 10^−7^ M	Detection of Cd^2+^ ions in environmental water samples
**26**	Cu^2+^	0.217 µM	Quantification of Cu^2+^ in food and water samples
**27**	CuCl_2_	−	−
**28**	Ag^+^	1.28 × 10^−7^ M	Live-cell fluorescence imaging for Ag^+^ detection and monitoring Ag^+^ ions in natural water samples
**29**		8.18 × 10^−9^ M	−
**30 and 31**	Ca^2+^	^−^	−
**32**	Zr^4+^	16.99 µM	Live cell imaging of Zr^4+^ ions
**33**	Fe^3+^	1.6 × 10^−8^ M.	Construction of field test kit for qualitative analysis of Fe^3+^ and formulation of INHIBIT gate
**34**	Fe^2+^ Ni^2+^ Cu^2+^	2.977 × 10^−6^ M 0.895 × 10^−6^ M 0.593 × 10^−6^ M	Rapid detection of multiple ions
**35**	Fe^3+^ and Fe^2+^	0.51 µM and 1.51 µM	Quantification of Fe^3+^ in water samples
**36**	Fe^3+^	3.74 µmol/L	Detection of Fe^3+^ in living cells with good membrane permeability and less toxicity
**37**	Hg^2+^ and Fe^3+^	3.52 × 10^−8^ M and 5.0 × 10^−8^ M	Analytical quantification of Hg^2+^ and Fe^3+^ in water samples
**38**	Co^2+^ and Cu^2+^	0.19 µM for Co^2+^	Determination of Co^2+^ and Cu^2+^ in drinking and tap water samples
**39**		0.88 µM and 0.14 µM	−
**40 and 41**	Pd^2+^	49 × 10^−9^ M and 44 × 10^−9^ M	Detection of Pd^2+^ using TLC strips
**42**		0.89 µM	Qualitative detection of CN^−^ ions in drinking and mineral water using test strips
**43**		44.6 µM	Selective detection of CN^−^ by fluorescent turn-on method
**44**		0.32 µM	Real-time monitoring of CN^−^ in aqueous media and also employed as DNA-detection agent.
**45**	CN^−^	−	Biomedical applications for sensing CN^−^ at lower concentrations.
**46**		4.24 × 10^− 8^ M	Real-time monitoring of CN^−^ ions in aqueous solution and fluorescence imaging of live cells.
**47**		51 nM	Detection of low levels of CN^−^ in drinking water and bio-imaging to detect these ions
**48 and 49**		0.019 µM and 0.02 µM	−
**50**	F^−^	−	Detection of F^−^ in the presence of competing anions.
**51**	CN^−^, F^−^ and AcO^−^	−	Ratiometric sensing of anions
**52 and 53**	F^−^ and CN^−^	0.04 ppm and 0.07 ppm	Solid state detection ability of F^−^ and CN^−^ using test strip analysis
**54 and 55**	F^−^	−	−
**56**		6.9 × 10^−7^ M	Real-time monitoring of F^−^ ions by construction of logic gate and molecular switches.
**57**		2.02 × 10^−7^ M	Detection of F^−^ by test-strip method
**58 and 59**		−	Potential luminescent chemosensors
**61, 62, 63**		−	Promising candidates for F^−^ detection
**64**	I^−^	1.7 × 10^−7^ M	Qualitative analysis of I^−^ in aqueous samples
**65**	F^−^, Cl^−^, Br^−^, I^−^	−	−
**66**	I^−^	−	DNA quantification by the formation of nanostructured networks
**67**	HSO_3_^−^	−	Detection of exogenous and endogenous HSO_3_^−^ ions *in vivo*
**68 and 69**		−	Detection of HSO_3_^−^ in water samples
**70**	S^2−^	354 nM	Efficacious bio probe for detecting S^2−^ intracellularly
**71**	HSO_3_^−^	0.9 µM	−
**72**	ClO^−^	7 µmol/L	Employed in fluorescent bioimaging and mitochondrial targeting of HeLa cells
**73**	SCN^−^	0.88 µmol/L	−
**74**	F^−^, AcO^−^ and H_2_PO_4_^−^	−	Qualitative estimation of F^−^ and AcO^−^ in toothpaste and vinegar
**75 and 76**	RCOO^−^	−	These chemosensors could be further explored in biomolecules for Carboxylate anion detection.

## Conclusions

There is ever-growing research on the design, synthesis, and improvement in the sensitivity and detection of the various ions in the environmental and biological systems. Thiophene-based fluorometric probes have become an essential tool in detecting anions, cations, and neutral molecules because of their significant characteristics such as extended emission and absorption wavelengths, vast absorption co-efficient, and more fluorescence quantum yield. Consideration of the reviewed data shows that thiophene moiety in combination with other heterocycles has achieved significant interest. The sensing of molecules can be done when thiophene is used as a fluorophore moiety or a signaling unit to behave as a fluorescent “Turn off”’ or “Turn On” chemosensor, when combined with other heterocycles, during the detection of analytes of interest. Although, the substitution needs to be carefully selected in order to obtain excellent results as well as take the benefit of the optical and chelating properties of the probe. The mechanism of sensing of various cations and anions and the reversibility of the interaction mechanism has also been reviewed.
